# Profiling of kidney vascular endothelial cell plasma membrane proteins by liquid chromatography–tandem mass spectrometry

**DOI:** 10.1007/s10157-012-0708-1

**Published:** 2012-12-11

**Authors:** Zan Liu, Bo Xu, Masaaki Nameta, Ying Zhang, Sameh Magdeldin, Yutaka Yoshida, Keiko Yamamoto, Hidehiko Fujinaka, Eishin Yaoita, Masayuki Tasaki, Yuki Nakagawa, Kazuhide Saito, Kota Takahashi, Tadashi Yamamoto

**Affiliations:** 1 Department of Structural Pathology, Institute of Nephrology, Graduate School of Medical and Dental Sciences, Niigata University, Niigata, Japan; 2 Division of Urology, Department of Regenerative and Transplant Medicine, Graduate School of Medical and Dental Sciences, Niigata University, 1-757 Asahimachi-Dori, Chuo-Ku, Niigata, 951-8510 Japan; 3 Department of Physiology, Faculty of Veterinary Medicine, Suez Canal University, Ismailia, Egypt

**Keywords:** Vascular endothelial cell plasma membrane, Cationic colloidal silica nanoparticles, Proteomic analysis, Deltex 3-like

## Abstract

**Background:**

Vascular endothelial cells (VECs) play crucial roles in physiological and pathologic conditions in tissues and organs. Most of these roles are related to VEC plasma membrane proteins. In the kidney, VECs are closely associated with structures and functions; however, plasma membrane proteins in kidney VECs remain to be fully elucidated.

**Methods:**

Rat kidneys were perfused with cationic colloidal silica nanoparticles (CCSN) to label the VEC plasma membrane. The CCSN-labeled plasma membrane fraction was collected by gradient ultracentrifugation. The VEC plasma membrane or whole-kidney lysate proteins were separated by sodium dodecyl sulfate polyacrylamide gel electrophoresis and digested with trypsin in gels for liquid chromatography–tandem mass spectrometry. Enrichment analysis was then performed.

**Results:**

The VEC plasma membrane proteins were purified by the CCSN method with high yield (approximately 20 μg from 1 g of rat kidney). By Mascot search, 582 proteins were identified in the VEC plasma membrane fraction, and 1,205 proteins were identified in the kidney lysate. In addition to 16 VEC marker proteins such as integrin beta-1 and intercellular adhesion molecule-2 (ICAM-2), 8 novel proteins such as Deltex 3-like protein and phosphatidylinositol binding clathrin assembly protein (PICALM) were identified. As expected, many key functions of plasma membranes in general and of endothelial cells in particular (i.e., leukocyte adhesion) were significantly overrepresented in the proteome of CCSN-labeled kidney VEC fraction.

**Conclusions:**

The CCSN method is a reliable technique for isolation of VEC plasma membrane from the kidney, and proteomic analysis followed by bioinformatics revealed the characteristics of in vivo VECs in the kidney.

**Electronic supplementary material:**

The online version of this article (doi:10.1007/s10157-012-0708-1) contains supplementary material, which is available to authorized users.

## Introduction

Vascular endothelial cells (VECs) are known to play important roles in the exchange of oxygen and nutrients with carbon dioxide and metabolites in the microenvironment of organs or tissues. However, apart from this general role, VECs also have organ- or tissue-specific functions [1]. Angiotensin-converting enzyme has higher activity in lung VECs than in VECs in other organs [2], suggesting that VECs differ among tissues and organs. The characteristics of VECs have been extensively studied in vitro [3]. However, the in vivo roles of VECs in tissues and organs remain poorly understood. In fact, once cells are isolated from organs or tissues and grown in culture media, their appearance, structure, and protein expression can change dramatically, leading to phenotypic changes of VECs [4, 5].

VECs have also been demonstrated to play pivotal roles in numerous diseases, such as cancer [6] and diabetes [7]. In the kidney, processes related to injuries or transplant rejection take place on the surface of VECs. Sufficient knowledge about the characteristics of VECs is thus essential to more clearly understand the pathogenesis of kidney diseases.

A recent study comparing a comprehensive mass spectrometry (MS)-based proteome with an antibody-based proteome of single type cultured cells demonstrated that most cell-specific or unique proteins are localized at the plasma membrane or in association with the membrane [8]. These results suggested that the specific functions of cells depend largely on their plasma membrane protein profile. MS-based proteomics studies have provided unprecedented information on the protein expression of organs or tissues, as well as the protein components of subcellular multimolecular complexes [9, 10]. Profiling the VEC plasma membrane proteome has proven to be challenging since the overall levels of proteins are low in organs and tissues, making identification on proteomics analysis challenging. In addition, cells and their organelles are dynamic structures, constantly shuffling proteins between compartments [11]. Therefore, enrichment and purification of VEC plasma membrane are required for proteomic analysis. The cationic colloidal silica nanoparticle (CCSN) procedure was introduced to selectively collect VEC plasma membrane proteins from organs. This procedure is based on ionic interactions of negatively charged plasma membrane with positively charged nanoparticles and involves intravascular perfusion and collection of particle-labeled VEC plasma membrane [12, 13]. Enrichment of plasma membrane proteins from rat lung VECs was successfully performed, and 81 % of proteins were classified as plasma membrane proteins [5].

This study was designed to profile the kidney VEC plasma membrane and entire kidney proteome by means of the CCSN technique and liquid chromatography–tandem mass spectrometry (LC–MS/MS). Our results confirm the efficiency of these methods for isolation of VEC plasma membrane and demonstrate some characteristic features of kidney VECs.

## Materials and methods

### Animals

Male 8-week-old Wistar rats (Charles River) were used in this study. The use of these animals in this study was approved by the Ethics Committee and Animal Committee of Niigata University School of Medicine.

### CCSN preparation

CCSN was prepared as follows: 9 ml of colloidal silica beads (Nalco 1060, diameter 60 nm; Ondeo Nalco Company, USA) were mixed with 3 ml of aluminum chlorohydroxide complex solution (350 mg) (Reheis Chemical Company, USA) for 2 min at maximum speed in a blender (Nihonseiki Kaisha, Ltd., Japan), as described previously [13]. The mixture was then incubated while stirring in a water bath at temperature of 80 °C for 30 min. The pH of the colloidal silica bead solution was adjusted to 5.0 with 1 N NaOH, and the solution was incubated for 24 h. The solution was then diluted to 30 % with distilled water and stored at 4 °C. Immediately before use, the silica bead solution was further diluted to 6 % with 140 mM sorbitol and 20 mM 2-(*N*-morpholino)ethanesulfonic acid hydrate (MES, Sigma-Aldrich Co., USA) solution.

### Perfusion of CCSN and isolation of kidney VEC membrane

After anesthetizing the rats with ether, the abdominal aorta was cannulated just below the left renal artery, and the following blood vessels were clipped: the inferior vena cava just below the hepatic vein, the abdominal aorta below the superior mesenteric artery, the abdominal aorta at the puncture site, and the inferior vena cava between the left and right renal veins. Then, a hole was made in the left renal vein to allow outflow of perfusates. The flow rate of all solutions was maintained at approximately 2–3 ml/min. The left kidneys were perfused sequentially with the following solutions: (1) phosphate-buffered saline (PBS) for 3 min to remove blood from the vascular bed, (2) ice-cold PBS for 5 min to reduce the temperature of the perfused kidneys to 10–15 °C, (3) MES-buffered saline (pH 6.0) for 2 min to reduce the pH of the vascular bed, (4) 6 % CCSN solution for 3 min to label the surface of VECs, (5) MES for 1 min to wash out unbound CCSN, (6) 1 % sodium polyacrylate in MES for 2 min to cross-link CCSN and VEC plasma membrane, and (7) 4-(2-hydroxyethyl)-1-piperazineethanesulfonic acid (HEPES) buffer [25 mM HEPES, 250 mM sucrose, 1 mM ethylenediaminetetraacetic acid (EDTA), pH 8.0] for 3 min to flush the vasculature.

After perfusion, the left kidney was removed and minced with a razor blade in a plastic dish at 4 °C and then placed in 5 ml HEPES buffer. Homogenization was carried out for 2 min at 14,000 rpm (Polytron PT1200; Kinematica, AG, Switzerland). The homogenate was filtered through a 40-μm nylon monofilament net, and the filtrate was then fractionated by Nycodenz (Axis-Shield plc, Scotland) gradient centrifugation as follows: the filtered homogenate was diluted with an equal volume of 1.02 g/ml Nycodenz, and the total volume of 5 ml mixture was layered onto a 55–70 % Nycodenz gradient by placing 2.0 ml of 70 %, 1.5 ml of 65 %, 1 ml of 60 %, and 1 ml of 55 % Nycodenz in a 12-ml centrifuge tube. The tube was topped off with HEPES buffer and centrifuged at 15,000 rpm for 30 min at 4 °C in a swinging bucket rotor (P40ST; Hitachi High Technology, Japan). After centrifugation, the supernatant was removed, and the CCSN-labeled membrane fraction was collected at the bottom as a pellet. The pellet was then resuspended in 1 ml MBS. Then, an equal volume of 1.02 g/ml Nycodenz was added to the solution, and a second centrifugation was performed at 30,000 rpm for 60 min at 4 °C (CP80β; Hitachi High Technology, Japan), using a 80–60 % Nycodenz gradient (1.5 ml of 80 % and 0.7 ml of 75, 70, 65, and 60 % Nycodenz). The CCSN-coated membrane was collected as a pellet and was washed in 1 ml MBS buffer in a microfuge tube at 14,000*g* for 30 min. The CCSN was resuspended in 100 μl of 2 % sodium dodecyl sulfate (SDS) in 50 mM Tris buffer (pH 7.4) and sonicated at 50 Hz for 30 s to detach the CCSN from the VEC membrane. The suspension was heated at 100 °C for 5 min to solubilize proteins, and the silica was separated by centrifugation at 14,000*g* for 15 min.

### Histological examination

After perfusion of the CCSN beads, parts of the kidneys were fixed in 10 % formalin and embedded in paraffin for light-microscopic examination. Small kidney blocks of approximately 1 mm^3^ were fixed in 2.5 % glutaraldehyde in 0.1 M phosphate buffer (pH 7.4) overnight for electron microscopy. Sections of the kidneys were stained with periodic acid-methenamine (PAM) to demonstrate binding sites of the CCSN beads by light microscopy. The glutaraldehyde-fixed blocks were postfixed for 1 h in 1 % OsO_4_ in 0.1 M phosphate buffer and then embedded in epoxy resin. Ultrathin sections were cut, stained with uranyl acetate and lead citrate, and observed under a transmission electron microscope (H-600A; Hitachi High Technology).

### Immunoblotting

Protein concentrations of the samples were determined by Lowry’s method, and 10 μg protein of each sample was separated on 10 % sodium dodecyl sulfate polyacrylamide gel electrophoresis (SDS-PAGE) gels. The electrophoresed proteins were transferred onto polyvinylidene fluoride (PVDF) membranes and incubated with primary antibodies overnight at 4 °C, followed by peroxidase-labeled anti-mouse immunoglobulin G (IgG) antibody (1:1,000; Dako Denmark A/S, Denmark). Immunoreactive proteins were visualized using an enhanced chemiluminescence detection system (ECL Plus; GE Healthcare, UK). Primary antibodies used in this study were as follows: monoclonal anti-caveolin-1 antibody (sc-53564; Santa Cruz Biotechnology, USA) for identification of VEC plasma membrane fraction, monoclonal anti-lysosomal-associated membrane protein 1 (LAMP1) antibody (sc-17758; Santa Cruz Biotechnology) for identification of lysosomal vesicle fraction, monoclonal anti-cytochrome *c* antibody (BD Biosciences, USA) for identification of mitochondria fraction, and monoclonal anti-ras-related nuclear protein (Ran) antibody (BD Biosciences) for identification of nucleus fraction.

### Mass spectrometry and protein identification

**Fig. 1:**
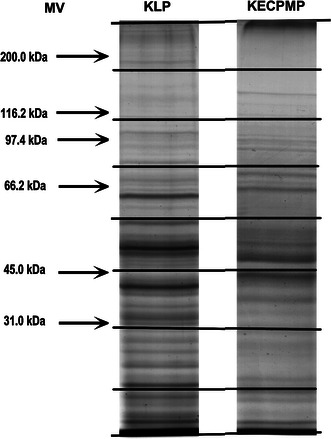
SDS-PAGE analysis of proteome preparations from KECPMP and KLP. Samples containing 15 μg proteins were separated on a 10 % polyacrylamide gel, and proteins were visualized by staining with Coomassie Brilliant Blue R-250. The respective protein separation lanes were manually cut into 8 equal slices (6.5 mm/slice)

Each of three samples of kidney endothelial cell plasma membrane proteins (KECPMP) collected by the CCSN method and, additionally, three samples of kidney lysate protein (KLP) were separated by 10 % SDS-PAGE gels (15 μg each), stained with Coomassie Brilliant Blue R-250, cut into 8 slices per lane, and subjected to in-gel trypsin digestion as described previously (Fig. 1) [14].

Mass-spectrometric analysis was performed by using an ion-trap mass spectrometer (Agilent 6300 series LC/MSD XCT; Agilent Technologies, Hachioji, Japan) online coupled with a nanoflow high-performance liquid chromatography (HPLC) system (Agilent 1100) equipped with a trap column (ZORBAX 300SB-C18, 5 μm, 0.3 × 5 mm; Agilent) and a separation column (ZORBAX 300SB-C18, 3.5 μm, 0.075 × 150 mm; Agilent). Mobile phases used were: A, 0.1 % formic acid, 2 % methanol; B, 0.1 % formic acid, 98 % methanol. Tryptic peptides were applied and eluted by 2–70 % B in 120 min, followed by 70 % B isocratic run for 5 min, and subsequent 100 % B isocratic run for 10 min at flow rate of 300 nl/min. The mass spectrometer was operated in positive mode in the scan range of 350–2,200 *m*/*z*, signal-to-noise ratio ≥25. The three most intense peaks with charge state ≥2 were selected from each survey scan in data-dependent mode.

For protein identification, raw data were processed to generate MS/MS peak lists (Mascot generic file) by using Data Analysis software for 6300 series ion-trap LC/MS (version 3.4). The MS/MS ion search was performed by Mascot Daemon (version 2.2.01) to search against the International Protein Index (IPI) rat protein database (version 3.70). Peptide modification settings were: fixed modification, carbamidomethylation on Cys; variable modifications, oxidation on Met, deamidation on Asn and Gln. The peptide and fragment mass tolerances were set at ±2.5 and 0.7 Da, respectively. Maximum missed cleavage of 2 was allowed. The “require bold red” option was activated to remove redundancy. The significance threshold was adjusted to give a false-discovery rate (FDR) <1 %, which was calculated on the basis of the number of peptide matches against a decoy database. Proteins identified with matched peptides exceeding the “identity threshold” are reported as identified proteins.

### Bioinformatics analysis

Distributions in subcellular location and molecular function were assigned to each protein based on UniProt/GO (http://www.uniprot.org, http://www.geneontolgy.org) and also by manually searching the literature. Functional enrichment analyses of cellular components, molecular functions, and biological processes were performed via the FatiGO analytic tool (http://www.fatigo.org). In the enrichment analysis, modified Fisher’s exact tests were used for statistical analysis. The significantly (*p* value <0.05) enriched GO categories are presented. Each annotated function was assigned a *Z* score to measure whether a given function or process was significantly overrepresented in our VEC plasma membrane proteome relative to the public databases.

### Deltex 3-like immunohistochemical and immunofluorescence analysis

For immunohistochemical analysis, kidney tissues were fixed in methyl Carnoy’s solution and embedded in paraffin. The paraffin-embedded tissues were sectioned at thickness of 4 μm, dewaxed, and incubated sequentially with rabbit anti-human Dll3 antibody (Sigma-Aldrich Co., USA) for 1 h and horseradish peroxidase-conjugated goat anti-rabbit immunoglobulins at 37 °C for 1 h. The peroxidase reaction was visualized using 0.5 mg/mL of 3′-diaminobenzidine tetrahydrochloride-0.01 % hydrogen peroxide as substrate.

For immunofluorescence, frozen blocks were sectioned at thickness of 3 μm. Rabbit monoclonal anti-Dll3 in combination with mouse monoclonal anti-caveolin-1 antibody were applied as primary antibodies for double-labeled immunostaining. After washing with PBS, the sections were stained with fluorescein isothiocyanate-conjugated goat anti-rabbit IgG, and subsequently with Texas-Red-conjugated anti-mouse immunoglobulins. Immunofluorescence of the stained sections was observed with a microscope (BX50; Olympus, Tokyo, Japan).

## Results

### Labeling of CCSN on the surface of VECs in the kidney

**Fig. 2:**
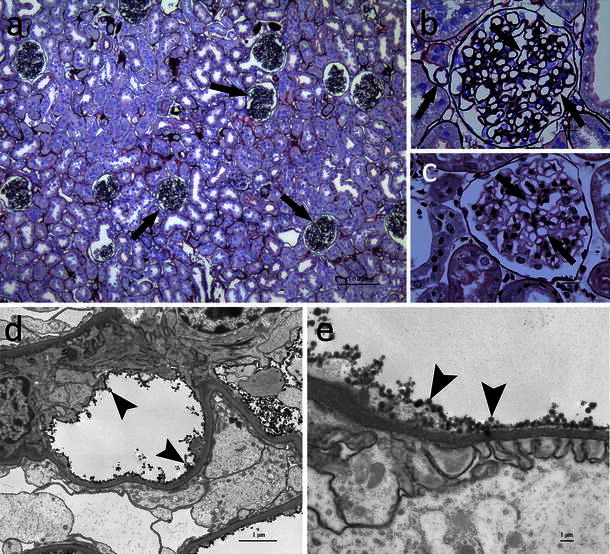
Histological micrograph of a rat kidney perfused with CCSN (**a**–**e**). The *thick arrow* points to the CCSN-coated vascular endothelium. Overview showing the PAM staining confirmed intense and exclusive labeling of CCSN on the surface of VECs in the kidney. No labeling was observed in other sites of the kidneys (**a**). Intense staining along the inner surface of the renal vasculature was observed in the kidneys. A nanoparticle is attached to the capillary (**b**). CCSN labeling was negative in rat kidney sections as negative control (**c**). Transmission electron micrograph of rat kidney perfused with silica beads. Overview showing the CCSN-coated microvasculature (**d**). Specificity of the labeling procedure to an individual capillary at different magnifications (**e**)

To confirm that CCSN bound to cell surface membrane of VECs in the kidney, the rat kidneys removed after the perfusion of CCSN were examined by light and electron microscopy. Intense staining of CCSN along the surface of the renal vasculature was observed on the PAM-stained kidney sections, indicating universal labeling of CCSN on VECs; no labeling was observed in other sites of the kidneys (Fig. 2a–c). Electron microscopy also demonstrated CCSN on the surface of peritubular and glomerular capillaries and other blood vessels (Fig. 2d, e).

### Immunoblotting analysis

**Fig. 3:**
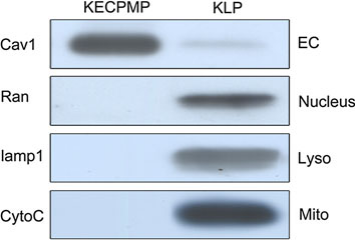
Western blot analysis of kidney VEC membrane and kidney lysate samples for quality control. Proteins (10 μg) were separated by SDS-PAGE, transferred to PVDF membrane, and immunoblotted with antibodies to the indicated proteins. Enrichment of membrane protein Caveolin-1 (*Cav1*) is found in the kidney VEC membrane fraction without contamination by intracellular components. Cytochrome *c* (*CytoC*) is a marker for mitochondria, Ran for nuclei, and LAMP1 (*lamp1*) for lysosomes

The purity of VEC plasma membrane fraction isolated by the CCSN method was examined by Western blotting using antibodies against organelle-specific marker molecules: caveolin-1 for VEC plasma membrane, cytochrome *c* for mitochondria, Ran for nucleus, and LAMP1 for lysosomes. An intense band was immunoblotted with anti-caveolin-1 antibody in the CCSN-labeled protein fraction. No bands were demonstrated in the fraction on Western blotting with antibodies against cytochrome *c*, Ran, or LAMP1 (Fig. 3). These results indicated that the VEC membrane proteins are highly enriched in the CCSN-labeled protein fraction and that no other subcellular organelles were included.

### LC–MS/MS analysis and protein classification

**Fig. 4:**
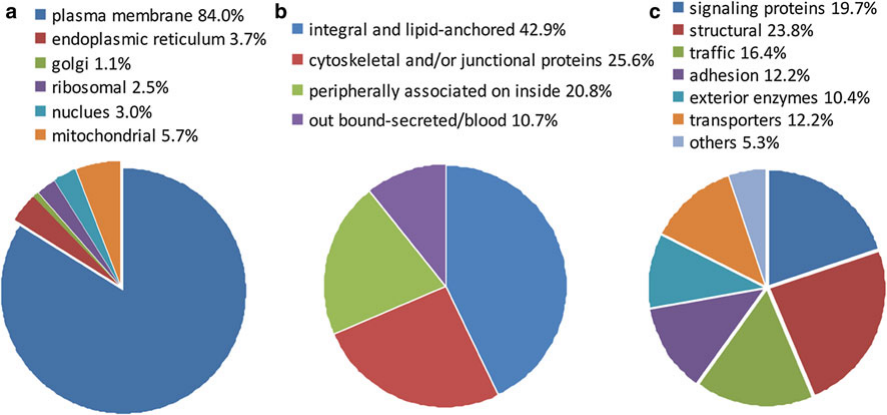
Classification of proteins identified in rat kidney VEC plasma membrane. The expected primary subcellular localization of the characterized proteins (**a**), subclasses of plasma membrane proteins (**b**), and functional characterization of the plasma membrane proteins (**c**)

After merging data, 1,205 proteins and 582 proteins were respectively identified in whole kidney lysate and kidney VEC plasma membrane by Mascot search as high-confidence proteins (see Online Resources 1, 2). In the VEC plasma membrane proteome, 399 (71 %) proteins were categorized as characterized proteins and 183 (29 %) were categorized as yet-to-be-characterized proteins on GO/UniProt annotation analysis. The yet-to-be characterized proteins included entries from genes of unknown functions or hypothetical proteins. Among the characterized proteins, 335 (84.0 %) proteins were classified as plasma membrane proteins, and smaller numbers of proteins were classified as proteins in ribosomes (2.5 %), endoplasmic reticulum (ER) (3.7 %), mitochondria (5.7 %), Golgi apparatus (1.1 %), and nuclei (3.0 %) (Fig. 4a).

The 335 plasma membrane proteins were further classified according to their interactions, orientation, and structure in the membrane. A total of 143 proteins (42.9 %) corresponded to integral or lipid-anchored membrane proteins, 86 proteins (25.6 %) corresponded to cytoskeletal and/or junctional proteins, 70 proteins (20.8 %) corresponded to peripherally associated on inside proteins, and 36 proteins (10.7 %) corresponded to externally bound-secreted/blood proteins (Fig. 4b).

The plasma membrane proteins were also classified into several categories according to GO/UniProt functional annotation: 66 (19.7 %) signaling proteins, 80 (23.8 %) structural proteins, 55 (16.4 %) trafficking proteins, 41 (12.2 %) adhesion, 34 (10.4 %) exterior enzymes, 41 (12.2 %) transporters, and 18 (5.3 %) other proteins (Fig. 4c).

### Enrichment analysis of cellular components, biological processes, and molecular functions

**Fig. 5:**
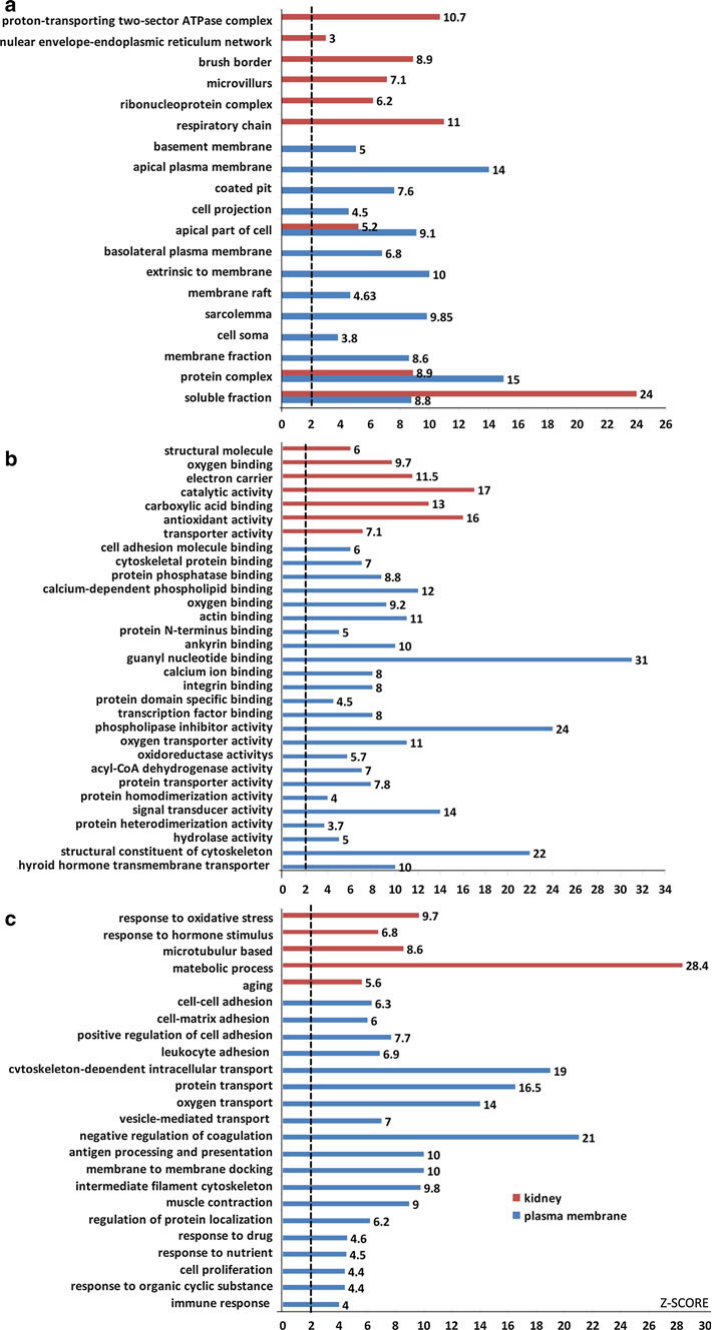
Enriched cellular components, biological processes, and molecular functions in kidney and kidney VEC plasma membrane proteome. The overrepresentation of each category was determined by *Z* score (≥2). All general categories in cellular components, molecular functions, and biological processes included in these data are listed in this figure. Each general category is organized by GO algorithms, and only selected categories are shown: enriched cellular components in kidney and kidney VEC plasma membrane proteomes (**a**), enriched biological processes in kidney and kidney VEC plasma membrane proteomes (**b**), and enriched molecular functions in kidney and kidney VEC plasma membrane proteomes (**c**)

To assess the enrichment degree of plasma membranes and to explore overrepresented biological functions associated with the plasma membrane proteins, the web-based program FatiGO was used to characterize potential biological functions in the rat kidney VEC plasma membrane proteome. Then, the significance of enrichment of each functional category was determined by *Z* score. The VEC plasma membrane proteome was also compared with the rat whole-kidney proteome. On FatiGO/GO ontology analysis, 460 proteins of the VEC plasma membrane dataset and 1,205 proteins of the whole-kidney dataset were matched to the FatiGO rat knowledge database. With respect to cellular components, 13 cellular component terms were overrepresented in the VEC plasma membrane, including apical plasma membrane (*Z* > 14), basolateral plasma membrane (*Z* > 6), and basement membrane (*Z* > 5). In contrast, 9 terms were overrepresented in the whole-kidney proteome, including respiratory chain (*Z* > 11), ribonucleoprotein complex (*Z* > 6), and microvillus (*Z* > 7) (Fig. 5a).

Similar to the cellular components, FatiGO algorithms were used to classify and investigate the molecular functions and biologic processes of all proteins in these two proteomes; 24 overrepresented terms were found in the molecular function category in VEC plasma membrane fraction. Most of these terms can typically be associated with the plasma membrane, including binding, signal transducer activity (*Z* > 14), and structural constituent of cytoskeleton (*Z* > 22). In the whole kidney, 7 terms were significantly enriched, including structural molecule (*Z* > 6) and transporter activity (*Z* > 7) (Fig. 5b). In biological processes, 19 terms were significantly enriched in the VEC plasma membrane fraction, including cell–cell adhesion (*Z* > 6) and protein transport (*Z* > 16). In the whole-kidney lysate, 5 terms were significantly enriched, such as metabolic process (*Z* > 28) and response to hormone stimulus (*Z* > 9) (Fig. 5c).

**Fig. 6:**
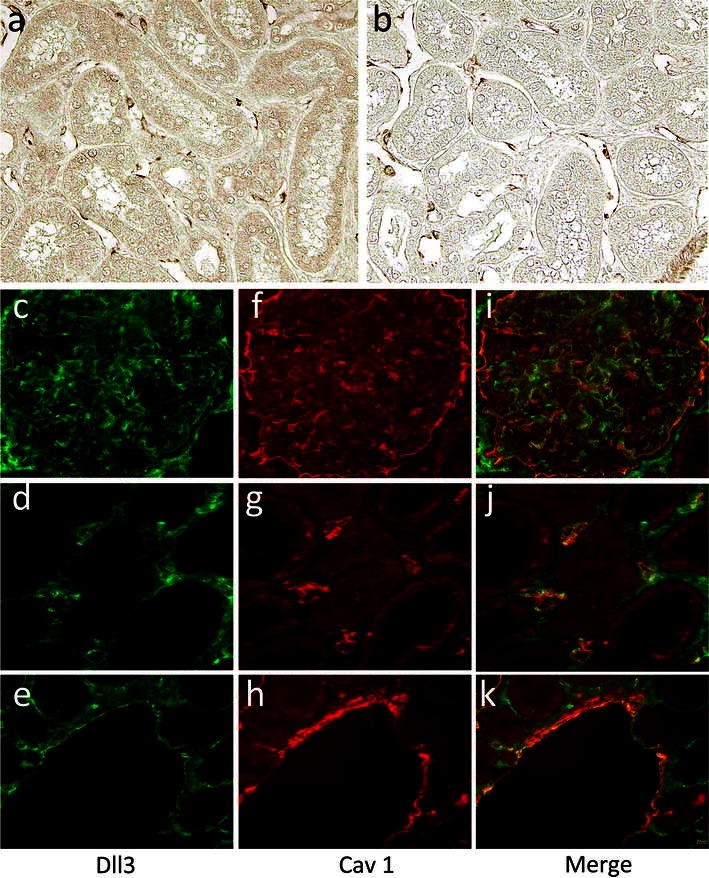
Immunohistochemical validation of protein expression using antibodies to Deltex 3-like in normal kidney tissue. Significant staining was observed in the VEC membrane of kidney (**a**, **b**). Double-labeled immunofluorescence microscopy was conducted using anti-Deltex 3-like antibody (**c**–**e**) and anti-caveolin-1 antibody (**f**–**h**). Their merged image is also shown (**i**–**k**)

In this study, we identified 16 proteins known to be VEC marker membrane proteins in the CCSN-labeled plasma membrane fraction (Table 1). In addition, 8 proteins not previously reported to be VEC proteins in the kidney were confirmed to be VEC proteins on the basis of the immunolocalization of these orthologous proteins in the kidney as demonstrated in the Human Protein Atlas (Table 2). Among these proteins, we focused on Deltex 3-like (Dll3), because growing evidence suggests that Dll families (Dll1, Dll3, and Dll4) act as Notch signaling ligands and participate in regulation of vasculogenesis and angiogenesis by modulating Notch signaling pathway [24]. This has not been demonstrated previously in VECs of any organ. We then investigated the actual subcellular location of Dll3 by immunohistochemical and double-labeled immunofluorescence techniques using human kidney sections and anti-Dll3 antibody. The results of immunohistochemical analysis showed Dll3 expression in VECs specifically in kidney (Fig. 6a, b). Immunofluorescence microscopy showed co-localization of Dll3 and caveolin-1 to glomerular capillaries, veins, and arteries, but not to tubules elsewhere (Fig. 6c–k).

**Table 1:** VEC membrane marker proteins identified in the VEC membrane fraction

Prot Desc	Accession No.^a^	Prot_Matches	Prot_Score^b^	Prot_Sequence	Mass cover (%)
Dipeptidyl peptidase 4	IPI00208422	35	297	88,774	17.5
Carbonic anhydrase 3	IPI00230788	32	434	29,698	12.4
Sodium/potassium-transporting ATPase subunit alpha-1	IPI00326305	28	613	114,293	17.6
Integrin alpha-1	IPI00191681	16	149	91,687	12.9
Integrin beta-3	IPI00198695	11	118	90,066	10.9
Integrin beta-2	IPI00360541	9	113	87,955	13.7
Epidermal growth factor receptor	IPI00212694	7	47	138,225	11.7
Scavenger receptor class B type 2	IPI00464469	7	56	56,705	7.3
Von Willebrand factor A domain-containing protein 5A	IPI00400616	6	49	92,280	7.9
5′-Nucleotidase	IPI00204348	5	84	64,384	8.5
Aminopeptidase N	IPI00230862	5	88	109,779	6.4
Aquaporin-1	IPI00327202	4	116	29,066	7.8
Intercellular adhesion molecule-2	IPI00372952	3	71	31,641	9.7
Endomucin	IPI00372732	2	56	26,614	4.6
CD59 glycoprotein	IPI00195173	1	47	14,465	5.2
Annexin 5	IPI00471889	1	81	35,779	3.7

^a^Accession number of IPI protein database

^b^Score provided from Mascot search engine for protein identification (calculated by MudPIT scoring of Mascot)

**Table 2:** Novel proteins identified in the VEC membrane fraction

Prot_Desc	Accession No.	Prot_Matches	Prot_Sequence	Score cover (%)
Fermt2 RCG61183, isoform CRA_b	IPI00362106	15	140	14.9
Signal recognition particle 72-kDa protein	IPI00763992	11	49	10.0
Tubulin alpha-4A chain	IPI00362927	7	98	9.4
PICALM	IPI00194959	6	111	9.0
ATP-binding cassette, sub-family E (OABP), member 1	IPI00193816	5	47	6.3
Receptor-type tyrosine-protein phosphatase C	IPI00231601	5	75	6.5
Deltex 3-like	IPI00763877	3	66	3.3
Dihydropyrimidinase-related protein 2	IPI00870112	1	51	2.1

## Discussion

VECs have been demonstrated to play important roles in microenvironments of organs or tissues in physiological as well as pathological conditions. The kidney has a complex vascular network, which is related to the functions of the kidney and the development and progression of kidney diseases or the rejection of renal transplants. Plasma membrane proteins have been reported to have important roles in the functions of cells. Therefore, knowledge about VEC plasma membrane proteins in the kidney is essential to understanding renal VEC functions. However, comprehensive in vivo studies of kidney VEC plasma membrane have been precluded by difficulty in isolating VECs from the kidney and the low abundance of VEC plasma membrane proteins. The CCSN method was introduced by Chaney and Jacobson [15] to isolate the VEC plasma membrane in vivo from rat lungs, utilizing the electrostatic attachment of CCSN to negatively charged plasma membrane. Studies showed proteomes of VEC plasma membrane proteins in rat lungs with >20-fold enrichment of VEC plasma membranes relative to total homogenate/lysate, and 81 % of identified proteins were plasma membrane-associated proteins [5]. Using this technique, we first isolated VEC plasma membrane proteins from the kidney. Quality control by Western analysis and functional annotation/enrichment analysis demonstrated that kidney VECs were highly enriched by our methods. Consistent with the findings of previous studies [5], 84 % of characterized proteins were classified as plasma membrane proteins in our study. Moreover, the number of identified proteins in our study was much higher than that of VEC plasma membrane proteins collected by the biotin–avidin method [16].

Among 335 plasma membrane proteins identified in the present study, several VEC membrane marker proteins were included. ICAM-2 is a type I transmembrane glycoprotein that is constitutively expressed in VECs [17] and mediates adhesive interactions between cells involved in antigen-specific immune response, natural killer (NK)-cell-mediated clearance, lymphocyte recirculation, and other cellular interactions.

Integrin alpha-1 is known to be expressed in both leukocytes and endothelium and to participate in cell adhesion as well as cell-surface-mediated signaling, involving leukocyte adhesion to VEC, migration into the subendothelial matrix, and neural migration [18]. Von Willebrand factor (vWf) was also identified in this study, which is well known to be involved in hemostasis and is also a blood type ABO antigen-carrying protein. It exists as a multimeric plasma glycoprotein and a membrane-bound protein in VECs and megakaryocytes. Immunofluorescence microscopy demonstrated its localization in VECs of the human kidney [19].

Eight novel proteins, not previously reported in kidney VEC, were identified as plasma membrane proteins. One of them was Dll3, which has been reported to participate in the Notch signaling pathway and to control cell fate determination in multicellular animals [20, 21]. Dll3 binds to Deltex 1 via its unique N-terminus [22]. Deltex 1 serves as an important signaling transcriptional regulator downstream of Notch receptor [23]. Notch receptor is a critical downstream effector of arteriogenic and angiogenic responses to vascular endothelial growth factor (VEGF) [24]. Our immunohistochemical and immunofluorescence results provide the first evidence that Dll3 is localized uniquely to VECs in kidney, although the precise role of Deltex/Notch signaling in governing endothelial cell behavior remains unclear. In kidney, Dll3, a newly identified ligand responsible for activation of Notch receptor, was uniquely expressed in arterial endothelium, indicating that Dll3 may potentially be a new VEC marker protein and suggesting a potential role of Dll3 in modulating arterial development (arteriogenesis). Further studies are needed to evaluate the roles of Dll3 in kidney VECs and to gain further insight into the critical role of Notch signaling in arteriogenesis and angiogenesis.

Beyond single-protein functional studies in kidney VECs, our study opens the door to understanding the biologic roles of kidney VEC plasma membrane proteins and provides important details about biologic processes, molecular functions, and molecular relationships within the proteome. Moreover, previous proteomic analyses identified approximately 60 proteins in cultured endothelial cells, although few proteins were VEC marker proteins [3, 25]. We believe that our results provide a relatively comprehensive profile of plasma membrane proteins of in vivo kidney VECs and may contribute to a better understanding of the roles and functions of VECs in the kidney.

FatiGO algorithms were used to identify enriched cellular component terms such as apical plasma membrane, basolateral plasma membrane, and membrane fraction. Functions such as binding, signaling, transport, and adhesion are typically associated with plasma membrane proteins. Moreover, VEC-associated functions such as leukocyte adhesion and vesicle-mediated transport were also significantly enriched.

In addition, proteins categorized into phospholipase inhibitor activity and thyroid hormone transmembrane transporter terms were also highly enriched in the VEC plasma membrane proteome. Mining into those two categories, we found that 5 annexin family proteins (ANXA1, ANXA2, ANXA3, ANXA6, and ANXA11) were included in the phospholipase inhibitor activity term. Annexins, as a family of plasma membrane-associated proteins, mediate signaling and binding functions. Gerke et al. [26] reported that members of the annexin family act as receptors for serum proteases on VECs as well as inhibitors of neutrophil migration and blood coagulation. Annexins were also annotated as angiogenesis molecules in the GO annotation. In our results, only solute carrier organic anion transporter family member 1A5 (Slco1a5) was categorized as a thyroid hormone transmembrane transporter. Slco1a5, a member of the organic anion transporter family, is highly expressed in the kidney and moderately abundant in the retina. The transporter is reported to mediate the Na^+^-independent transport of organic anions such as taurocholate and thyroid hormones. Ohtsuki et al. [27] demonstrated Slco1a5 localization in the capillary endothelial cells of brain. These studies have provided basic functional knowledge about VEC functions, and further proteomic analysis of kidney VEC plasma membrane will provide more knowledge about functions and roles in both physiologic and pathologic conditions in the kidney.

## Conclusions

We demonstrated that the CCSN method is a viable, effective technique for directly isolating VEC plasma membrane from the kidney. More than 580 proteins of kidney VEC plasma membrane were identified, and profiling may provide direct insight into the biologic functions of renal VECs in vivo. The technology and results described here may be exploited to better understand the roles of VECs in kidney diseases in the future.

## Electronic supplementary material

Below is the link to the electronic supplementary material.

Supplementary material 1 (DOCX 95 kb)

Supplementary material 2 (DOCX 160 kb)

## Abbreviations

VEC: Vascular endothelial cell
CCSN: Cationic colloidal silica nanoparticles
LC–MS/MS: Liquid chromatography–tandem mass spectrometry
SDS-PAGE: Sodium dodecyl sulfate polyacrylamide gel electrophoresis
KECPMP: Kidney endothelial cell plasma membrane proteins
KLP: Kidney lysate proteins
