# Supplementary material for: Profiling of kidney vascular endothelial cell plasma membrane proteins by liquid chromatography–tandem mass spectrometry
Source: Clin Exp Nephrol. 2012 Dec 11;17(3):327–37. doi: 10.1007/s10157-012-0708-1 (PMC3684716; doi:10.1007/s10157-012-0708-1)
Supplement: Supplementary file 1 — Supplementary material 1 (DOCX 95 kb) [file 10157_2012_708_MOESM1_ESM.docx]

| **Supplementary Table 1** | | | | |
| --- | --- | --- | --- | --- |
| **kidney VEC plasma membrane proteome** | | | | |
| **integral and lipid-anchored** | | | | |
| accession | ns1:prot_desc | prot_  score | prot_  mass | prot_  matches |
| IPI00187747 | Rap1a Ras-related protein Rap-1A | 119 | 21316 | 2 |
| IPI00187967 | Tmem205 transmembrane protein 205 | 86 | 21456 | 4 |
| IPI00188119 | Atp12a Isoform Long of Potassium-transporting ATPase alpha chain 2 | 21 | 114975 | 2 |
| IPI00188509 | Dad1 Dolichyl-diphosphooligosaccharide--protein glycosyltransferase subunit DAD1 | 64 | 12660 | 1 |
| IPI00189503 | Clic5 Chloride intracellular channel protein 5 | 24 | 28299 | 10 |
| IPI00189562 | Slc21a4 Isoform 1 of Solute carrier organic anion transporter family member 1A3 | 80 | 75365 | 9 |
| IPI00189690 | Rab8b Ras-related protein Rab-8B | 52 | 23759 | 3 |
| IPI00190402 | Ugt2b17 UDP-glucuronosyltransferase 2B1 | 111 | 61072 | 11 |
| IPI00191761 | Rab5c Rab5c protein | 54 | 23639 | 1 |
| IPI00191929 | Tmem27 Collectrin | 71 | 25320 | 2 |
| IPI00192216 | Rab33b RAB33B, member of RAS oncogene family | 54 | 26192 | 1 |
| IPI00192310 | Bcam Basal cell adhesion molecule | 120 | 68268 | 2 |
| IPI00193212 | Clec4f C-type lectin domain family 4 member F | 147 | 61523 | 14 |
| IPI00193374 | Slc10a1 Sodium/bile acid cotransporter | 102 | 39725 | 2 |
| IPI00193816 | Abce1 ATP-binding cassette, sub-family E (OABP), member 1 | 47 | 68226 | 5 |
| IPI00195173 | Cd59 CD59 glycoprotein | 47 | 14465 | 1 |
| IPI00196643 | Slc2a2 Solute carrier family 2 (Facilitated glucose transporter), member 2 | 102 | 57432 | 10 |
| IPI00196789 | Rab8a Ras-related protein Rab-8A | 54 | 23824 | 4 |
| IPI00196794 | Rab14 Ras-related protein Rab-14 | 85 | 24140 | 2 |
| IPI00196795 | Rab15 Ras-related protein Rab-15 | 54 | 24610 | 2 |
| IPI00198695 | Itgb3 Integrin beta 3 | 118 | 90066 | 11 |
| IPI00199224 | Rab4b Ras-related protein Rab-4B | 54 | 23899 | 3 |
| IPI00199248 | Rab26 Ras-related protein Rab-26 | 62 | 64915 | 4 |
| IPI00199872 | Gnas Isoform Gnas-1 of Guanine nucleotide-binding protein G(s) subunit alpha isoforms short | 58 | 46091 | 3 |
| IPI00200747 | ECE1 Endothelin-converting enzyme 1 | 96 | 77410 | 14 |
| IPI00201120 | Slc5a2 Low affinity Na-dependent glucose transporter SGLT2 delta e trans | 52 | 66918 | 1 |
| IPI00202570 | Rab2a Ras-related protein Rab-2A | 87 | 23692 | 4 |
| IPI00202689 | Ust5r Integral membrane transport protein UST5r | 153 | 62989 | 13 |
| IPI00203760 | Rab39 RAB39, member RAS oncogene family | 58 | 25304 | 2 |
| IPI00203992 | Pdzk1ip1 PDZK1-interacting protein 1 | 85 | 12349 | 2 |
| IPI00204239 | Gnb3 Guanine nucleotide-binding protein G(I)/G(S)/G(T) subunit beta-3 | 62 | 38125 | 9 |
| IPI00204737 | Rab43 Ras-related protein Rab-43 | 52 | 23443 | 2 |
| IPI00204843 | Gnao1 Isoform Alpha-2 of Guanine nucleotide-binding protein G(o) subunit alpha | 58 | 40568 | 2 |
| IPI00205325 | Lrp2 Low-density lipoprotein receptor-related protein 2 | 266 | 537697 | 28 |
| IPI00205693 | Atp1a2 Sodium/potassium-transporting ATPase subunit alpha-2 | 173 | 113457 | 13 |
| IPI00208111 | Hsd17b2 Estradiol 17-beta-dehydrogenase 2 | 58 | 42567 | 5 |
| IPI00208154 | Cd81 CD81 antigen | 68 | 26555 | 4 |
| IPI00208175 | Tmem33 Transmembrane protein 33 | 55 | 28307 | 3 |
| IPI00208422 | Dpp4 Dipeptidyl peptidase 4 | 297 | 88774 | 35 |
| IPI00208799 | Slco1a5 Solute carrier organic anion transporter family member 1A5 | 57 | 76066 | 3 |
| IPI00209150 | Rab3c Ras-related protein Rab-3C | 54 | 26084 | 3 |
| IPI00209291 | Esyt1 Extended synaptotagmin-1 | 50 | 121369 | 5 |
| IPI00210008 | Tmed2 Transmembrane emp24 domain-containing protein 2 | 84 | 22832 | 3 |
| IPI00210346 | Gnal Guanine nucleotide-binding protein G(olf) subunit alpha | 58 | 44734 | 3 |
| IPI00210381 | Rab11b Ras-related protein Rab-11B | 111 | 24588 | 3 |
| IPI00210733 | Rab5a Small GTP-binding protein rab5 | 68 | 23828 | 4 |
| IPI00210884 | Dnaja1 DnaJ homolog subfamily A member 1 | 418 | 45581 | 29 |
| IPI00211616 | Slc3a2 4F2 cell-surface antigen heavy chain | 46 | 58150 | 1 |
| IPI00211648 | Slc3a1 Neutral and basic amino acid transport protein rBAT | 58 | 78913 | 2 |
| IPI00211732 | Adam10 similar to ADAM 10 precursor | 45 | 73593 | 2 |
| IPI00212655 | Gnb1 Guanine nucleotide-binding protein G(I)/G(S)/G(T) subunit beta-1 | 72 | 38151 | 12 |
| IPI00212658 | Gnb2 Guanine nucleotide-binding protein G(I)/G(S)/G(T) subunit beta-2 | 30 | 37331 | 6 |
| IPI00212796 | Ran GTP-binding nuclear protein Ran | 107 | 24579 | 10 |
| IPI00214004 | Slco1a6 Solute carrier organic anion transporter family member 1A6 | 57 | 76169 | 3 |
| IPI00214031 | Slco1a4 Solute carrier organic anion transporter family member 1A4 | 46 | 74857 | 11 |
| IPI00214674 | Slco1a1 Solute carrier organic anion transporter family member 1A1 | 49 | 75782 | 6 |
| IPI00214787 | Slc16a1 Monocarboxylate transporter 1 | 178 | 53831 | 8 |
| IPI00215390 | Slco1b2 Isoform 1 of Solute carrier organic anion transporter family member 1B2 | 86 | 73859 | 10 |
| IPI00215564 | Rab7a Ras-related protein Rab-7a | 60 | 23774 | 1 |
| IPI00230862 | Anpep Aminopeptidase N | 88 | 109779 | 5 |
| IPI00230866 | Gna12 Guanine nucleotide-binding protein subunit alpha-12 | 49 | 44208 | 7 |
| IPI00231001 | Gnat3 Guanine nucleotide-binding protein G(t) subunit alpha-3 | 47 | 40781 | 8 |
| IPI00231181 | Slc21a4 Isoform 7 of Solute carrier organic anion transporter family member 1A3 | 44 | 64358 | 5 |
| IPI00231451 | Atp1a3 Sodium/potassium-transporting ATPase subunit alpha-3 | 173 | 113045 | 11 |
| IPI00231601 | Ptprc Isoform 4 of Receptor-type tyrosine-protein phosphatase C | 75 | 136173 | 5 |
| IPI00231629 | Flot1 Isoform 2 of Flotillin-1 | 77 | 40144 | 3 |
| IPI00010737 | Thbd Thrombomodulin | 56 | 37339 | 4 |
| IPI00231659 | Tmed10 Transmembrane emp24 domain-containing protein 10 | 94 | 25013 | 4 |
| IPI00231726 | Gnai3 Guanine nucleotide-binding protein G(k) subunit alpha | 72 | 41066 | 8 |
| IPI00231733 | Gnai1 Guanine nucleotide-binding protein G(i) subunit alpha-1 | 58 | 40889 | 3 |
| IPI00231738 | Asgr1 Asialoglycoprotein receptor 1 | 48 | 33398 | 3 |
| IPI00231925 | Gnai2 Guanine nucleotide-binding protein G(i) subunit alpha-2 | 283 | 41043 | 13 |
| IPI00324309 | Cybb Endothelial type gp91-phox | 52 | 66163 | 3 |
| IPI00324585 | Itga1 Integrin alpha-1 | 135 | 21323 | 5 |
| IPI00325762 | Rab3a Ras-related protein Rab-3A | 58 | 25182 | 2 |
| IPI00325860 | Podxl Podocalyxin | 22 | 51658 | 4 |
| IPI00326305 | Atp1a1 Sodium/potassium-transporting ATPase subunit alpha-1 | 613 | 114293 | 28 |
| IPI00327202 | Aqp1 Aquaporin-1 | 116 | 29066 | 4 |
| IPI00327398 | Enpep Isoform 1 of Glutamyl aminopeptidase | 101 | 108440 | 8 |
| IPI00327697 | Dpep1 Dipeptidase 1 | 70 | 45949 | 1 |
| IPI00332009 | RT1-CE1 RT1 class I, locus CE1 | 52 | 42534 | 4 |
| IPI00339124 | Atp1b1 Sodium/potassium-transporting ATPase subunit beta-1 | 51 | 35578 | 6 |
| IPI00358313 | Plp2 Proteolipid protein 2 | 66 | 16831 | 1 |
| IPI00360541 | Itgb2 integrin beta 2 | 113 | 87955 | 9 |
| IPI00361512 | Abca8a similar to ATP-binding cassette, sub-family A (ABC1), member 8a | 67 | 192706 | 42 |
| IPI00362251 | RT1-N2 RT1 class Ib, locus N2 | 50 | 43719 | 3 |
| IPI00363265 | Hspa9 Stress-70 protein, mitochondrial | 58 | 74097 | 7 |
| IPI00364707 | Tmed9 Transmembrane emp24 protein transport domain containing 9 | 59 | 27182 | 2 |
| IPI00365705 | Atp4a potassium-transporting ATPase alpha chain 1 | 70 | 115745 | 3 |
| IPI00365888 | Rab2b RAB2B, member RAS oncogene family | 25 | 24085 | 11 |
| IPI00366221 | Rab21 Ras-related protein Rab-21 | 63 | 24547 | 3 |
| IPI00368180 | Myof similar to myoferlin isoform b | 133 | 236511 | 5 |
| IPI00368308 | Gnat2 guanine nucleotide binding protein, alpha transducing 2 | 342 | 69213 | 15 |
| IPI00369995 | Lrp1 low density lipoprotein receptor-related protein 1 | 192 | 523489 | 20 |
| IPI00370158 | Rac2 Ras-related C3 botulinum toxin substrate 2 | 70 | 21826 | 2 |
| IPI00371187 | Rab35 Ras-related protein Rab-35 | 52 | 23296 | 2 |
| IPI00371269 | Rab5b RAB5B, member RAS oncogene family (Predicted), isoform CRA_c | 63 | 23888 | 2 |
| IPI00372689 | Itga3 117 kDa protein | 61 | 118024 | 1 |
| IPI00372732 | Emcn Endomucin | 56 | 26614 | 2 |
| IPI00372839 | Col6a2 collagen, type VI, alpha 2 | 46 | 110732 | 13 |
| IPI00372952 | Icam2 Intercellular adhesion molecule 2 | 71 | 31641 | 3 |
| IPI00373076 | Atp6v1a ATPase, H+ transporting, lysosomal V1 subunit A | 86 | 68564 | 2 |
| IPI00390785 | Gnal similar to guanine nucleotide binding protein (G protein), alpha activating activity polypeptide, olfactory type isoform 1 isoform 1 | 49 | 52266 | 5 |
| IPI00400615 | Atp6v1e1 V-type proton ATPase subunit E 1 | 101 | 26169 | 6 |
| IPI00400616 | Vwa5a von Willebrand factor A domain-containing protein 5A | 49 | 92280 | 6 |
| IPI00411191 | RT1-Cl MHC class Ib antigen | 62 | 41469 | 6 |
| IPI00411230 | Gstm2 Glutathione S-transferase Mu 2 | 86 | 25857 | 12 |
| IPI00421897 | Rab1 Ras-related protein Rab-1A | 35 | 22677 | 18 |
| IPI00421966 | RT1-T24-4 RT1 class I, locus T24, gene 4 | 52 | 42891 | 2 |
| IPI00421980 | RT1-CE16 RCG41814 | 52 | 41474 | 6 |
| IPI00421982 | RT1-CE13 RT1 class I, CE13 | 52 | 41339 | 4 |
| IPI00421983 | RT1-CE12 RT1 class I, CE12 | 69 | 41877 | 3 |
| IPI00421985 | RT1-CE10 RT1-CE10 protein | 82 | 41710 | 5 |
| IPI00422053 | Gna13 Guanine nucleotide binding protein alpha 13 | 60 | 44326 | 5 |
| IPI00422067 | Gnb4 Guanine nucleotide-binding protein subunit beta-4 | 72 | 38080 | 6 |
| IPI00422092 | Rac1 Ras-related C3 botulinum toxin substrate 1 | 52 | 21835 | 3 |
| IPI00454534 | Eng endoglin | 48 | 70812 | 2 |
| IPI00464469 | Scarb1 Scavenger receptor class B type 2 | 56 | 56705 | 7 |
| IPI00551631 | RT1-A3 MHC class I RT1.Ac heavy chain | 52 | 41505 | 6 |
| IPI00551661 | RT1-CE5 MHC class Ib antigen (Fragment) | 53 | 30624 | 2 |
| IPI00551718 | RT1-A2 RT1 class Ia, locus A2 | 263 | 41710 | 9 |
| IPI00551731 | RT1-L3 MHC class Ib antigen (Fragment) | 52 | 38296 | 4 |
| IPI00551732 | Dnaja2 DnaJ (Hsp40) homolog, subfamily A, member 2 | 93 | 46344 | 9 |
| IPI00555185 | Rab10 RAB10, member RAS oncogene family | 111 | 22755 | 7 |
| IPI00557493 | RT1-CE7 MHC class I protein | 52 | 41359 | 4 |
| IPI00558853 | RT1-CE4 MHC class I protein | 51 | 42398 | 5 |
| IPI00558908 | Slc9a3r2 | 142 | 35481 | 7 |
| IPI00560847 | Dab2 | 26 | 80547 | 3 |
| IPI00566654 | Rab4a 24 kDa protein | 54 | 24232 | 5 |
| IPI00566814 | Cd200 OX-2 membrane glycoprotein | 49 | 31467 | 1 |
| IPI00655278 | RT1-CE14 RT1 class I, CE14 | 69 | 39534 | 5 |
| IPI00762951 | Abca6 similar to ATP-binding cassette, sub-family A (ABC1), member 6 | 81 | 185449 | 9 |
| IPI00763480 | Rab6a similar to RAB6A, member RAS oncogene family | 55 | 57323 | 7 |
| IPI00766812 | RT1-CE3 similar to RT1 class I, CE3 | 52 | 29663 | 4 |
| IPI00767110 | Integrin alpha-IIb precursor | 100 | 114075 | 6 |
| IPI00767698 | Rab6b RAB6B, member RAS oncogene family | 54 | 19979 | 3 |
| IPI00778978 | Slc4a1 Slc4a1 protein | 60 | 94580 | 4 |
| IPI00781739 | Rab30 23 kDa protein | 52 | 23386 | 2 |
| IPI00855220 | RT1-EC2 MHC class Ia protein | 252 | 42000 | 7 |
| IPI00876621 | Rab12 ras-related protein Rab-12 | 54 | 27596 | 2 |
| IPI00876636 | Nckap1 Nck-associated protein 1 | 158 | 130093 | 28 |
| IPI00949667 | Lims1 39 kDa protein | 72 | 40430 | 4 |
| IPI00950239 | Bsg | 251 | 29980 | 15 |
| **Cytoskeletal and/or junctional proteins** | |  |  |  |
| IPI00372040 | Arpc4 protein | 78 | 19768 | 4 |
| IPI00189813 | Acta1 Actin, alpha skeletal muscle | 718 | 42366 | 83 |
| IPI00197129 | Acta2 Actin, aortic smooth muscle | 679 | 42381 | 79 |
| IPI00200455 | Actg2 Actin, gamma-enteric smooth muscle | 89 | 42249 | 17 |
| IPI00454431 | Actn1 Brain-specific alpha actinin 1 isoform | 45 | 106032 | 5 |
| IPI00363022 | Actn2 actinin alpha 2 | 49 | 104339 | 9 |
| IPI00200773 | Actn3 Alpha-actinin-3 | 56 | 103575 | 3 |
| IPI00213463 | Actn4 Alpha-actinin-4 | 155 | 105306 | 16 |
| IPI00362072 | Actr2 Actin-related protein 2 | 70 | 44990 | 8 |
| IPI00768299 | Arpc3 Actin related protein 2/3 complex, subunit 3 (Predicted), isoform CRA_b | 48 | 20750 | 4 |
| IPI00213408 | Arpc5l Isoform 2 of Actin-related protein 2/3 complex subunit 5-like protein | 51 | 11228 | 2 |
| IPI00337168 | Cct4 T-complex protein 1 subunit delta | 90 | 58576 | 7 |
| IPI00193983 | Cltc Clathrin heavy chain 1 | 124 | 193187 | 13 |
| IPI00358406 | Ctnna1 Catenin (Cadherin-associated protein), alpha 1, isoform CRA_b | 659 | 100858 | 40 |
| IPI00870129 | Ctnna2 catenin, alpha 2 | 153 | 106018 | 16 |
| IPI00325912 | Ctnnb1 Catenin beta-1 | 231 | 86027 | 18 |
| IPI00421517 | Des Desmin | 48 | 53481 | 9 |
| IPI00870112 | Dpysl2 Dihydropyrimidinase-related protein 2 | 51 | 62638 | 1 |
| IPI00763877 | Dtx3l deltex 3-like | 66 | 84335 | 3 |
| IPI00470254 | Ezr Ezrin | 341 | 69462 | 37 |
| IPI00362106 | Fermt2 RCG61183, isoform CRA_b | 140 | 78453 | 15 |
| IPI00951791 | Flna Filamin, alpha (Predicted), isoform CRA_a | 87 | 283057 | 9 |
| IPI00208205 | Hspa8 Heat shock cognate 71 kDa protein | 1162 | 71055 | 53 |
| IPI00194728 | Ilk Integrin-linked protein kinase | 226 | 51853 | 25 |
| IPI00950128 | Iqgap1 Iqgap1 protein | 419 | 133197 | 30 |
| IPI00421429 | Jup Junction plakoglobin | 53 | 82490 | 9 |
| IPI00364904 | Kif5b Kinesin-1 heavy chain | 149 | 110204 | 13 |
| IPI00193402 | Kif5c kinesin heavy chain isoform 5C | 79 | 109685 | 6 |
| IPI00421625 | Mrlc2 Myosin regulatory light chain 12B | 336 | 19883 | 17 |
| IPI00212314 | Msn Moesin | 265 | 67868 | 35 |
| IPI00200640 | Mucdhl Isoform 1 of Mucin and cadherin-like protein | 68 | 91262 | 1 |
| IPI00339007 | Myadm Myeloid-associated differentiation marker | 61 | 35866 | 1 |
| IPI00949586 | Myh10 Myosin-10 | 47 | 229793 | 12 |
| IPI00767676 | Myh11 myosin-11 | 144 | 228337 | 14 |
| IPI00367479 | Myh14 myosin, heavy chain 14 | 348 | 229630 | 28 |
| IPI00209113 | Myh9 Myosin-9 | 2905 | 227566 | 199 |
| IPI00231788 | Myl3 Myosin light chain 3 | 62 | 22256 | 9 |
| IPI00817070 | Myl6 myosin, light chain 6, alkali, smooth muscle and non-muscle | 111 | 16961 | 55 |
| IPI00870820 | Myl6b;Myl6 myosin, light chain 6B, alkali, smooth muscle and non-muscle | 94 | 12634 | 9 |
| IPI00365944 | Myl6l Myosin light polypeptide 6 | 94 | 17135 | 13 |
| IPI00858359 | Myl9 Myosin, light polypeptide 9, regulatory | 56 | 19898 | 4 |
| IPI00780133 | Mylc2b Myosin regulatory light chain-B | 27 | 19608 | 12 |
| IPI00391496 | Myo1b Isoform B of Myosin-Ib | 103 | 129544 | 24 |
| IPI00393867 | Myo1c Myosin-Ic | 132 | 120535 | 23 |
| IPI00207989 | Myo1d Myosin-Id | 135 | 116820 | 23 |
| IPI00207988 | Myo1e Myosin-Ie | 47 | 127374 | 7 |
| IPI00188524 | Nefh Neurofilament heavy polypeptide | 11 | 115378 | 1 |
| IPI00231302 | Nefl Neurofilament light polypeptide | 11 | 61335 | 1 |
| IPI00779968 | Nf2 25 kDa protein | 114 | 25039 | 8 |
| IPI00231136 | Nid1 similar to Nidogen-1 precursor | 130 | 139746 | 3 |
| IPI00372786 | Nid2 Isoform 1 of Nidogen-2 | 65 | 155674 | 3 |
| IPI00194959 | Picalm Picalm protein | 111 | 70202 | 6 |
| IPI00230793 | Plec1 Isoform 3 of Plectin-1 | 202 | 518765 | 64 |
| IPI00557051 | Plec1 Plectin 8 | 202 | 518992 | 62 |
| IPI00817060 | Podn 59 kDa protein | 46 | 59376 | 11 |
| IPI00326582 | Prph peripherin | 48 | 54063 | 9 |
| IPI00564409 | RGD1309537 Myosin regulatory light chain RLC-A | 73 | 19940 | 5 |
| IPI00200523 | Slc9a3r2 Na(+)/H(+) exchange regulatory cofactor NHE-RF2 | 60 | 37688 | 2 |
| IPI00209258 | Spna2 285 kDa protein | 149 | 285663 | 29 |
| IPI00776619 | Spna2 Spectrin alpha chain, brain | 1480 | 285261 | 116 |
| IPI00372717 | Spta1 Erythroid spectrin alpha | 49 | 277705 | 9 |
| IPI00555287 | Sptbn1 Non-erythroid spectrin beta | 961 | 274692 | 98 |
| IPI00327662 | Sptbn2 Spectrin beta chain, brain 2 | 146 | 272151 | 30 |
| IPI00362014 | Tln1 talin | 982 | 271671 | 63 |
| IPI00197888 | Tpm1 Isoform 1 of Tropomyosin alpha-1 chain | 38 | 32680 | 12 |
| IPI00230775 | Tpm2 Isoform 1 of Tropomyosin beta chain | 79 | 32931 | 5 |
| IPI00372259 | Tpm3 Isoform 1 of Tropomyosin alpha-3 chain | 38 | 29006 | 14 |
| IPI00214905 | Tpm4 Tropomyosin alpha-4 chain | 148 | 28549 | 9 |
| IPI00189795 | Tuba1a Tubulin alpha-1A chain | 141 | 50788 | 10 |
| IPI00339167 | Tuba1b Tubulin alpha-1B chain | 155 | 50932 | 8 |
| IPI00364046 | Tuba1c Tubulin alpha-1C chain | 155 | 50590 | 9 |
| IPI00471523 | Tuba3a;Tuba3b Tubulin alpha-3 chain | 125 | 50612 | 9 |
| IPI00362927 | Tuba4a Tubulin alpha-4A chain | 98 | 50634 | 7 |
| IPI00464587 | Tuba8 Tubulin alpha-8 chain | 98 | 50690 | 9 |
| IPI00870141 | Tubal3 tubulin, alpha-like 3 | 12 | 42833 | 2 |
| IPI00475639 | Tubb2a Tubulin beta-2A chain | 57 | 50274 | 2 |
| IPI00655259 | Tubb2b Tubulin beta-2B chain | 142 | 49953 | 35 |
| IPI00400573 | Tubb2c Tubulin beta-2C chain | 602 | 50225 | 33 |
| IPI00362160 | Tubb3 Tubulin beta-3 chain | 355 | 50842 | 21 |
| IPI00765366 | Tubb4 tubulin, beta 4 | 335 | 50010 | 28 |
| IPI00829443 | Tubb5 Isoform 2 of Tubulin beta-5 chain | 85 | 24271 | 39 |
| IPI00195673 | Tubb6 Tubulin, beta 6 | 291 | 50483 | 18 |
| IPI00950163 | Tubgcp3 tubulin, gamma complex associated protein 3 | 52 | 68369 | 2 |
| IPI00362757 | Vil1 Vil1 protein | 66 | 93115 | 7 |
| IPI00230941 | Vim Vimentin | 48 | 53757 | 6 |
| IPI00189723 | Tropomodulin 1 |  |  |  |
| **Peripherally associated on inside** | |  |  |  |
| IPI00188884 | Abp10 similar to CG5514-PB, isoform B | 46 | 350481 | 91 |
| IPI00231615 | Anxa1 Annexin A1 | 125 | 39147 | 4 |
| IPI00364621 | Anxa11 Annexin A11 | 204 | 54468 | 11 |
| IPI00829462 | Anxa2 Isoform Long of Annexin A2 | 335 | 49172 | 26 |
| IPI00207390 | Anxa3 Annexin A3 | 56 | 36569 | 6 |
| IPI00471889 | Anxa5 Annexin A5 | 81 | 35779 | 1 |
| IPI00421888 | Anxa6 Annexin A6 | 442 | 76106 | 36 |
| IPI00201713 | Ap1b1 Isoform B of AP-1 complex subunit beta-1 | 70 | 104662 | 3 |
| IPI00567919 | Ap2a1 105 kDa protein | 263 | 106468 | 19 |
| IPI00471901 | Ap2a2 Adaptor-related protein complex 2, alpha 2 subunit | 503 | 105020 | 34 |
| IPI00231502 | Ap2b1 Isoform 2 of AP-2 complex subunit beta | 204 | 106537 | 24 |
| IPI00360117 | Ap3b1 122 kDa protein | 45 | 122053 | 6 |
| IPI00211127 | Ass1 Argininosuccinate synthase | 117 | 46752 | 14 |
| IPI00231955 | Calm1;Calm2;Calm3 Calmodulin | 68 | 16827 | 3 |
| IPI00215463 | Calml3 Calmodulin-like protein 3 | 72 | 16849 | 1 |
| IPI00285606 | Cdc42 Isoform 1 of Cell division control protein 42 homolog | 64 | 21587 | 2 |
| IPI00471800 | Cdh16 Cadherin 16 | 54 | 90519 | 2 |
| IPI00362534 | Ddx3x DEAD (Asp-Glu-Ala-Asp) box polypeptide 3, X-linked | 430 | 73499 | 31 |
| IPI00952120 | Dnm2 Isoform 4 of Dynamin-2 | 101 | 97971 | 19 |
| IPI00766164 | Dst 300 kDa protein | 44 | 302218 | 16 |
| IPI00212694 | Egfr Epidermal growth factor receptor | 47 | 138225 | 7 |
| IPI00360340 | Ehd1 EH domain-containing protein 1 | 69 | 60622 | 5 |
| IPI00200258 | Ehd3 EH domain-containing protein 3 | 173 | 60810 | 22 |
| IPI00200271 | Ehd4 Pincher | 69 | 61657 | 5 |
| IPI00324275 | Exoc2 Exocyst complex component 2 | 95 | 104992 | 7 |
| IPI00209037 | Fkbp2 FK506 binding protein 2 | 67 | 15549 | 2 |
| IPI00214149 | Grb14 Growth factor receptor-bound protein 14 | 71 | 61181 | 3 |
| IPI00211507 | Hpd 4-hydroxyphenylpyruvate dioxygenase | 54 | 45312 | 9 |
| IPI00194792 | Lancl1 LanC-like protein 1 | 77 | 45952 | 5 |
| IPI00568559 | Lims1 LIM and senescent cell antigen-like domains 1 isoform D | 84 | 43463 | 6 |
| IPI00559178 | Mst4 similar to serine/threonine protein kinase MASK | 56 | 74414 | 4 |
| IPI00421389 | Ndrg1 Protein NDRG1 | 74 | 43383 | 1 |
| IPI00211048 | Nf2 merlin | 114 | 69445 | 7 |
| IPI00198887 | P4hb Protein disulfide-isomerase | 376 | 57315 | 30 |
| IPI00213498 | Pacsin2 Isoform 1 of Protein kinase C and casein kinase substrate in neurons 2 protein | 96 | 56285 | 9 |
| IPI00950520 | Pacsin3 Protein kinase C and casein kinase substrate in neurons 3 | 58 | 48953 | 6 |
| IPI00365929 | Pdia6 protein disulfide-isomerase A6 | 369 | 49129 | 14 |
| IPI00211756 | Phb Prohibitin | 227 | 29859 | 7 |
| IPI00209033 | Plcb3 Phospholipase C beta 3 | 109 | 140437 | 7 |
| IPI00208265 | Ppp1ca Serine/threonine-protein phosphatase PP1-alpha catalytic subunit | 53 | 38229 | 4 |
| IPI00203390 | Ppp1cb Serine/threonine-protein phosphatase PP1-beta catalytic subunit | 73 | 37961 | 5 |
| IPI00203358 | Ppp1cc Isoform Gamma-1 of Serine/threonine-protein phosphatase PP1-gamma catalytic subunit | 20 | 36984 | 8 |
| IPI00388209 | Prkcsh Protein kinase C substrate 80K-H (Predicted), isoform CRA_b | 94 | 60150 | 3 |
| IPI00369635 | Rdx Radixin | 1020 | 68672 | 88 |
| IPI00201699 | Rhoa Transforming protein RhoA | 24 | 21782 | 11 |
| IPI00191114 | Rhoc Ras homolog gene family, member C | 12 | 22036 | 5 |
| IPI00364932 | Rsu1 Ras suppressor protein 1 | 122 | 31442 | 18 |
| IPI00214447 | RT1-N3 RT1 class Ib, locus N3 | 52 | 45562 | 2 |
| IPI00869818 | Sec23a SEC23A | 305 | 87019 | 16 |
| IPI00199716 | Sec23b 86 kDa protein | 69 | 87212 | 5 |
| IPI00777116 | Sec3l1 84 kDa protein | 54 | 84689 | 5 |
| IPI00364983 | Sec61b Sec61 beta subunit | 47 | 10039 | 2 |
| IPI00363930 | Sept11 Isoform 1 of Septin-11 | 107 | 50005 | 9 |
| IPI00208304 | Sept2 Septin-2 | 201 | 41737 | 13 |
| IPI00766474 | Sept6 similar to septin 6 | 48 | 60181 | 7 |
| IPI00884567 | Sept7 septin-7 isoform b | 144 | 50989 | 10 |
| IPI00211336 | Sh3bgrl3 SH3 domain binding glutamic acid-rich protein-like 3 (Predicted), isoform CRA_b | 58 | 10527 | 2 |
| IPI00200898 | Slc9a3r1 Na(+)/H(+) exchange regulatory cofactor NHE-RF1 | 89 | 39149 | 8 |
| IPI00198369 | Snx1 Sorting nexin 1 | 67 | 59122 | 5 |
| IPI00870489 | Snx2 sorting nexin 2 | 83 | 58609 | 9 |
| IPI00365613 | Snx6 Sorting nexin 6 | 56 | 46893 | 1 |
| IPI00763992 | Srp72 similar to Signal recognition particle 72 kDa protein | 49 | 74499 | 11 |
| IPI00390595 | Stk25 Serine/threonine kinase 25 (STE20 homolog, yeast), isoform CRA_a | 56 | 48356 | 3 |
| IPI00949426 | Stoml2 33 kDa protein | 56 | 33325 | 1 |
| IPI00390422 | Tbckl TBC domain-containing protein kinase-like | 47 | 98006 | 5 |
| IPI00198550 | Uba52 ubiquitin A-52 residue ribosomal protein fusion product 1 | 42 | 14728 | 29 |
| IPI00882520 | Ubb Polyubiquitin (Fragment) | 42 | 11241 | 38 |
| IPI00476033 | Ubc Ubc protein | 42 | 48338 | 8 |
| IPI00869568 | Vps35 maternal embryonic message 3 | 59 | 92467 | 6 |
| IPI00230835 | Ywhag 14-3-3 protein gamma | 52 | 28456 | 9 |
| **Out bound-secreted/blood** | |  |  |  |
| IPI00190428 | Tinagl1 Tubulointerstitial nephritis antigen-like | 90 | 54155 | 2 |
| IPI00190577 | Lama5 similar to Laminin alpha-5 chain precursor | 73 | 416613 | 8 |
| IPI00193049 | Sult1a1 Sulfotransferase 1A1 | 130 | 34169 | 8 |
| IPI00193258 | Pah Phenylalanine-4-hydroxylase | 69 | 52303 | 7 |
| IPI00193277 | Hsd17b11 Estradiol 17-beta-dehydrogenase 11 | 64 | 33316 | 2 |
| IPI00195160 | Psap Sulfated glycoprotein 1 | 51 | 62908 | 1 |
| IPI00197711 | Ldha L-lactate dehydrogenase A chain | 331 | 36712 | 20 |
| IPI00200344 | Scpep1 Retinoid-inducible serine carboxypeptidase | 73 | 51427 | 1 |
| IPI00201262 | Mug1;LOC297568 Alpha-1-inhibitor 3 | 133 | 165038 | 17 |
| IPI00204359 | B2m Beta-2-microglobulin | 116 | 13825 | 5 |
| IPI00205036 | LOC360504 hemoglobin alpha 2 chain | 49 | 15446 | 6 |
| IPI00205561 | Acox2 Peroxisomal acyl-coenzyme A oxidase 2 | 230 | 77548 | 16 |
| IPI00207146 | MGC72973 Zero beta-1 globin | 267 | 16069 | 22 |
| IPI00211510 | Acox1 Isoform 1 of Peroxisomal acyl-coenzyme A oxidase 1 | 180 | 75030 | 13 |
| IPI00212478 | Hbe1 RCG39817, isoform CRA_a | 49 | 16151 | 23 |
| IPI00212868 | Lamb2 Laminin subunit beta-2 | 199 | 203474 | 7 |
| IPI00230897 | Hbb Hemoglobin subunit beta-1 | 1184 | 16083 | 72 |
| IPI00231982 | Fn1 Isoform 2 of Fibronectin | 61 | 266275 | 9 |
| IPI00287835 | Hba-a2 Hemoglobin subunit alpha-1/2 | 771 | 15490 | 59 |
| IPI00324380 | Ttr Transthyretin | 56 | 15824 | 3 |
| IPI00325484 | Lgals9 Isoform Short of Galectin-9 | 75 | 36692 | 4 |
| IPI00339123 | Acox3 Peroxisomal acyl-coenzyme A oxidase 3 | 56 | 79080 | 4 |
| IPI00371043 | Armet Mesencephalic astrocyte-derived neurotrophic factor | 55 | 20831 | 1 |
| IPI00421711 | Atp5l ATP synthase, H+ transporting, subunit G | 164 | 11453 | 5 |
| IPI00464815 | Eno1 Alpha-enolase | 271 | 47440 | 9 |
| IPI00475946 | Tf Isoform 2 of Serotransferrin | 94 | 55971 | 17 |
| IPI00476458 | Gpx3 Glutathione peroxidase 3 | 215 | 25831 | 5 |
| IPI00476749 | Mug2 Murinoglobulin-2 | 94 | 163035 | 18 |
| IPI00555161 | Apob Apolipoprotein B-100 | 47 | 537740 | 18 |
| IPI00565677 | Col6a3 288 kDa protein | 49 | 289437 | 11 |
| IPI00951327 | Col18a1 183 kDa protein | 68 | 183851 | 6 |
| IPI00201596 | Mup4 Alpha-2u globulin | 94 | 163035 | 18 |
| IPI00214419 | Gpx6 Glutathione peroxidase 6 | 14 | 24961 | 7 |
| IPI00421714 | Lyz2 Lysozyme C | 75 | 5299 | 6 |
| IPI00363849 | Lamc1 laminin, gamma 1 | 106 | 182975 | 7 |
| IPI00959364 | Vwf Von Willebrand factor | 55 | 87480 | 7 |
| **Endoplasmic reticulum proteins** | |  |  |  |
| IPI00195614 | Rdh2 Retinol dehydrogenase 2 | 240 | 35973 | 16 |
| IPI00199636 | Canx Calnexin | 187 | 67612 | 17 |
| IPI00204365 | Rpn1 Ribophorin1 | 189 | 68473 | 11 |
| IPI00204703 | Serpinh1 Serpin H1 | 52 | 46602 | 1 |
| IPI00204774 | Cesl1 Liver carboxylesterase B-1 | 65 | 62968 | 15 |
| IPI00205417 | Slc27a2 Very long-chain acyl-CoA synthetase | 186 | 71447 | 12 |
| IPI00206948 | Retsat All-trans-retinol 13,14-reductase | 46 | 68001 | 4 |
| IPI00208714 | Pip4k2b Phosphatidylinositol-5-phosphate 4-kinase type-2 beta | 50 | 47633 | 2 |
| IPI00365607 | Tmem195 Transmembrane protein 195 | 49 | 52064 | 1 |
| IPI00369774 | Mettl7b Methyltransferase-like protein 7B | 55 | 28342 | 4 |
| IPI00372377 | Fmo6 similar to Putative dimethylaniline monooxygenase [N-oxide-forming] 6 | 98 | 60761 | 2 |
| IPI00480820 | Pgrmc1 Membrane-associated progesterone receptor component 1 | 89 | 21699 | 6 |
| IPI00555268 | Hsd3b6 3 beta-hydroxysteroid dehydrogenase/Delta 5-->4-isomerase type 4 | 75 | 42273 | 8 |
| IPI00947957 | Rpn2 67 kDa protein | 134 | 67196 | 13 |
| IPI00949107 | Srprb Cc1-8 | 119 | 109510 | 22 |
| **Golgi** | |  |  |  |
| IPI00213677 | Arf2 ADP-ribosylation factor 2 | 71 | 20790 | 3 |
| IPI00231674 | Arf3 ADP-ribosylation factor 3 | 71 | 20645 | 4 |
| IPI00231965 | Arf5 ADP-ribosylation factor 5 | 63 | 20631 | 4 |
| IPI00231966 | Arf4 ADP-ribosylation factor 4 | 55 | 20831 | 1 |
| **Ribosomal proteins** | |  |  |  |
| IPI00188058 | Rps18 40S ribosomal protein S18 | 60 | 17708 | 3 |
| IPI00190240 | Rps27a Ribosomal protein S27a | 64 | 18282 | 2 |
| IPI00201500 | Rps14 40S ribosomal protein S14 | 47 | 16420 | 1 |
| IPI00203214 | Eef2 Elongation factor 2 | 440 | 96192 | 50 |
| IPI00203523 | Rpl23a 60S ribosomal protein L23a | 63 | 17684 | 1 |
| IPI00211216 | Eif5a Eukaryotic translation initiation factor 5A-1 | 49 | 17049 | 9 |
| IPI00212776 | Rps3 40S ribosomal protein S3 | 62 | 26828 | 5 |
| IPI00214654 | Rps24 Isoform 1 of 40S ribosomal protein S24 | 53 | 15413 | 2 |
| IPI00215184 | Rps25 40S ribosomal protein S25 | 57 | 18028 | 3 |
| IPI00231473 | Cyp2d2 Cytochrome P450 2D26 | 447 | 56876 | 32 |
| **Nuclues** | |  |  |  |
| IPI00191997 | Eefsec Eefsec protein | 297 | 64303 | 22 |
| IPI00196645 | Prkag1 5'-AMP-activated protein kinase subunit gamma-1 | 79 | 37534 | 4 |
| IPI00201060 | Lmna Lamin-A | 57 | 74564 | 5 |
| IPI00203730 | Rasl2-9 GTP-binding nuclear protein Ran, testis-specific isoform | 68 | 24607 | 8 |
| IPI00204499 | Eif2s3x Eukaryotic translation initiation factor 2 subunit 3 | 294 | 51617 | 24 |
| IPI00205466 | Cand1 Cullin-associated NEDD8-dissociated protein 1 | 55 | 137985 | 8 |
| IPI00210090 | Hnrnpu SP120 | 67 | 88492 | 6 |
| IPI00555214 | Hmgb1 High mobility group protein B1 | 78 | 25049 | 4 |
| IPI00555314 | Ddx1 ATP-dependent RNA helicase DDX1 | 67 | 83414 | 10 |
| IPI00566054 | Pnn 24 kDa protein | 47 | 83572 | 6 |
| IPI00763247 | Samhd1 similar to SAM domain and HD domain-containing protein 1 | 96 | 72504 | 6 |
| IPI00948134 | Luzp1 119 kDa protein | 49 | 119779 | 8 |
| **Mitochondrial proteins** | |  |  |  |
| IPI00188924 | Uqcrc2 Cytochrome b-c1 complex subunit 2, mitochondrial | 73 | 48423 | 4 |
| IPI00188989 | Acsl1 Long-chain-fatty-acid--CoA ligase 1 | 61 | 79155 | 9 |
| IPI00214373 | Aadat Kynurenine/alpha-aminoadipate aminotransferase, mitochondrial | 62 | 48096 | 2 |
| IPI00201413 | Acaa2 3-ketoacyl-CoA thiolase, mitochondrial | 361 | 42244 | 22 |
| IPI00557975 | Acad11 Acyl-CoA dehydrogenase family member 11 | 109 | 88057 | 10 |
| IPI00421539 | Aco2 Aconitate hydratase, mitochondrial | 64 | 86121 | 2 |
| IPI00204118 | Aifm1 Apoptosis-inducing factor 1, mitochondrial | 71 | 66966 | 2 |
| IPI00196725 | Aldh1l1 10-formyltetrahydrofolate dehydrogenase | 74 | 99976 | 21 |
| IPI00197770 | Aldh2 Aldehyde dehydrogenase, mitochondrial | 350 | 56966 | 10 |
| IPI00198620 | Atp5d ATP synthase subunit delta, mitochondrial | 54 | 17584 | 2 |
| IPI00390086 | Atp5j2 similar to ATP synthase, H+ transporting, mitochondrial F0 complex, subunit f, isoform 2 | 49 | 10503 | 9 |
| IPI00194222 | Cox4i1 Cytochrome c oxidase subunit 4 isoform 1, mitochondrial | 49 | 19559 | 3 |
| IPI00231611 | Fh1 Isoform Mitochondrial of Fumarate hydratase, mitochondrial | 82 | 54714 | 1 |
| IPI00198444 | Gatm Glycine amidinotransferase, mitochondrial | 82 | 48724 | 2 |
| IPI00200883 | Gls2 Isoform 2 of Glutaminase liver isoform, mitochondrial | 58 | 60021 | 6 |
| IPI00324633 | Glud1 Glutamate dehydrogenase 1, mitochondrial | 173 | 61719 | 16 |
| IPI00205157 | Hadh Hydroxyacyl-coenzyme A dehydrogenase, mitochondrial | 182 | 34540 | 21 |
| IPI00202658 | Hibadh 3-hydroxyisobutyrate dehydrogenase, mitochondrial | 62 | 35679 | 1 |
| IPI00210444 | Hmgcs2 Hydroxymethylglutaryl-CoA synthase, mitochondrial | 572 | 57332 | 46 |
| IPI00339148 | Hspd1 60 kDa heat shock protein, mitochondrial | 153 | 61088 | 12 |
| IPI00194324 | Pdhb Pyruvate dehydrogenase E1 component subunit beta, mitochondrial | 102 | 39299 | 2 |
| IPI00208215 | Prdx3 Thioredoxin-dependent peroxide reductase, mitochondrial | 48 | 28563 | 1 |
| IPI00785564 | Sdhb Succinate dehydrogenase [ubiquinone] iron-sulfur subunit, mitochondrial | 138 | 32607 | 3 |
| **unknown localization structure and function** | |  |  |  |
| IPI00765735 | LOC291545similar to Glyceraldehyde-3-phosphate dehydrogenase | 86 | 21456 | 4 |
| IPI00358127 | Actr3b similar to ARP3 actin-related protein 3 homolog B | 110 | 14180 | 4 |
| IPI00211013 | Adap1 Centaurin alpha | 72 | 77868 | 1 |
| IPI00369534 | Alkbh5 hypothetical protein | 227 | 25708 | 11 |
| IPI00207010 | Baat Bile acid-CoA:amino acid N-acyltransferase | 56 | 10922 | 2 |
| IPI00206466 | Csnk1a1 Csnk1a1 protein | 53 | 107293 | 10 |
| IPI00231826 | Dab2 26 kDa protein | 51 | 16924 | 1 |
| IPI00361832 | Dhx15 similar to Putative pre-mRNA-splicing factor ATP-dependent RNA helicase DHX15 | 49 | 15446 | 6 |
| IPI00366091 | Eps8l2 Eps8l2 protein | 105 | 61913 | 4 |
| IPI00365852 | Erh RCG20904, isoform CRA_b | 85 | 39118 | 5 |
| IPI00409539 | Flna 281 kDa protein | 85 | 8000 | 4 |
| IPI00201333 | Ganab 107 kDa protein | 97 | 46777 | 6 |
| IPI00206254 | Ggt1 Gamma-glutamyltranspeptidase 1 | 119 | 24680 | 3 |
| IPI00366719 | Gnat1 rod-type transducin alpha subunit | 44 | 402600 | 19 |
| IPI00199482 | Gstm4 RCG29014, isoform CRA_a | 49 | 48616 | 3 |
| IPI00210360 | Hspg2 394 kDa protein | 57 | 26305 | 3 |
| IPI00361944 | LOC291863RCG63121, isoform CRA_a | 72 | 80665 | 3 |
| IPI00765011 | LOC295810similar to Actin, cytoplasmic 2 | 49 | 48117 | 4 |
| IPI00421412 | LOC296300LRRGT00145 | 85 | 54225 | 13 |
| IPI00392753 | LOC298109RCG32004transpoorter | 55 | 34306 | 7 |
| IPI00568085 | LOC29811119 kDa protein | 448 | 91717 | 31 |
| IPI00569362 | LOC29811120 kDa protein | 482 | 62399 | 27 |
| IPI00200422 | LOC298111Alpha2u globulin]lipocalin family | 57 | 18028 | 3 |
| IPI00464895 | LOC298116Rat alpha-2u-globulin | 60 | 33836 | 7 |
| IPI00471526 | LOC298795Similar to 14-3-3 protein sigma | 48 | 44074 | 6 |
| IPI00370776 | LOC303448Similar to glyceraldehyde-3-phosphate dehydrogenase | 192 | 23703 | 7 |
| IPI00421332 | LOC306079LRRGT00066 | 74 | 35360 | 4 |
| IPI00767374 | LOC360570LOC360570 protein | 95 | 28180 | 5 |
| IPI00568245 | LOC360570similar to myosin XVIIIa | 58 | 171823 | 7 |
| IPI00763003 | LOC365050similar to heat shock protein 1, alpha | 47 | 40383 | 6 |
| IPI00569888 | LOC36511425 kDa protein | 94 | 166177 | 13 |
| IPI00565587 | LOC365114similar to High mobility group protein 1 | 75 | 34315 | 4 |
| IPI00206851 | LOC366380Alpha 2u-globulin (Fragment) | 57 | 18617 | 4 |
| IPI00776965 | LOC366380similar to alpha-2u globulin PGCL2 | 46 | 44768 | 4 |
| IPI00768602 | LOC499706similar to Heat shock protein HSP 90-beta | 148 | 275391 | 24 |
| IPI00563431 | LOC499896Glyceraldehyde 3-phosphate dehydrogenase (Fragment) | 100 | 69759 | 6 |
| IPI00382256 | LOC502176Ab1-331 | 125 | 23370 | 5 |
| IPI00765152 | LOC502627similar to L-lactate dehydrogenase A chain | 73 | 43604 | 16 |
| IPI00921682 | LOC641316delta-1-pyrroline-5-carboxylate dehydrogenase, mitochondrial | 99 | 27729 | 2 |
| IPI00768167 | LOC679312similar to beta tubulin 1, class VI | 59 | 16980 | 4 |
| IPI00566640 | LOC679344;LOC685520 similar to High mobility group protein 1 | 148 | 274022 | 23 |
| IPI00557035 | LOC679451;Hmg1l1 high mobility group box 1 like | 60 | 11357 | 1 |
| IPI00763565 | LOC679594;LOC682397 similar to polyubiquitin | 248 | 20924 | 14 |
| IPI00781565 | LOC679950;Hist1h3f;Hist2h3c2;LOC684841;LOC684762 histone cluster 2, H3c2 | 53 | 38570 | 4 |
| IPI00214497 | LOC680063Histone H2A type 1-F | 53 | 72690 | 6 |
| IPI00767397 | LOC680385similar to Sjogren syndrome antigen B | 202 | 21007 | 13 |
| IPI00764426 | LOC681426similar to alpha-2u globulin PGCL4 isoform 1 | 52 | 27956 | 6 |
| IPI00564375 | LOC681718;LOC680968 similar to High mobility group protein 1 | 81 | 21084 | 5 |
| IPI00563236 | LOC682450;LOC680498 similar to CG31613-PA | 113 | 39561 | 7 |
| IPI00767531 | LOC682465;LOC680217 similar to Ferritin light chain 2 | 249 | 24945 | 16 |
| IPI00200762 | LOC682787similar to SMT3 suppressor of mif two 3 homolog 2 isoform 2 | 89 | 34274 | 4 |
| IPI00768849 | LOC683062;LOC679923 similar to voltage-dependent anion channel 1 | 57 | 14444 | 2 |
| IPI00765663 | LOC683099similar to zinc finger protein 341 isoform 1 | 228 | 21190 | 11 |
| IPI00359623 | LOC683474similar to aldehyde dehydrogenase 8 family, member A1 isoform 2 | 81 | 20772 | 4 |
| IPI00766218 | LOC683536similar to ubiquitin-conjugating enzyme E2N | 71 | 11360 | 14 |
| IPI00763070 | LOC683761similar to RT1 class I, CE11 | 68 | 44028 | 3 |
| IPI00765243 | LOC684280similar to Class I histocompatibility antigen, Non-RT1.A alpha-1 chain precursor | 135 | 24516 | 8 |
| IPI00589869 | LOC684609;LOC690495 similar to ribosomal protein S15a | 219 | 25033 | 10 |
| IPI00764478 | LOC684800similar to stromal membrane-associated protein 1 | 135 | 15824 | 8 |
| IPI00767428 | LOC684819similar to H3 histone, family 2 isoform 2 | 78 | 25043 | 5 |
| IPI00763706 | LOC685160similar to spermatogenesis associated glutamate (E)-rich protein 4d | 59 | 234229 | 9 |
| IPI00371093 | LOC68526969 kDa protein | 80 | 13991 | 12 |
| IPI00554102 | LOC685482;LOC259245 similar to alpha2u globulin | 71 | 22917 | 2 |
| IPI00769283 | LOC686250similar to unconventional myosin Myr2 I heavy chain | 89 | 24874 | 5 |
| IPI00769127 | LOC687144similar to RT1 class I, CE4 | 83 | 9605 | 6 |
| IPI00766278 | LOC687711similar to small nuclear ribonucleoprotein D3 | 86 | 23807 | 7 |
| IPI00766578 | LOC688226similar to UDP-glucuronosyltransferase 2B2 precursor | 54 | 73898 | 1 |
| IPI00764687 | LOC688319similar to RAS-related C3 botulinum substrate 3 | 384 | 11417 | 23 |
| IPI00569329 | LOC688583;LOC678896 similar to High mobility group protein 4 | 56 | 8610 | 2 |
| IPI00559336 | LOC688815similar to prohibitin | 70 | 45996 | 9 |
| IPI00781321 | LOC68886910 kDa protein | 124 | 24890 | 12 |
| IPI00569279 | LOC690102hypothetical protein LOC690102 | 77 | 133584 | 3 |
| IPI00766968 | LOC690226similar to dehydrogenase/reductase (SDR family) member 7 | 81 | 17200 | 2 |
| IPI00362298 | LOC690675;LOC684936 similar to Cytochrome c, somatic | 63 | 14021 | 5 |
| IPI00766000 | LOC690693similar to DDX19 homolog | 76 | 15484 | 4 |
| IPI00765570 | LOC690940;LOC689398 similar to High mobility group protein 1 | 250 | 29612 | 17 |
| IPI00763798 | LOC691781similar to Tubulin alpha-2 chain | 55 | 36738 | 8 |
| IPI00382173 | Macrod1 LRP16-like protein | 59 | 196264 | 9 |
| IPI00207766 | Mgst3 similar to microsomal glutathione S-transferase 3 | 117 | 46655 | 11 |
| IPI00371266 | Naca Nascent-polypeptide-associated complex alpha polypeptide (Predicted), isoform CRA_b | 74 | 16936 | 19 |
| IPI00557463 | PNN | 85 | 24338 | 6 |
| IPI00421405 | Podn LRRGT00160 | 48 | 15209 | 1 |
| IPI00555299 | Pon1 serum paraoxonase/arylesterase 1 | 69 | 51068 | 5 |
| IPI00366588 | Rb1cc1 170 kDa protein | 65 | 27896 | 15 |
| IPI00766092 | RGD1310016 similar to CG9063-PA | 82 | 13285 | 5 |
| IPI00362963 | RGD1310224 Similar to RIKEN cDNA 1810022C23 | 124 | 22309 | 7 |
| IPI00764799 | RGD1559455 similar to Alpha-3 catenin | 88 | 14849 | 3 |
| IPI00362347 | RGD1559590 similar to glyceraldehyde-3-phosphate dehydrogenase | 52 | 57159 | 3 |
| IPI00558079 | RGD1559704 similar to glyceraldehyde-3-phosphate dehydrogenase | 78 | 21018 | 4 |
| IPI00372910 | RGD1560402 similar to Phosphoglycerate kinase 1 | 105 | 62245 | 11 |
| IPI00561379 | RGD1560523 similar to S-adenosylmethionine synthetase isoform type-2 | 81 | 20788 | 4 |
| IPI00559222 | RGD1560584 similar to High mobility group protein 1 (HMG-1) B1) (Amphoterin) (Heparin-binding protein p30) isoform 2 | 146 | 59163 | 19 |
| IPI00949773 | RGD1560687 similar to Ferritin light chain 1 | 175 | 46477 | 8 |
| IPI00373541 | RGD1560797 similar to glyceraldehyde-3-phosphate dehydrogenase | 69 | 51068 | 5 |
| IPI00204128 | RGD1561381 similar to microsomal glutathione S-transferase 3 | 117 | 46655 | 11 |
| IPI00366490 | RGD1561812 similar to Retinol dehydrogenase 2 | 60 | 26160 | 3 |
| IPI00364052 | RGD1562091 hypothetical protein LOC292690 | 60 | 10609 | 2 |
| IPI00564933 | RGD1562312 similar to High mobility group protein 1 | 50 | 25052 | 1 |
| IPI00561052 | RGD1562378;Hist2h4 | 49 | 28847 | 9 |
| IPI00656375 | RGD1562758 similar to glyceraldehyde-3-phosphate dehydrogenase | 46 | 68001 | 4 |
| IPI00561747 | RGD1563668 similar to High mobility group protein 1 | 77 | 33326 | 7 |
| IPI00359732 | RGD1564064 similar to Glyceraldehyde-3-phosphate dehydrogenase | 176 | 29168 | 14 |
| IPI00944221 | RGD1564257 hypothetical protein LOC500595 | 88 | 36271 | 4 |
| IPI00558056 | RGD1564560 hypothetical protein LOC500988 | 55 | 303497 | 6 |
| IPI00360618 | RGD1564894 RGD1564894 protein | 67 | 192706 | 42 |
| IPI00768929 | RGD1565145 similar to cysteine sulfinic acid decarboxylase | 70 | 44990 | 8 |
| IPI00365813 | RGD1565338 similar to Voltage-dependent anion-selective channel protein 1 | 78 | 47734 | 2 |
| IPI00387880 | RGD1565416 272 kDa protein | 45 | 73593 | 2 |
| IPI00370137 | RGD1565416 similar to talin 2 | 47 | 40784 | 4 |
| IPI00388918 | RGD1565438 similar to ATP synthase, H+ transporting, mitochondrial F0 complex, subunit G | 51 | 125088 | 1 |
| IPI00560599 | RGD1566189 similar to Ferritin light chain 2 | 98 | 60761 | 2 |
| IPI00368704 | RGD1566313 similar to Murinoglobulin 1 homolog | 72 | 22055 | 1 |
| IPI00778900 | SFXN | 123 | 62083 | 8 |
| IPI00337118 | Stab2 similar to stabilin-2 | 55 | 35736 | 2 |
| IPI00555188 | Stard10 START domain containing 10, isoform CRA_b | 56 | 35871 | 5 |
| IPI00191919 | Susd2 Susd2 protein | 75 | 36032 | 3 |
| IPI00562292 | Tapbp 47 kDa protein | 154 | 37075 | 8 |
| IPI00191354 | TPM3 | 62 | 36769 | 2 |
| IPI00365393 | Tppp tubulin polymerization promoting protein | 288 | 37490 | 14 |
| IPI00781838 | - 10 kDa protein | 79 | 37886 | 12 |
| IPI00360148 | - 104 kDa protein | 69 | 39780 | 2 |
| IPI00777298 | - 109 kDa protein | 73 | 39678 | 6 |
| IPI00568896 | - 11 kDa protein | 57 | 39755 | 2 |
| IPI00778180 | - 11 kDa protein | 77 | 40274 | 3 |
| IPI00561016 | - 128 kDa protein | 83 | 41744 | 4 |
| IPI00560602 | - 13 kDa protein | 58 | 41576 | 7 |
| IPI00782125 | - 13 kDa protein | 52 | 43154 | 8 |
| IPI00207486 | - 14 kDa protein | 174 | 42405 | 10 |
| IPI00326699 | - 15 kDa protein | 155 | 44617 | 28 |
| IPI00475811 | - 15 kDa protein | 64 | 45115 | 12 |
| IPI00476722 | - 15 kDa protein | 75 | 46171 | 8 |
| IPI00778245 | - 159 kDa protein | 66 | 45968 | 2 |
| IPI00362291 | - 16 kDa protein | 97 | 49544 | 21 |
| IPI00780795 | - 161 kDa protein | 75 | 5299 | 6 |
| IPI00911369 | - 162 kDa protein | 221 | 50115 | 22 |
| IPI00189362 | - 17 kDa protein | 251 | 50418 | 34 |
| IPI00370899 | - 17 kDa protein | 121 | 50158 | 11 |
| IPI00781561 | - 18 kDa protein | 56 | 52246 | 2 |
| IPI00189519 | - 19 kDa protein | 55 | 57323 | 7 |
| IPI00400550 | - 19 kDa protein | 146 | 57629 | 12 |
| IPI00950425 | - 2 kDa protein | 78 | 59425 | 3 |
| IPI00394470 | - 20 kDa protein | 106 | 6479 | 9 |
| IPI00561205 | - 20 kDa protein | 139 | 60262 | 4 |
| IPI00565010 | - 20 kDa protein | 94 | 62449 | 5 |
| IPI00565818 | - 21 kDa protein | 62 | 64915 | 4 |
| IPI00388302 | - 22 kDa protein | 342 | 69213 | 15 |
| IPI00565247 | - 23 kDa protein | 714 | 71372 | 36 |
| IPI00390751 | - 24 kDa protein | 96 | 77410 | 14 |
| IPI00392981 | - 24 kDa protein | 100 | 81278 | 12 |
| IPI00782551 | - 24 kDa protein | 78 | 19768 | 4 |
| IPI00368131 | - 25 kDa protein | 79 | 21661 | 1 |
| IPI00765773 | - 26 kDa protein | 135 | 21323 | 5 |
| IPI00370348 | - 28 kDa protein | 56 | 37339 | 4 |
| IPI00776429 | - 29 kDa protein | 417 | 64107 | 19 |
| IPI00372308 | - 30 kDa protein | 62 | 11496 | 2 |
| IPI00949396 | - 31 kDa protein | 59 | 17199 | 5 |
| IPI00206268 | - 32 kDa protein | 284 | 42089 | 7 |
| IPI00781071 | - 34 kDa protein | 51 | 549884 | 4 |
| IPI00366141 | - 35 kDa protein | 80 | 22907 | 4 |
| IPI00950601 | - 36 kDa protein | 184 | 35444 | 9 |
| IPI00368629 | - 37 kDa protein | 57 | 13187 | 2 |
| IPI00564357 | - 37 kDa protein | 50 | 14311 | 5 |
| IPI00562827 | - 43 kDa protein | 50 | 14304 | 3 |
| IPI00391519 | - 44 kDa protein | 57 | 14881 | 1 |
| IPI00564678 | - 45 kDa protein | 632 | 14739 | 25 |
| IPI00559851 | - 46 kDa protein | 51 | 14634 | 1 |
| IPI00568311 | - 46 kDa protein | 55 | 15875 | 2 |
| IPI00360881 | - 49 kDa protein | 632 | 16399 | 25 |
| IPI00361239 | - 49 kDa protein | 97 | 15813 | 4 |
| IPI00778110 | - 5 kDa protein | 49 | 16817 | 1 |
| IPI00190713 | - 50 kDa protein | 84 | 16766 | 3 |
| IPI00390302 | - 57 kDa protein | 154 | 17809 | 12 |
| IPI00949883 | - 59 kDa protein | 59 | 17846 | 3 |
| IPI00950440 | - 6 kDa protein | 57 | 18629 | 3 |
| IPI00567316 | - 60 kDa protein | 73 | 1609 | 3 |
| IPI00778786 | - 62 kDa protein | 307 | 19927 | 11 |
| IPI00782078 | - 65 kDa protein | 219 | 20585 | 10 |
| IPI00778270 | - 68 kDa protein | 152 | 19855 | 8 |
| IPI00561262 | - 69 kDa protein | 51 | 20072 | 3 |
| IPI00949898 | - 71 kDa protein | 47 | 20508 | 8 |
| IPI00779120 | - 73 kDa protein | 135 | 21201 | 5 |
| IPI00392830 | - 77 kDa protein | 124 | 21099 | 6 |
| IPI00951899 | - 81 kDa protein | 56 | 21363 | 2 |
