# Supplementary material for: Profiling of kidney vascular endothelial cell plasma membrane proteins by liquid chromatography–tandem mass spectrometry
Source: Clin Exp Nephrol. 2012 Dec 11;17(3):327–37. doi: 10.1007/s10157-012-0708-1 (PMC3684716; doi:10.1007/s10157-012-0708-1)
Supplement: Supplementary file 2 — Supplementary material 2 (DOCX 160 kb) [file 10157_2012_708_MOESM2_ESM.docx]

| **Supplementary Table 2** | |  |  |  |
| --- | --- | --- | --- | --- |
| **Entire kidney proteome** | |  |  |  |
| **prot_acc** | **prot_desc** | **prot_**  **score** | **prot_**  **mass** | **prot_**  **matches** |
| IPI00551812 | Atp5b ATP synthase subunit beta, mitochondrial | 4345 | 57308 | 182 |
| IPI00396910 | Atp5a1 ATP synthase subunit alpha, mitochondrial | 3234 | 61050 | 139 |
| IPI00326305 | Atp1a1 Sodium/potassium-transporting ATPase subunit alpha-1 | 1735 | 115390 | 81 |
| IPI00205325 | Lrp2 Low-density lipoprotein receptor-related protein 2 | 1641 | 525860 | 127 |
| IPI00287835 | Hba-a2 Hemoglobin subunit alpha-1/2 | 1289 | 15490 | 120 |
| IPI00765205 | Col6a3 procollagen, type VI, alpha 3 | 1242 | 244175 | 85 |
| IPI00958161 | LOC684969 actin, gamma 1 propeptide-like | 1219 | 42109 | 98 |
| IPI00197696 | Mdh2 Malate dehydrogenase, mitochondrial | 1069 | 36736 | 27 |
| IPI00896224 | Actg1 Actin, cytoplasmic 2 | 984 | 42583 | 77 |
| IPI00195123 | Atp5o ATP synthase subunit O, mitochondrial | 970 | 24243 | 43 |
| IPI00471911 | Aldob Fructose-bisphosphate aldolase B | 882 | 40582 | 46 |
| IPI00958044 | Hist1h2ai histone cluster 1, H2ae-like | 845 | 28038 | 78 |
| IPI00960040 | LOC680097 histone cluster 1, H2ae-like | 845 | 52095 | 80 |
| IPI00205018 | Aldh6a1 Methylmalonate-semialdehyde dehydrogenase [acylating], mitochondrial | 810 | 59147 | 47 |
| IPI00201413 | Acaa2 3-ketoacyl-CoA thiolase, mitochondrial | 736 | 42834 | 34 |
| IPI00210435 | Pc Pyruvate carboxylase, mitochondrial | 699 | 132189 | 54 |
| IPI00210360 | Hspg2 394 kDa protein | 670 | 396444 | 40 |
| IPI00188924 | Uqcrc2 Cytochrome b-c1 complex subunit 2, mitochondrial | 649 | 49527 | 35 |
| IPI00189813 | Acta1 Actin, alpha skeletal muscle | 626 | 42841 | 69 |
| IPI00194087 | Actc1 Actin, alpha cardiac muscle 1 | 626 | 42809 | 69 |
| IPI00231245 | Hao2 Hydroxyacid oxidase 2 | 607 | 40123 | 29 |
| IPI00196107 | Atp5f1 ATP synthase subunit b, mitochondrial | 575 | 29797 | 32 |
| IPI00209113 | Myh9 Myosin-9 | 545 | 235014 | 45 |
| IPI00203317 | Cyp4a2 Cytochrome P450 4A2 | 539 | 59265 | 32 |
| IPI00231451 | Atp1a3 Sodium/potassium-transporting ATPase subunit alpha-3 | 518 | 114071 | 28 |
| IPI00230862 | Anpep Aminopeptidase N | 517 | 111444 | 24 |
| IPI00763746 | LOC683474;Aldh8a1 aldehyde dehydrogenase 8 family, member A1-like isoform 1 | 517 | 48030 | 45 |
| IPI00209363 | Slc25a10 Solute carrier family 25 (Mitochondrial carrier | 499 | 32036 | 38 |
| IPI00191737 | Alb Serum albumin | 491 | 71008 | 27 |
| IPI00203318 | Cyp4a3 Cytochrome P450 4A14 | 486 | 59571 | 24 |
| IPI00200659 | Sdha Succinate dehydrogenase [ubiquinone] flavoprotein subunit, mitochondrial | 471 | 72774 | 27 |
| IPI00363849 | Lamc1 laminin, gamma 1 | 467 | 180670 | 20 |
| IPI00785564 | Sdhb Succinate dehydrogenase [ubiquinone] iron-sulfur subunit, mitochondrial | 440 | 32884 | 29 |
| IPI00952273 | Sptbn1 Sptbn1 protein | 437 | 275310 | 41 |
| IPI00767147 | Eno1-ps1 similar to Alpha-enolase | 434 | 55567 | 19 |
| IPI00197129 | Acta2 Actin, aortic smooth muscle | 418 | 42799 | 59 |
| IPI00421539 | Aco2 Aconitate hydratase, mitochondrial | 415 | 87659 | 29 |
| IPI00560160 | Actg2 43 kDa protein | 414 | 43794 | 42 |
| IPI00949922 | Lama1 laminin, alpha 1 | 409 | 202399 | 32 |
| IPI00193983 | Cltc Clathrin heavy chain 1 | 397 | 195648 | 21 |
| IPI00190557 | Phb2 Prohibitin-2 | 395 | 34109 | 21 |
| IPI00370387 | RGD1560058 hypothetical protein LOC287559 | 391 | 30989 | 11 |
| IPI00388191 | - 34 kDa protein | 384 | 34755 | 12 |
| IPI00569815 | RGD1565238 glyceraldehyde-3-phosphate dehydrogenase-like, partial | 375 | 38336 | 11 |
| IPI00358033 | Ndufs1 NADH-ubiquinone oxidoreductase 75 kDa subunit, mitochondrial | 371 | 81125 | 30 |
| IPI00365545 | Dld Dihydrolipoyl dehydrogenase, mitochondrial | 370 | 54574 | 19 |
| IPI00921682 | Aldh4a1 delta-1-pyrroline-5-carboxylate dehydrogenase, mitochondrial | 346 | 62245 | 30 |
| IPI00968490 | Tas1r2 Ba1-651 | 346 | 165451 | 31 |
| IPI00324633 | Glud1 Glutamate dehydrogenase 1, mitochondrial | 344 | 62839 | 32 |
| IPI00564015 | - 35 kDa protein | 342 | 35670 | 22 |
| IPI00231631 | Eno3 Beta-enolase | 338 | 48619 | 12 |
| IPI00373076 | Atp6v1a ATPase, H+ transporting, lysosomal V1 subunit A | 328 | 69727 | 22 |
| IPI00196620 | Cubn Cubilin | 328 | 402430 | 30 |
| IPI00212622 | Hadha Trifunctional enzyme subunit alpha, mitochondrial | 325 | 85494 | 30 |
| IPI00470325 | Pyroxd2 Pyridine nucleotide-disulfide oxidoreductase domain-containing protein 2 | 324 | 63872 | 16 |
| IPI00231742 | Cat Catalase | 320 | 61010 | 22 |
| IPI00211756 | Phb Prohibitin | 317 | 30318 | 18 |
| IPI00763566 | - similar to Glyceraldehyde-3-phosphate dehydrogenase | 316 | 26897 | 12 |
| IPI00561918 | - 34 kDa protein | 314 | 35098 | 17 |
| IPI00360356 | Actbl2 actin, beta-like 2 | 314 | 42710 | 22 |
| IPI00213463 | Actn4 Alpha-actinin-4 | 308 | 107258 | 22 |
| IPI00187747 | Rap1a Ras-related protein Rap-1A | 307 | 21705 | 8 |
| IPI00358163 | Slc25a13 similar to Calcium-binding mitochondrial carrier protein Aralar2 | 307 | 76115 | 22 |
| IPI00199305 | Atp6v1b2 V-type proton ATPase subunit B, brain isoform | 300 | 57461 | 20 |
| IPI00464440 | Sfxn1 Sideroflexin 1 | 296 | 36124 | 14 |
| IPI00215574 | Dci 3,2-trans-enoyl-CoA isomerase, mitochondrial | 285 | 33094 | 16 |
| IPI00213584 | Agxt2 Alanine--glyoxylate aminotransferase 2, mitochondrial | 283 | 58366 | 18 |
| IPI00471539 | Suclg2 Suclg2 protein | 282 | 47244 | 16 |
| IPI00886470 | Hsd17b10 3-hydroxyacyl-CoA dehydrogenase type-2 | 281 | 28860 | 19 |
| IPI00324302 | Acat1 Acetyl-CoA acetyltransferase, mitochondrial | 277 | 46086 | 13 |
| IPI00357924 | Idh3B Isocitrate dehydrogenase [NAD] subunit beta, mitochondrial | 276 | 43445 | 5 |
| IPI00776619 | Spna2 Spectrin alpha chain, brain | 275 | 292977 | 40 |
| IPI00202616 | Ndufs3 NADH dehydrogenase (ubiquinone) Fe-S protein 3 | 266 | 30767 | 15 |
| IPI00339148 | Hspd1 60 kDa heat shock protein, mitochondrial | 265 | 63240 | 16 |
| IPI00364046 | Tuba1c Tubulin alpha-1C chain | 261 | 50680 | 17 |
| IPI00476458 | Gpx3 Glutathione peroxidase 3 | 257 | 26148 | 30 |
| IPI00372839 | Col6a2 collagen, type VI, alpha 2 | 254 | 111785 | 22 |
| IPI00948259 | Mthfd1 Methylenetetrahydrofolate dehydrogenase (NADP+ dependent) 1, methenyltetrahydrofolate cyclohydrolase, formyltetrahydrofolate synthetase | 254 | 103580 | 11 |
| IPI00207217 | Echs1 Enoyl-CoA hydratase, mitochondrial | 250 | 32442 | 20 |
| IPI00515768 | Glyat Glycine N-acyltransferase | 249 | 34823 | 11 |
| IPI00327697 | Dpep1 Dipeptidase 1 | 248 | 46095 | 15 |
| IPI00679256 | Es22;LOC100125372 Liver carboxylesterase 4 | 247 | 63731 | 14 |
| IPI00194974 | Hnrnpk Uncharacterized protein | 244 | 51281 | 12 |
| IPI00230859 | Akr1a1 Alcohol dehydrogenase [NADP+] | 240 | 37386 | 26 |
| IPI00205693 | Atp1a2 Sodium/potassium-transporting ATPase subunit alpha-2 | 240 | 114511 | 11 |
| IPI00950342 | - 39 kDa protein | 239 | 39958 | 23 |
| IPI00190577 | Lama5 similar to Laminin alpha-5 chain precursor | 239 | 407623 | 22 |
| IPI00339167 | Tuba1b Tubulin alpha-1B chain | 238 | 51065 | 15 |
| IPI00208422 | Dpp4 Dipeptidyl peptidase 4 | 235 | 89925 | 22 |
| IPI00209908 | Mt-co2 Cytochrome c oxidase subunit 2 | 233 | 26140 | 21 |
| IPI00777695 | Immt 87 kDa protein | 232 | 89469 | 7 |
| IPI00205745 | Prdx5 Isoform Mitochondrial of Peroxiredoxin-5, mitochondrial | 231 | 22507 | 12 |
| IPI00915569 | Gstt3;Gstt1 glutathione S-transferase, theta 3 | 229 | 23753 | 11 |
| IPI00371853 | Col6a1 similar to Collagen alpha-1(VI) chain precursor | 227 | 111103 | 15 |
| IPI00339162 | RGD1563601 rCG29914-like | 226 | 32285 | 15 |
| IPI00200344 | Scpep1 Retinoid-inducible serine carboxypeptidase | 226 | 51427 | 14 |
| IPI00358441 | Ndufa9 Ndufa9 protein | 224 | 43349 | 9 |
| IPI00560967 | Npl2 N-acetylneuraminate pyruvate lyase 2 | 223 | 35002 | 9 |
| IPI00650097 | Acaa1a 3-ketoacyl-CoA thiolase A, peroxisomal | 221 | 45310 | 13 |
| IPI00370596 | Acaa1b 3-ketoacyl-CoA thiolase B, peroxisomal | 221 | 44306 | 12 |
| IPI00364780 | Atp6v1h ATPase, H+ transporting, lysosomal V1 subunit H | 221 | 51988 | 10 |
| IPI00764690 | Mtch2 mitochondrial carrier homolog 2 | 221 | 33979 | 14 |
| IPI00768041 | Slc25a45 similar to mitochondrial hepatocellular carcinoma-downregulated carrier protein | 214 | 32004 | 6 |
| IPI00198444 | Gatm Glycine amidinotransferase, mitochondrial | 213 | 49243 | 12 |
| IPI00202543 | Hk1 Hexokinase-1 | 213 | 105094 | 16 |
| IPI00363265 | Hspa9 Stress-70 protein, mitochondrial | 213 | 76091 | 20 |
| IPI00766788 | Oxct1 Succinyl-CoA:3-ketoacid-coenzyme A transferase 1, mitochondrial | 213 | 57845 | 10 |
| IPI00212776 | Rps3 40S ribosomal protein S3 | 213 | 26828 | 12 |
| IPI00454367 | Lmna Lmna protein | 212 | 51732 | 15 |
| IPI00555265 | Nnt Nicotinamide nucleotide transhydrogenase | 211 | 116462 | 21 |
| IPI00211593 | Sod2 Superoxide dismutase [Mn], mitochondrial | 210 | 25347 | 4 |
| IPI00215093 | Ogdh 2-oxoglutarate dehydrogenase, mitochondrial | 209 | 118372 | 29 |
| IPI00231568 | Tpm1 Isoform 8 of Tropomyosin alpha-1 chain | 209 | 29647 | 6 |
| IPI00230897 | Hbb Hemoglobin subunit beta-1 | 208 | 16485 | 13 |
| IPI00189819 | Actb Actin, cytoplasmic 1 | 207 | 42527 | 21 |
| IPI00948302 | Atp5c1 ATP synthase gamma chain | 205 | 34136 | 16 |
| IPI00765231 | Pm20d1 peptidase M20 domain containing 1 | 205 | 56568 | 10 |
| IPI00949883 | - 59 kDa protein | 204 | 61177 | 15 |
| IPI00210920 | Got2 Aspartate aminotransferase, mitochondrial | 204 | 48574 | 28 |
| IPI00202238 | Ndufb10 NADH dehydrogenase (ubiquinone) 1 beta subcomplex, 10 | 204 | 21404 | 8 |
| IPI00365929 | Pdia6 protein disulfide-isomerase A6 | 202 | 50149 | 7 |
| IPI00209115 | Slc25a3 Solute carrier family 25 (Mitochondrial carrier | 202 | 40542 | 25 |
| IPI00326412 | Eno2 Gamma-enolase | 199 | 48272 | 5 |
| IPI00332042 | Aldh1a1 Retinal dehydrogenase 1 | 198 | 56101 | 6 |
| IPI00197568 | Gdi2 Rab GDP dissociation inhibitor beta | 198 | 52096 | 10 |
| IPI00198467 | Hadhb Trifunctional enzyme subunit beta, mitochondrial | 198 | 52844 | 14 |
| IPI00358005 | Chdh Choline dehydrogenase | 197 | 67465 | 17 |
| IPI00207146 | Hbb-b1 Zero beta-1 globin | 197 | 16069 | 42 |
| IPI00191707 | Pdha1 Pyruvate dehydrogenase E1 component subunit alpha, somatic form, mitochondrial | 197 | 44188 | 14 |
| IPI00197684 | Xpnpep2 X-prolyl aminopeptidase (Aminopeptidase P) 2, membrane-bound | 197 | 77107 | 17 |
| IPI00211225 | Acadl Long-chain specific acyl-CoA dehydrogenase, mitochondrial | 196 | 48961 | 5 |
| IPI00781133 | Aco1 99 kDa protein | 194 | 101088 | 13 |
| IPI00197770 | Aldh2 Aldehyde dehydrogenase, mitochondrial | 192 | 57786 | 9 |
| IPI00231650 | Hist1h1d Histone H1.2 | 192 | 21974 | 20 |
| IPI00763589 | LOC684681 histone cluster 1, H1c-like | 192 | 21304 | 23 |
| IPI00763910 | LOC684747 similar to 60 kDa heat shock protein, mitochondrial precursor | 192 | 96391 | 11 |
| IPI00367152 | Ndufv2 NADH dehydrogenase [ubiquinone] flavoprotein 2, mitochondrial | 192 | 27834 | 14 |
| IPI00211616 | Slc3a2 4F2 cell-surface antigen heavy chain | 191 | 59197 | 9 |
| IPI00198641 | Acsm2 Acyl-coenzyme A synthetase ACSM2, mitochondrial | 190 | 65824 | 10 |
| IPI00204311 | Ak3l1 Adenylate kinase isoenzyme 4, mitochondrial | 190 | 25703 | 27 |
| IPI00327991 | Cyp2c23 Cytochrome P450 2C23 | 190 | 58117 | 10 |
| IPI00231745 | Fbp1 Fructose-1,6-bisphosphatase 1 | 190 | 40616 | 11 |
| IPI00471577 | Uqcrc1 Cytochrome b-c1 complex subunit 1, mitochondrial | 190 | 53374 | 27 |
| IPI00198966 | Abat 50 kDa protein | 188 | 51341 | 10 |
| IPI00188330 | Ndufs8 Ndufs8 protein | 188 | 24342 | 12 |
| IPI00561513 | Ndufa10 NADH dehydrogenase [ubiquinone] 1 alpha subcomplex subunit 10, mitochondrial | 187 | 41500 | 12 |
| IPI00231261 | Slc25a11 Mitochondrial 2-oxoglutarate/malate carrier protein | 187 | 34910 | 11 |
| IPI00390542 | - 36 kDa protein | 186 | 37052 | 16 |
| IPI00766273 | LOC684828 histone cluster 1, H1d-like | 185 | 22220 | 26 |
| IPI00203528 | Stoml2 Stomatin-like protein 2 | 185 | 39121 | 9 |
| IPI00231118 | Calb1 Calbindin | 184 | 31050 | 12 |
| IPI00564034 | - 16 kDa protein | 183 | 16163 | 11 |
| IPI00557715 | - 46 kDa protein | 183 | 47019 | 11 |
| IPI00765861 | - similar to Glyceraldehyde-3-phosphate dehydrogenase | 183 | 16904 | 10 |
| IPI00193485 | Idh2 Isocitrate dehydrogenase [NADP], mitochondrial | 183 | 52526 | 17 |
| IPI00206222 | - 29 kDa protein | 182 | 29781 | 6 |
| IPI00204808 | Mep1b Meprin A subunit beta | 182 | 80155 | 8 |
| IPI00393508 | Hist1h2bb;LOC684647 similar to Histone H2B 291B | 180 | 14774 | 6 |
| IPI00766320 | LOC682355;Hist1h2bm histone cluster 1, H2bm | 180 | 16287 | 7 |
| IPI00400573 | Tubb2c Tubulin beta-2C chain | 180 | 50414 | 28 |
| IPI00365705 | Atp4a potassium-transporting ATPase alpha chain 1 | 179 | 116098 | 9 |
| IPI00421389 | Ndrg1 Protein NDRG1 | 175 | 43529 | 4 |
| IPI00199636 | Canx Calnexin | 173 | 69664 | 4 |
| IPI00362160 | Tubb3 Tubulin beta-3 chain | 172 | 51074 | 19 |
| IPI00212934 | Slc5a2 Sodium/glucose cotransporter 2 | 171 | 73516 | 10 |
| IPI00197579 | Tubb5 Isoform 1 of Tubulin beta-5 chain | 170 | 50284 | 23 |
| IPI00464791 | Acy1 Aminoacylase-1A | 169 | 46549 | 23 |
| IPI00364431 | Sucla2 succinate-Coenzyme A ligase, ADP-forming, beta subunit | 169 | 51865 | 7 |
| IPI00949131 | - 38 kDa protein | 168 | 38972 | 6 |
| IPI00214540 | Cyp4a8 Cytochrome P450 4A12 | 168 | 59769 | 8 |
| IPI00778620 | Cyp4f17 similar to Cytochrome P450 4F6 | 168 | 61542 | 7 |
| IPI00559821 | Cyp4f40 cytochrome P450, family 4, subfamily f, polypeptide 40 | 168 | 60826 | 6 |
| IPI00195673 | Tubb6 Tubulin, beta 6 | 165 | 50715 | 18 |
| IPI00758468 | Pgcp Plasma glutamate carboxypeptidase | 164 | 53084 | 8 |
| IPI00214373 | Aadat Kynurenine/alpha-aminoadipate aminotransferase, mitochondrial | 163 | 48958 | 11 |
| IPI00421885 | Acot2 Acyl-coenzyme A thioesterase 2, mitochondrial | 163 | 50616 | 10 |
| IPI00764023 | Col12a1 similar to procollagen, type XII, alpha 1 | 163 | 345298 | 16 |
| IPI00209480 | Pccb Propionyl-CoA carboxylase beta chain, mitochondrial | 163 | 59578 | 18 |
| IPI00195109 | Shmt2 Serine hydroxymethyltransferase | 163 | 56633 | 10 |
| IPI00194045 | Idh1 Isocitrate dehydrogenase [NADP] cytoplasmic | 162 | 48296 | 11 |
| IPI00198717 | Mdh1 Malate dehydrogenase, cytoplasmic | 162 | 37750 | 12 |
| IPI00231714 | Dlat Dihydrolipoyllysine-residue acetyltransferase component of pyruvate dehydrogenase complex, mitochondrial | 161 | 68844 | 12 |
| IPI00325146 | Anxa2 Isoform Short of Annexin A2 | 160 | 40073 | 12 |
| IPI00327202 | Aqp1 Aquaporin-1 | 159 | 29139 | 6 |
| IPI00231148 | Gpd1 Glycerol-3-phosphate dehydrogenase [NAD+], cytoplasmic | 158 | 38112 | 25 |
| IPI00205135 | Tgm2 Transglutaminase 2, C polypeptide | 158 | 78220 | 17 |
| IPI00464587 | Tuba8 Tubulin alpha-8 chain | 158 | 50823 | 5 |
| IPI00231611 | Fh1 Isoform Mitochondrial of Fumarate hydratase, mitochondrial | 157 | 55848 | 7 |
| IPI00471584 | Hsp90ab1 Heat shock protein HSP 90-beta | 157 | 86455 | 12 |
| IPI00365542 | Lamb1 Protein | 157 | 206523 | 8 |
| IPI00203690 | Aldh9a1 4-trimethylaminobutyraldehyde dehydrogenase | 156 | 57680 | 8 |
| IPI00193894 | - 197 kDa protein | 155 | 200379 | 22 |
| IPI00363395 | Rap1b Ras-related protein Rap-1b | 154 | 21387 | 4 |
| IPI00556932 | - 16 kDa protein | 152 | 17245 | 4 |
| IPI00212015 | Acadm Medium-chain specific acyl-CoA dehydrogenase, mitochondrial | 152 | 47859 | 11 |
| IPI00780961 | Hist1h2bn histone cluster 1, H2bn | 152 | 16387 | 4 |
| IPI00209163 | Hist2h2be similar to histone 2, H2be | 152 | 14815 | 4 |
| IPI00388454 | Hist3h2ba;LOC687018 histone cluster 3, H2ba | 152 | 14803 | 4 |
| IPI00421364 | Shmt1 Serine hydroxymethyltransferase | 150 | 76067 | 20 |
| IPI00211648 | Slc3a1 Neutral and basic amino acid transport protein rBAT | 150 | 80048 | 10 |
| IPI00197344 | Mgll Monoglyceride lipase | 149 | 33994 | 10 |
| IPI00196451 | Hsd11b2 Corticosteroid 11-beta-dehydrogenase isozyme 2 | 148 | 44215 | 7 |
| IPI00212868 | Lamb2 Laminin subunit beta-2 | 148 | 197766 | 13 |
| IPI00421874 | Vdac1 Voltage-dependent anion-selective channel protein 1 | 148 | 31812 | 9 |
| IPI00214158 | Slc22a6 Solute carrier family 22 member 6 | 147 | 61372 | 10 |
| IPI00471889 | Anxa5 Annexin A5 | 146 | 36625 | 5 |
| IPI00213057 | Acadvl Very long-chain specific acyl-CoA dehydrogenase, mitochondrial | 145 | 72468 | 8 |
| IPI00230907 | Mif Macrophage migration inhibitory factor | 144 | 12640 | 9 |
| IPI00214905 | Tpm4 Tropomyosin alpha-4 chain | 144 | 29568 | 5 |
| IPI00569785 | - 30 kDa protein | 143 | 30662 | 6 |
| IPI00361462 | - Uncharacterized protein | 143 | 13457 | 7 |
| IPI00195372 | Eef1a1 Elongation factor 1-alpha 1 | 143 | 52103 | 12 |
| IPI00194324 | Pdhb Pyruvate dehydrogenase E1 component subunit beta, mitochondrial | 143 | 39774 | 4 |
| IPI00564409 | RGD1309537 Myosin regulatory light chain RLC-A | 143 | 20485 | 9 |
| IPI00361677 | RGD1564839 mCG49427-like | 143 | 23965 | 6 |
| IPI00205417 | Slc27a2 Very long-chain acyl-CoA synthetase | 143 | 72412 | 8 |
| IPI00205157 | Hadh Hydroxyacyl-coenzyme A dehydrogenase, mitochondrial | 141 | 35716 | 6 |
| IPI00365985 | Tra1 Isoform 1 of Endoplasmin | 141 | 95896 | 20 |
| IPI00206254 | Ggt1 Gamma-glutamyltranspeptidase 1 | 140 | 62689 | 5 |
| IPI00951175 | Myo6 Protein | 140 | 151082 | 8 |
| IPI00192043 | Mccc2 Methylcrotonoyl-CoA carboxylase beta chain, mitochondrial | 139 | 62511 | 12 |
| IPI00557582 | - 20 kDa protein | 138 | 20668 | 6 |
| IPI00188119 | Atp12a Isoform Long of Potassium-transporting ATPase alpha chain 2 | 138 | 115643 | 16 |
| IPI00372845 | Gstt1 Uncharacterized protein | 138 | 27866 | 12 |
| IPI00210566 | Hsp90aa1 Heat shock protein HSP 90-alpha | 138 | 88202 | 10 |
| IPI00206624 | Hspa5 78 kDa glucose-regulated protein | 138 | 74926 | 10 |
| IPI00362949 | Uqcrfs1 Cytochrome b-c1 complex subunit Rieske, mitochondrial | 138 | 30115 | 11 |
| IPI00205374 | Hmgcl Hydroxymethylglutaryl-CoA lyase, mitochondrial | 137 | 35116 | 3 |
| IPI00764184 | LOC683295 keratin, type II cytoskeletal 6A-like isoform 1 | 137 | 59735 | 10 |
| IPI00959248 | LOC683720 rCG50520-like | 137 | 58869 | 10 |
| IPI00231136 | Nid1 similar to Nidogen-1 precursor | 137 | 138328 | 7 |
| IPI00362927 | Tuba4a Tubulin alpha-4A chain | 136 | 50709 | 6 |
| IPI00214480 | Fah Fumarylacetoacetase | 135 | 46677 | 9 |
| IPI00763209 | LOC684685 regulator of G-protein signaling like 1-like | 135 | 127943 | 21 |
| IPI00395267 | Pck1 Phosphoenolpyruvate carboxykinase, cytosolic [GTP] | 135 | 71177 | 15 |
| IPI00951316 | Psat1 Phosphoserine aminotransferase | 135 | 40699 | 16 |
| IPI00851115 | Pccb Propionyl coenzyme A carboxylase, beta polypeptide | 134 | 59630 | 15 |
| IPI00327144 | Cfl1 Cofilin-1 | 133 | 18749 | 3 |
| IPI00763003 | LOC365050 similar to heat shock protein 1, alpha | 133 | 25407 | 5 |
| IPI00393867 | Myo1c Myosin-Ic | 133 | 122575 | 14 |
| IPI00769286 | Slc5a12 solute carrier family 5 (sodium/glucose cotransporter), member 12 | 133 | 69097 | 5 |
| IPI00369217 | Trap1 Heat shock protein 75 kDa, mitochondrial | 133 | 82389 | 4 |
| IPI00557049 | - 39 kDa protein | 132 | 39685 | 8 |
| IPI00365734 | Aldh16a1 Aldehyde dehydrogenase family 16 member A1 | 132 | 86006 | 9 |
| IPI00555252 | Gapdh;LOC685186;LOC682005 Glyceraldehyde-3-phosphate dehydrogenase | 132 | 36923 | 15 |
| IPI00769218 | RGD1565368 similar to glyceraldehyde-3-phosphate dehydrogenase | 132 | 36878 | 15 |
| IPI00208205 | Hspa8 Heat shock cognate 71 kDa protein | 131 | 73150 | 15 |
| IPI00231767 | Tpi1 Triosephosphate isomerase | 131 | 27735 | 4 |
| IPI00188989 | Acsl1 Long-chain-fatty-acid--CoA ligase 1 | 129 | 80236 | 11 |
| IPI00559851 | - 46 kDa protein | 128 | 47031 | 10 |
| IPI00207941 | Dmgdh Dimethylglycine dehydrogenase, mitochondrial | 128 | 98095 | 14 |
| IPI00566672 | LOC689908 similar to heat shock protein 8 | 128 | 72708 | 7 |
| IPI00411232 | Ccbl1 Isoform 1 of Kynurenine--oxoglutarate transaminase 1, mitochondrial | 127 | 52075 | 7 |
| IPI00193279 | Oat Ornithine aminotransferase, mitochondrial | 126 | 49420 | 14 |
| IPI00776522 | - 31 kDa protein | 125 | 32233 | 10 |
| IPI00564357 | - 37 kDa protein | 125 | 37364 | 8 |
| IPI00765501 | Gnpda1 RCG49489, isoform CRA_a | 125 | 33591 | 4 |
| IPI00949107 | Srprb Cc1-8 | 125 | 110440 | 19 |
| IPI00328013 | Akr7a3 Aflatoxin B1 aldehyde reductase member 3 | 124 | 37411 | 6 |
| IPI00339124 | Atp1b1 Sodium/potassium-transporting ATPase subunit beta-1 | 124 | 36555 | 4 |
| IPI00204316 | Atp5j ATP synthase-coupling factor 6, mitochondrial | 124 | 12487 | 7 |
| IPI00198720 | Idh3a Isocitrate dehydrogenase [NAD] subunit alpha, mitochondrial | 124 | 40663 | 10 |
| IPI00205332 | Etfa Electron transfer flavoprotein subunit alpha, mitochondrial | 123 | 36048 | 3 |
| IPI00206336 | Lamp1 Lysosome-associated membrane glycoprotein 1 | 123 | 44844 | 2 |
| IPI00191385 | Sacm1l Phosphatidylinositide phosphatase SAC1 | 123 | 68415 | 5 |
| IPI00371236 | Tufm Elongation factor Tu, mitochondrial | 123 | 50824 | 5 |
| IPI00210090 | Hnrnpu SP120 | 122 | 88492 | 16 |
| IPI00231260 | Prdx6 Peroxiredoxin-6 | 122 | 25577 | 7 |
| IPI00373168 | Acss1 acyl-CoA synthetase short-chain family member 1 | 121 | 75962 | 9 |
| IPI00558912 | Myh11 152 kDa protein | 121 | 157731 | 14 |
| IPI00372786 | Nid2 Isoform 1 of Nidogen-2 | 121 | 153869 | 9 |
| IPI00949898 | - 71 kDa protein | 120 | 73495 | 12 |
| IPI00763872 | Gstz1 RCG20683, isoform CRA_b | 120 | 24174 | 9 |
| IPI00870183 | Cisd1 CDGSH iron-sulfur domain-containing protein 1 | 119 | 12260 | 8 |
| IPI00464512 | Hnrnph2 Heterogeneous nuclear ribonucleoprotein H2 | 119 | 49547 | 12 |
| IPI00231202 | Rps8 40S ribosomal protein S8 | 119 | 24475 | 5 |
| IPI00362243 | Ak3 GTP:AMP phosphotransferase mitochondrial | 118 | 26111 | 10 |
| IPI00231192 | LOC100134871;LOC689064;Hbb;MGC72973 Hemoglobin subunit beta-2 | 118 | 16488 | 9 |
| IPI00421392 | LOC500959 Triosephosphate isomerase | 118 | 27306 | 12 |
| IPI00952436 | Atp6v1h 56 kDa protein | 117 | 57209 | 4 |
| IPI00231229 | Gstp1 Glutathione S-transferase P | 117 | 23983 | 3 |
| IPI00324741 | Pdia3 Protein disulfide-isomerase A3 | 117 | 58738 | 9 |
| IPI00561052 | RGD156237 Isoform Histone H4 of Histone H4 | 117 | 11833 | 5 |
| IPI00230835 | Ywhag 14-3-3 protein gamma | 117 | 29016 | 8 |
| IPI00365851 | Atp6v1d ATPase, H+ transporting, lysosomal V1 subunit D | 116 | 29452 | 4 |
| IPI00204774 | Cesl1 Liver carboxylesterase B-1 | 116 | 63960 | 9 |
| IPI00370752 | Nit2 Omega-amidase NIT2 | 116 | 31499 | 3 |
| IPI00210032 | Pter Phosphotriesterase-related protein | 116 | 39894 | 7 |
| IPI00213324 | Slc22a1 Isoform 2 of Solute carrier family 22 member 1 | 116 | 48205 | 6 |
| IPI00191929 | Tmem27 Collectrin | 116 | 25465 | 2 |
| IPI00324986 | Gdi1 Rab GDP dissociation inhibitor alpha | 115 | 51751 | 4 |
| IPI00475676 | LOC641316 Delta-1-pyrroline-5-carboxylate dehydrogenase, mitochondrial | 115 | 63077 | 8 |
| IPI00382282 | - Ac2-281 | 114 | 7588 | 7 |
| IPI00208215 | Prdx3 Thioredoxin-dependent peroxide reductase, mitochondrial | 112 | 28837 | 6 |
| IPI00230939 | Rpl24 60S ribosomal protein L24 | 111 | 17882 | 4 |
| IPI00365106 | Atp6v1b1 ATPase, H transporting, lysosomal V1 subunit B1 | 110 | 57675 | 8 |
| IPI00191593 | Trpv4 Transient receptor potential cation channel subfamily V member 4 | 110 | 99839 | 7 |
| IPI00206977 | Cs Citrate synthase, mitochondrial | 109 | 52995 | 18 |
| IPI00231292 | Hrsp12 Ribonuclease UK114 | 109 | 14639 | 4 |
| IPI00230937 | Pebp1 Phosphatidylethanolamine-binding protein 1 | 109 | 21347 | 3 |
| IPI00231609 | Rpl6-ps1 similar to 60S ribosomal protein L6 | 109 | 35675 | 5 |
| IPI00208203 | Eci2 Enoyl-CoA delta isomerase 2, mitochondrial | 108 | 43336 | 12 |
| IPI00191681 | Itgb1 Integrin beta-1 | 108 | 90845 | 4 |
| IPI00207702 | Slc22a8 Solute carrier family 22 member 8 | 108 | 60180 | 8 |
| IPI00192076 | - 100 kDa protein | 107 | 102841 | 9 |
| IPI00201399 | Alad Delta-aminolevulinic acid dehydratase | 107 | 36524 | 3 |
| IPI00411230 | Gstm2 Glutathione S-transferase Mu 2 | 107 | 26546 | 7 |
| IPI00231356 | Mthfd1 C-1-tetrahydrofolate synthase, cytoplasmic | 107 | 103598 | 10 |
| IPI00206664 | Acp1 Isoform 1 of Low molecular weight phosphotyrosine protein phosphatase | 106 | 18596 | 4 |
| IPI00734731 | Mt-nd5 NADH dehydrogenase subunit 5 | 106 | 69604 | 7 |
| IPI00362963 | Eci3 Uncharacterized protein | 105 | 33836 | 8 |
| IPI00202658 | Hibadh 3-hydroxyisobutyrate dehydrogenase, mitochondrial | 105 | 36183 | 7 |
| IPI00370427 | Tinag Tubulointerstitial nephritis antigen | 105 | 55036 | 17 |
| IPI00190428 | Tinagl1 Tubulointerstitial nephritis antigen-like | 105 | 53173 | 4 |
| IPI00189795 | Tuba1a Tubulin alpha-1A chain | 105 | 50921 | 11 |
| IPI00213569 | Ugt1a1 UDP-glucuronosyltransferase 1-1 | 105 | 61000 | 6 |
| IPI00370520 | - 35 kDa protein | 104 | 35952 | 4 |
| IPI00188134 | Fxyd2 Isoform 1 of Sodium/potassium-transporting ATPase subunit gamma | 104 | 7468 | 2 |
| IPI00656375 | RGD1562758 similar to glyceraldehyde-3-phosphate dehydrogenase | 104 | 36952 | 4 |
| IPI00778558 | - Protein | 103 | 116468 | 3 |
| IPI00213684 | Fuca1 Tissue alpha-L-fucosidase | 103 | 53737 | 4 |
| IPI00202570 | Rab2a Ras-related protein Rab-2A | 103 | 24037 | 3 |
| IPI00212651 | Timm13 Mitochondrial import inner membrane translocase subunit Tim13 | 103 | 10709 | 12 |
| IPI00191090 | Bgn Biglycan | 102 | 42712 | 4 |
| IPI00551702 | Dlst Dihydrolipoyllysine-residue succinyltransferase component of 2-oxoglutarate dehydrogenase complex, mitochondrial | 102 | 50013 | 8 |
| IPI00561643 | - 22 kDa protein | 101 | 22361 | 4 |
| IPI00392259 | - 28 kDa protein | 101 | 29053 | 5 |
| IPI00363402 | - 34 kDa protein | 101 | 34995 | 5 |
| IPI00569325 | - 34 kDa protein | 101 | 35075 | 4 |
| IPI00566322 | - 35 kDa protein | 101 | 35948 | 6 |
| IPI00393046 | - 35 kDa protein | 101 | 35971 | 5 |
| IPI00208917 | Aldh7a1 similar to aldehyde dehydrogenase family 7, member A1 | 101 | 59959 | 9 |
| IPI00765735 | LOC291545 similar to Glyceraldehyde-3-phosphate dehydrogenase | 101 | 20100 | 5 |
| IPI00763753 | LOC500912 similar to Glyceraldehyde-3-phosphate dehydrogenase | 101 | 17569 | 10 |
| IPI00559336 | LOC688815 similar to prohibitin | 101 | 24124 | 7 |
| IPI00326195 | Pecr Peroxisomal trans-2-enoyl-CoA reductase | 101 | 33144 | 4 |
| IPI00211733 | Psmc1 26S protease regulatory subunit 4 | 101 | 51089 | 3 |
| IPI00210381 | Rab11b Ras-related protein Rab-11B | 101 | 25033 | 6 |
| IPI00362347 | RGD1559590 similar to glyceraldehyde-3-phosphate dehydrogenase | 101 | 30794 | 11 |
| IPI00373541 | RGD1560797 similar to glyceraldehyde-3-phosphate dehydrogenase | 101 | 46000 | 4 |
| IPI00360237 | RGD1563861 ribosomal protein 10-like | 101 | 25147 | 5 |
| IPI00362452 | RGD1564958 similar to Glyceraldehyde-3-phosphate dehydrogenase | 101 | 37090 | 10 |
| IPI00564500 | RGD1564963 ribosomal protein 10-like | 101 | 16027 | 5 |
| IPI00475946 | Tf Isoform 2 of Serotransferrin | 100 | 56295 | 16 |
| IPI00231423 | C9 C9 protein | 99 | 65411 | 5 |
| IPI00365982 | Ckap4 cytoskeleton-associated protein 4 | 99 | 65186 | 4 |
| IPI00200794 | Dcxr L-xylulose reductase | 99 | 26047 | 7 |
| IPI00829505 | Lap3 Isoform 2 of Cytosol aminopeptidase | 99 | 54102 | 6 |
| IPI00764677 | LOC684556 similar to mitochondrial hepatocellular carcinoma-downregulated carrier protein | 99 | 31995 | 3 |
| IPI00231786 | Me1 NADP-dependent malic enzyme | 99 | 64589 | 16 |
| IPI00231139 | Tkt transketolase | 99 | 73092 | 6 |
| IPI00858395 | Aass Alpha-aminoadipic semialdehyde synthase, mitochondrial | 97 | 105336 | 8 |
| IPI00231756 | Aldh1a7 Aldehyde dehydrogenase, cytosolic 1 | 97 | 56159 | 6 |
| IPI00851133 | Hnrph1 Isoform 3 of Heterogeneous nuclear ribonucleoprotein H | 97 | 40882 | 10 |
| IPI00324585 | Itga1 Integrin alpha-1 | 97 | 133564 | 4 |
| IPI00325312 | Prom1 prominin 1 isoform 1 | 97 | 98806 | 3 |
| IPI00325765 | Akr7a2 Aflatoxin B1 aldehyde reductase member 2 | 96 | 41208 | 10 |
| IPI00193777 | Atp6v0a4 95 kDa protein | 96 | 96782 | 9 |
| IPI00205466 | Cand1 Cullin-associated NEDD8-dissociated protein 1 | 96 | 139629 | 6 |
| IPI00207933 | Dhrs4 dehydrogenase/reductase SDR family member 4 | 96 | 30577 | 9 |
| IPI00201057 | Hk2 Hexokinase-2 | 96 | 104757 | 15 |
| IPI00197555 | Suclg1 Succinyl-CoA ligase [GDP-forming] subunit alpha, mitochondrial | 96 | 36524 | 18 |
| IPI00400615 | Atp6v1e1 V-type proton ATPase subunit E 1 | 95 | 27144 | 7 |
| IPI00389350 | Ganab neutral alpha-glucosidase AB | 95 | 91633 | 6 |
| IPI00231780 | H1f0 Histone H1.0 | 95 | 20872 | 10 |
| IPI00476749 | Mug2 Murinoglobulin-2 | 95 | 165753 | 8 |
| IPI00421490 | Naprt1 Nicotinate phosphoribosyltransferase | 95 | 59130 | 1 |
| IPI00326436 | Oplah 5-oxoprolinase | 95 | 138279 | 6 |
| IPI00325823 | Fmo4 Dimethylaniline monooxygenase [N-oxide-forming] 4 | 94 | 65339 | 7 |
| IPI00369691 | Agphd1 aminoglycoside phosphotransferase domain containing 1 | 93 | 43265 | 2 |
| IPI00231862 | Gclc Glutamate--cysteine ligase catalytic subunit | 93 | 74121 | 7 |
| IPI00213436 | Ndufa4 RCG28086, isoform CRA_a | 93 | 9665 | 2 |
| IPI00765520 | RGD1563375 Small nuclear ribonucleoprotein-associated protein B-like | 93 | 23585 | 6 |
| IPI00367135 | Scly Selenocysteine lyase | 93 | 47682 | 8 |
| IPI00409539 | Flna Uncharacterized protein | 92 | 283853 | 16 |
| IPI00870114 | Gcdh glutaryl-Coenzyme A dehydrogenase | 92 | 50499 | 9 |
| IPI00327079 | Gstk1 Glutathione S-transferase kappa 1 | 92 | 25590 | 7 |
| IPI00556987 | Hgd Homogentisate 1, 2-dioxygenase | 92 | 50666 | 14 |
| IPI00869818 | Sec23a SEC23A | 92 | 87354 | 6 |
| IPI00421899 | Cndp2 Cytosolic non-specific dipeptidase | 91 | 54251 | 9 |
| IPI00760146 | Cyb5r3 Isoform 2 of NADH-cytochrome b5 reductase 3 | 91 | 35617 | 8 |
| IPI00364321 | Etfb Electron transfer flavoprotein subunit beta | 91 | 28788 | 12 |
| IPI00202283 | Psmd13 26S proteasome non-ATPase regulatory subunit 13 | 91 | 43779 | 2 |
| IPI00763440 | - similar to Maltase-glucoamylase, intestinal | 90 | 247250 | 10 |
| IPI00325847 | Cp GPI-anchored ceruloplasmin | 90 | 126638 | 11 |
| IPI00201032 | Hnrpd Isoform 1 of Heterogeneous nuclear ribonucleoprotein D0 | 90 | 38339 | 7 |
| IPI00765011 | LOC295810 similar to Actin, cytoplasmic 2 | 90 | 59624 | 17 |
| IPI00287713 | Si Sucrase-isomaltase, intestinal | 90 | 213442 | 9 |
| IPI00325135 | Ywhae 14-3-3 protein epsilon | 90 | 29930 | 10 |
| IPI00324893 | Ywhaz 14-3-3 protein zeta/delta | 90 | 28614 | 6 |
| IPI00382226 | RGD1306939 Ab2-162 | 89 | 147973 | 5 |
| IPI00557754 | - 19 kDa protein | 88 | 19717 | 6 |
| IPI00777931 | - 29 kDa protein | 88 | 29210 | 5 |
| IPI00231267 | Atp2b1 Isoform A of Plasma membrane calcium-transporting ATPase 1 | 88 | 133084 | 8 |
| IPI00421995 | Clic1 Chloride intracellular channel protein 1 | 88 | 27306 | 7 |
| IPI00231539 | Psma7 Proteasome subunit alpha type | 88 | 28552 | 2 |
| IPI00388786 | - Uncharacterized protein | 87 | 14448 | 4 |
| IPI00363022 | Actn2 actinin alpha 2 | 87 | 106048 | 8 |
| IPI00215377 | Agpat1 1-acylglycerol-3-phosphate O-acyltransferase 1 | 87 | 32151 | 4 |
| IPI00421888 | Anxa6 Annexin A6 | 87 | 78072 | 3 |
| IPI00950965 | Aprt Adenine phosphoribosyltransferase | 87 | 19963 | 3 |
| IPI00361686 | C1qbp Complement component 1 Q subcomponent-binding protein, mitochondrial | 87 | 31320 | 6 |
| IPI00214448 | Crym Mu-crystallin homolog | 87 | 34221 | 2 |
| IPI00565267 | Fmo3 Dimethylaniline monooxygenase [N-oxide-forming] 3 | 87 | 61513 | 12 |
| IPI00192495 | Ncstn Isoform 1 of Nicastrin | 87 | 79469 | 4 |
| IPI00231683 | Pklr Isoform L-type of Pyruvate kinase isozymes R/L | 87 | 59660 | 6 |
| IPI00213610 | Cryl1 Lambda-crystallin homolog | 86 | 36135 | 4 |
| IPI00358524 | Enpp6 Ectonucleotide pyrophosphatase/phosphodiesterase family member 6 | 86 | 51011 | 6 |
| IPI00212666 | Mug1;LOC297568 Isoform 1 of Murinoglobulin-1 | 86 | 169092 | 8 |
| IPI00359981 | Ndufa13 similar to NADH dehydrogenase (ubiquinone) 1 alpha subcomplex, 13 | 86 | 17251 | 8 |
| IPI00215107 | Rpsa 40S ribosomal protein SA | 86 | 32917 | 4 |
| IPI00196725 | Aldh1l1 10-formyltetrahydrofolate dehydrogenase | 85 | 101515 | 6 |
| IPI00231734 | Aldoa Fructose-bisphosphate aldolase A | 85 | 40445 | 5 |
| IPI00195851 | Aldoal1 Fructose-bisphosphate aldolase | 85 | 40585 | 4 |
| IPI00231801 | Cst3 Cystatin-C | 85 | 15655 | 1 |
| IPI00359623 | LOC683474 similar to aldehyde dehydrogenase 8 family, member A1 isoform 2 | 85 | 54631 | 6 |
| IPI00202370 | Maoa Amine oxidase [flavin-containing] A | 85 | 61190 | 3 |
| IPI00196790 | Rab10 Ras-related protein Rab-10 | 85 | 23747 | 7 |
| IPI00199716 | Sec23b 86 kDa protein | 85 | 87590 | 4 |
| IPI00778786 | - 62 kDa protein | 84 | 64414 | 5 |
| IPI00470317 | Eef1g Elongation factor 1-gamma | 84 | 51448 | 5 |
| IPI00200271 | Ehd4 Pincher | 84 | 63192 | 2 |
| IPI00914178 | Fahd2a Fumarylacetoacetate hydrolase domain-containing protein 2 | 84 | 35376 | 6 |
| IPI00231733 | Gnai1 Guanine nucleotide-binding protein G(i) subunit alpha-1 | 84 | 41609 | 4 |
| IPI00766169 | LOC687295 similar to translocase of inner mitochondrial membrane 50 homolog isoform 2 | 84 | 40518 | 4 |
| IPI00213532 | Nat8 Probable N-acetyltransferase CML4 | 84 | 25222 | 4 |
| IPI00231359 | Acads Acetyl-Coenzyme A dehydrogenase, short chain, isoform CRA_a | 83 | 45885 | 8 |
| IPI00198620 | Atp5d ATP synthase subunit delta, mitochondrial | 83 | 17713 | 1 |
| IPI00231978 | Atp5i ATP synthase subunit e, mitochondrial | 83 | 8249 | 8 |
| IPI00372498 | Dak Bifunctional ATP-dependent dihydroxyacetone kinase/FAD-AMP lyase (cyclizing) | 83 | 60825 | 5 |
| IPI00231971 | Fabp3 Fatty acid-binding protein, heart | 83 | 14766 | 5 |
| IPI00393787 | Parva Alpha-parvin | 83 | 43642 | 2 |
| IPI00475835 | Phgdh D-3-phosphoglycerate dehydrogenase | 83 | 57575 | 6 |
| IPI00325599 | Asl Argininosuccinate lyase | 82 | 52506 | 9 |
| IPI00364715 | Auh AU RNA binding protein/enoyl-Coenzyme A hydratase | 82 | 27755 | 5 |
| IPI00393034 | LOC685778 similar to Pyruvate dehydrogenase E1 component alpha subunit, somatic form, mitochondrial precursor (PDHE1-A type I) isoform 2 | 82 | 45172 | 10 |
| IPI00870631 | Np Purine nucleoside phosphorylase | 82 | 32711 | 7 |
| IPI00193258 | Pah Phenylalanine-4-hydroxylase | 82 | 52950 | 9 |
| IPI00765682 | Pcca propionyl-CoA carboxylase alpha chain, mitochondrial | 82 | 81964 | 11 |
| IPI00768086 | Pdha1 RCG36458 | 82 | 44177 | 11 |
| IPI00480820 | Pgrmc1 Membrane-associated progesterone receptor component 1 | 82 | 22101 | 5 |
| IPI00368250 | RGD1561333 similar to 60S ribosomal protein L8 | 82 | 29257 | 5 |
| IPI00199543 | Rpl7 60S ribosomal protein L7 | 82 | 31987 | 2 |
| IPI00215208 | Rpl8 60S ribosomal protein L8 | 82 | 29125 | 5 |
| IPI00760137 | Sord Sorbitol dehydrogenase | 82 | 39328 | 7 |
| IPI00949459 | Susd2 sushi domain containing 2 | 82 | 90603 | 4 |
| IPI00392894 | - 204 kDa protein | 81 | 207032 | 22 |
| IPI00207252 | Asrgl1 L-asparaginase | 81 | 34845 | 19 |
| IPI00326561 | Ech1 Delta(3,5)-Delta(2,4)-dienoyl-CoA isomerase, mitochondrial | 81 | 37052 | 3 |
| IPI00231781 | Hist1h1t Histone H1t | 81 | 21712 | 9 |
| IPI00764164 | LOC687023;Hist3h2bb histone cluster 3, H2bb | 81 | 14760 | 3 |
| IPI00360075 | Lrpprc Leucine-rich PPR motif-containing protein, mitochondrial | 81 | 160940 | 6 |
| IPI00212314 | Msn Moesin | 81 | 70449 | 8 |
| IPI00196794 | Rab14 Ras-related protein Rab-14 | 81 | 24428 | 10 |
| IPI00391519 | - 44 kDa protein | 80 | 45737 | 3 |
| IPI00231615 | Anxa1 Annexin A1 | 80 | 40138 | 4 |
| IPI00231638 | Gsta2;Gsta3;Gsta1 Glutathione S-transferase alpha-1 | 80 | 26580 | 17 |
| IPI00214152 | Hagh Isoform 2 of Hydroxyacylglutathione hydrolase, mitochondrial | 80 | 29824 | 5 |
| IPI00191103 | Ndufa2 NADH dehydrogenase [ubiquinone] 1 alpha subcomplex subunit 2 | 80 | 10952 | 8 |
| IPI00209807 | Sardh Sarcosine dehydrogenase, mitochondrial | 80 | 102537 | 11 |
| IPI00231379 | Slc4a1 band 3 anion transport protein | 80 | 104855 | 8 |
| IPI00200898 | Slc9a3r1 Na(+)/H(+) exchange regulatory cofactor NHE-RF1 | 80 | 40011 | 2 |
| IPI00327338 | Tmlhe Isoform 2 of Trimethyllysine dioxygenase, mitochondrial | 80 | 51830 | 9 |
| IPI00392981 | - 22 kDa protein | 79 | 22365 | 7 |
| IPI00777934 | - Protein | 79 | 65929 | 11 |
| IPI00366218 | Cct2 T-complex protein 1 subunit beta | 79 | 57764 | 24 |
| IPI00366416 | Cyc1 cytochrome c-1 | 79 | 36014 | 11 |
| IPI00372520 | Eef1b2l Uncharacterized protein | 79 | 28123 | 4 |
| IPI00327398 | Enpep Isoform 1 of Glutamyl aminopeptidase | 79 | 110335 | 9 |
| IPI00944224 | Hagh Isoform 1 of Hydroxyacylglutathione hydrolase, mitochondrial | 79 | 34544 | 9 |
| IPI00207355 | Hspa2 Heat shock-related 70 kDa protein 2 | 79 | 71835 | 5 |
| IPI00475707 | Ugt1a1;Ugt1a6 UDP-glucuronosyltransferase 1-6 | 79 | 61486 | 8 |
| IPI00949879 | - 39 kDa protein | 78 | 39782 | 8 |
| IPI00361815 | - Uncharacterized protein | 78 | 32951 | 8 |
| IPI00211510 | Acox1 Isoform 1 of Peroxisomal acyl-coenzyme A oxidase 1 | 78 | 76395 | 6 |
| IPI00231968 | Anxa4 Annexin A4 | 78 | 36774 | 6 |
| IPI00367829 | Cyfip1 cytoplasmic FMR1 interacting protein 1 | 78 | 148260 | 9 |
| IPI00421361 | Decr2;Rab11fip3 LRRGT00043 | 78 | 97223 | 10 |
| IPI00211927 | Lyz2 Lysozyme C-1 | 78 | 17019 | 2 |
| IPI00200640 | Mucdhl Isoform 1 of Mucin and cadherin-like protein | 78 | 92296 | 2 |
| IPI00948244 | Ndufs2 Protein | 78 | 53206 | 14 |
| IPI00565188 | RGD1559960 similar to Sulfotransferase K1 | 78 | 36061 | 6 |
| IPI00564212 | Acot1 36 kDa protein | 77 | 36155 | 5 |
| IPI00326667 | Acot1 Acyl-coenzyme A thioesterase 1 | 77 | 46844 | 5 |
| IPI00471901 | Ap2a2 Adaptor-related protein complex 2, alpha 2 subunit | 77 | 106387 | 2 |
| IPI00197703 | Apoa1 Apolipoprotein A-I | 77 | 31075 | 4 |
| IPI00194550 | Cth Cystathionine gamma-lyase | 77 | 44609 | 2 |
| IPI00365962 | Ndufs7 NADH dehydrogenase (Ubiquinone) Fe-S protein 7, isoform CRA_d | 77 | 24316 | 4 |
| IPI00763620 | RGD1564209 similar to Acyl-CoA dehydrogenase family member 8, mitochondrial precursor | 77 | 45615 | 7 |
| IPI00191354 | Tpm3 33 kDa protein | 77 | 34677 | 5 |
| IPI00331983 | Adh1 Alcohol dehydrogenase 1 | 76 | 40953 | 2 |
| IPI00382211 | Atad1 Ab2-088 | 76 | 63611 | 4 |
| IPI00327630 | Dync1h1 Cytoplasmic dynein 1 heavy chain 1 | 76 | 544646 | 22 |
| IPI00199482 | Gstm4 RCG29014, isoform CRA_a | 76 | 26182 | 5 |
| IPI00208636 | Gstm5 Glutathione S-transferase Mu 5 | 76 | 27428 | 5 |
| IPI00230942 | Gstm7 Glutathione S-transferase Yb-3 | 76 | 26309 | 6 |
| IPI00210872 | Mep1a Meprin A subunit alpha | 76 | 86289 | 4 |
| IPI00365744 | Nomo1 nodal modulator 1 | 76 | 136240 | 12 |
| IPI00203760 | Rab39 RAB39, member RAS oncogene family | 76 | 25550 | 6 |
| IPI00325762 | Rab3a Ras-related protein Rab-3A | 76 | 25513 | 6 |
| IPI00204737 | Rab43 Ras-related protein Rab-43 | 76 | 23731 | 6 |
| IPI00196789 | Rab8a Ras-related protein Rab-8A | 76 | 24513 | 7 |
| IPI00202576 | RGD1565767 Ribosomal protein L15 | 76 | 24401 | 9 |
| IPI00213659 | Decr1 2,4-dienoyl-CoA reductase, mitochondrial | 75 | 37142 | 5 |
| IPI00193716 | Ivd Isovaleryl-CoA dehydrogenase, mitochondrial | 75 | 47395 | 16 |
| IPI00189991 | Mat2a S-adenosylmethionine synthase isoform type-2 | 75 | 44030 | 4 |
| IPI00211779 | Prdx1 Peroxiredoxin-1 | 75 | 22912 | 4 |
| IPI00191437 | - 64 kDa protein | 74 | 64544 | 11 |
| IPI00369493 | Adh6 Alcohol dehydrogenase 6 | 74 | 40784 | 11 |
| IPI00358872 | Amt Aminomethyltransferase | 74 | 44810 | 3 |
| IPI00372388 | Cct3 T-complex protein 1 subunit gamma | 74 | 62114 | 3 |
| IPI00559259 | Celsr3 359 kDa protein | 74 | 360670 | 12 |
| IPI00365149 | Coq9 Ubiquinone biosynthesis protein COQ9, mitochondrial | 74 | 35382 | 2 |
| IPI00950234 | Dhtkd1 102 kDa protein | 74 | 103782 | 9 |
| IPI00198039 | Ethe1 Ethylmalonic encephalopathy 1 | 74 | 27960 | 7 |
| IPI00372191 | Mccc1 Methylcrotonoyl-CoA carboxylase subunit alpha, mitochondrial | 74 | 81086 | 11 |
| IPI00231426 | Pgk1 Phosphoglycerate kinase 1 | 74 | 46273 | 10 |
| IPI00371769 | Pgk2 Phosphoglycerate kinase | 74 | 46917 | 7 |
| IPI00372910 | RGD1560402 similar to Phosphoglycerate kinase 1 | 74 | 44782 | 9 |
| IPI00193919 | Suox Sulfite oxidase, mitochondrial | 74 | 61628 | 7 |
| IPI00212821 | Akr1cl2 1,5-anhydro-D-fructose reductase | 73 | 35510 | 4 |
| IPI00764737 | Aldh8a1 similar to aldehyde dehydrogenase 8 family, member A1 | 73 | 52137 | 8 |
| IPI00480639 | C3 Complement C3 (Fragment) | 73 | 191202 | 7 |
| IPI00366665 | Car12 Membrane-bound carbonic anhydrase 12 | 73 | 40440 | 1 |
| IPI00470254 | Ezr Ezrin | 73 | 71713 | 8 |
| IPI00191913 | Ndufv1 NADH dehydrogenase (Ubiquinone) flavoprotein 1 | 73 | 51817 | 11 |
| IPI00369635 | Rdx Radixin | 73 | 71254 | 5 |
| IPI00359821 | RGD1308461 similar to CG5149-PA | 73 | 47906 | 4 |
| IPI00366293 | Tst Thiosulfate sulfurtransferase | 73 | 33988 | 15 |
| IPI00765366 | Tubb4 tubulin, beta 4 | 73 | 50156 | 27 |
| IPI00869806 | Actr1a Alpha-centractin | 72 | 42701 | 4 |
| IPI00390795 | Atp1a4 ATPase, Na+/K+ transporting, alpha 4 polypeptide | 72 | 116772 | 8 |
| IPI00560978 | LOC291758 similar to leucine aminopeptidase 3 | 72 | 32840 | 2 |
| IPI00768768 | LOC364268 similar to Tu translation elongation factor, mitochondrial | 72 | 18523 | 3 |
| IPI00765328 | LOC688318 RCG33066, isoform CRA_a | 72 | 78025 | 6 |
| IPI00201500 | Rps14 40S ribosomal protein S14 | 72 | 16420 | 4 |
| IPI00231099 | Spp2 Secreted phosphoprotein 24 | 72 | 23413 | 2 |
| IPI00551625 | Ugt1a3;Ugt1a1 UDP-glucuronosyltransferase 1-3 | 72 | 61304 | 6 |
| IPI00768666 | - similar to Ras-related protein Rab-1B | 71 | 32269 | 7 |
| IPI00210351 | Ak1 Adenylate kinase isoenzyme 1 | 71 | 22387 | 5 |
| IPI00213929 | Bcs1l BCS1-like (Yeast), isoform CRA_a | 71 | 47922 | 5 |
| IPI00211812 | Copb1 Coatomer subunit beta | 71 | 109910 | 4 |
| IPI00360340 | Ehd1 EH domain-containing protein 1 | 71 | 62457 | 3 |
| IPI00471868 | Etfdh Electron-transferring-flavoprotein dehydrogenase | 71 | 69927 | 7 |
| IPI00358757 | Hint2 histidine triad nucleotide binding protein 2 | 71 | 17426 | 10 |
| IPI00211507 | Hpd 4-hydroxyphenylpyruvate dioxygenase | 71 | 46331 | 5 |
| IPI00369774 | Mettl7b Methyltransferase-like protein 7B | 71 | 28703 | 4 |
| IPI00382300 | Rab1 Ac2-048 | 71 | 35664 | 7 |
| IPI00876621 | Rab12 ras-related protein Rab-12 | 71 | 28114 | 6 |
| IPI00196795 | Rab15 Ras-related protein Rab-15 | 71 | 24913 | 6 |
| IPI00480673 | Rab1b-ps1;Rab1b Ras-related protein Rab-1B | 71 | 22837 | 7 |
| IPI00198880 | Rab26 20 kDa protein | 71 | 20522 | 8 |
| IPI00781739 | Rab30 23 kDa protein | 71 | 23474 | 7 |
| IPI00192216 | Rab33b RAB33B, member of RAS oncogene family | 71 | 26295 | 6 |
| IPI00371187 | Rab35 Ras-related protein Rab-35 | 71 | 23785 | 6 |
| IPI00209150 | Rab3c Ras-related protein Rab-3C | 71 | 26501 | 6 |
| IPI00213685 | Rab3d GTP-binding protein Rab-3D | 71 | 24791 | 6 |
| IPI00325763 | Rab4a ras-related protein Rab-4A | 71 | 24909 | 10 |
| IPI00199224 | Rab4b Ras-related protein Rab-4B | 71 | 24044 | 14 |
| IPI00763480 | Rab6a ras-related protein Rab-6A | 71 | 24090 | 7 |
| IPI00189690 | Rab8b Ras-related protein Rab-8B | 71 | 24448 | 6 |
| IPI00202111 | Usmg5 Up-regulated during skeletal muscle growth protein 5 | 71 | 6661 | 2 |
| IPI00365481 | Atad3a ATPase family AAA domain-containing protein 3 | 70 | 68395 | 3 |
| IPI00358406 | Ctnna1 Catenin (Cadherin-associated protein), alpha 1, isoform CRA_b | 70 | 103313 | 6 |
| IPI00201891 | Hk3 Hexokinase-3 | 70 | 101136 | 6 |
| IPI00231789 | Mme Neprilysin | 70 | 87977 | 10 |
| IPI00371500 | RGD1304982 Quinone oxidoreductase-like protein 2 | 70 | 38298 | 4 |
| IPI00567976 | RGD1565421 sulfotransferase K2-like | 70 | 32742 | 13 |
| IPI00188059 | Rpn2 Dolichyl-diphosphooligosaccharide--protein glycosyltransferase subunit 2 | 70 | 70325 | 3 |
| IPI00231043 | Slc12a3 Solute carrier family 12 member 3 | 70 | 112376 | 2 |
| IPI00555171 | Tagln2 Transgelin-2 | 70 | 22895 | 2 |
| IPI00950270 | - 34 kDa protein | 69 | 35515 | 7 |
| IPI00565128 | - 35 kDa protein | 69 | 35661 | 7 |
| IPI00212811 | Ctsb Cathepsin B | 69 | 38048 | 2 |
| IPI00205598 | Grpel1 GrpE protein homolog 1, mitochondrial | 69 | 24510 | 6 |
| IPI00190531 | Gsr glutathione reductase | 69 | 47397 | 2 |
| IPI00209148 | Hnrnpm Isoform 1 of Heterogeneous nuclear ribonucleoprotein M | 69 | 74076 | 22 |
| IPI00563431 | LOC499896 Glyceraldehyde 3-phosphate dehydrogenase (Fragment) | 69 | 9493 | 7 |
| IPI00948391 | LOC499896 similar to glyceraldehyde-3-phosphate dehydrogenase | 69 | 34278 | 6 |
| IPI00204128 | RGD1561381 similar to microsomal glutathione S-transferase 3 | 69 | 17054 | 2 |
| IPI00204703 | Serpinh1 Serpin H1 | 69 | 47993 | 7 |
| IPI00199585 | Slc22a5 Solute carrier family 22 member 5 | 69 | 63215 | 3 |
| IPI00213457 | Atp6v1c1 V-type proton ATPase subunit C 1 | 68 | 45507 | 3 |
| IPI00358059 | Dnpep Aspartyl aminopeptidase | 68 | 53124 | 2 |
| IPI00205076 | Lonp1 Lon protease homolog, mitochondrial | 68 | 108393 | 9 |
| IPI00421625 | Mrlc2 Myosin regulatory light chain 12B | 68 | 20471 | 3 |
| IPI00950080 | - 21 kDa protein | 67 | 21579 | 3 |
| IPI00565818 | - 21 kDa protein | 67 | 21737 | 3 |
| IPI00949070 | - 25 kDa protein | 67 | 26144 | 3 |
| IPI00560130 | - 30 kDa protein | 67 | 30602 | 2 |
| IPI00556964 | - 31 kDa protein | 67 | 32076 | 3 |
| IPI00567786 | - Protein | 67 | 38014 | 2 |
| IPI00362229 | Actr1b ARP1 actin-related protein 1 homolog B | 67 | 42369 | 4 |
| IPI00569920 | Actr2 32 kDa protein | 67 | 33072 | 2 |
| IPI00358127 | Actr3b similar to ARP3 actin-related protein 3 homolog B | 67 | 48507 | 3 |
| IPI00230838 | Atp5h ATP synthase subunit d, mitochondrial | 67 | 19526 | 6 |
| IPI00231982 | Fn1 Isoform 2 of Fibronectin | 67 | 265937 | 8 |
| IPI00231475 | H3f3b;LOC100361558;LOC100365096 Histone H3.3 | 67 | 15376 | 13 |
| IPI00365888 | Rab2b RAB2B, member RAS oncogene family | 67 | 24543 | 4 |
| IPI00200069 | Sfxn3 Sideroflexin-3 | 67 | 35927 | 4 |
| IPI00364318 | Aspdh Putative L-aspartate dehydrogenase | 66 | 31326 | 4 |
| IPI00211127 | Ass1 Argininosuccinate synthase | 66 | 47929 | 13 |
| IPI00421711 | Atp5l ATP synthase, H+ transporting, mitochondrial F0 complex, subunit G | 66 | 11453 | 4 |
| IPI00231858 | Gstt2 Glutathione S-transferase theta-2 | 66 | 27938 | 7 |
| IPI00194562 | Hnrnpr Uncharacterized protein | 66 | 71115 | 3 |
| IPI00768849 | LOC683062;LOC679923 similar to voltage-dependent anion channel 1 | 66 | 22783 | 4 |
| IPI00206948 | Retsat All-trans-retinol 13,14-reductase | 66 | 69294 | 5 |
| IPI00361411 | RGD1564338 similar to aminoacylase 1 | 66 | 27696 | 4 |
| IPI00202214 | Rpl19 60S ribosomal protein L19 | 66 | 23565 | 8 |
| IPI00882453 | Slc2a2 Solute carrier family 2, facilitated glucose transporter member 2 | 66 | 57866 | 4 |
| IPI00777730 | - 39 kDa protein | 65 | 40496 | 6 |
| IPI00782078 | - 65 kDa protein | 65 | 67410 | 4 |
| IPI00188359 | Abhd14b Abhydrolase domain-containing protein 14B | 65 | 22819 | 6 |
| IPI00196210 | Ddx3y DEAD (Asp-Glu-Ala-Asp) box polypeptide 3 Y-linked | 65 | 73474 | 8 |
| IPI00211096 | Hsd11b1 Isoform 11-HSD1A of Corticosteroid 11-beta-dehydrogenase isozyme 1 | 65 | 32637 | 4 |
| IPI00471647 | Ndufs2 NADH dehydrogenase [ubiquinone] iron-sulfur protein 2, mitochondrial | 65 | 52927 | 12 |
| IPI00564737 | RGD1562373 Protein | 65 | 31461 | 7 |
| IPI00389611 | Rgn Regucalcin | 65 | 34185 | 4 |
| IPI00464535 | Serbp1 Isoform 2 of Plasminogen activator inhibitor 1 RNA-binding protein | 65 | 43016 | 6 |
| IPI00655323 | Tmem33 transmembrane protein 33 isoform 2 | 65 | 28167 | 3 |
| IPI00209392 | Trpv1 Stretch-inhibitable nonselective channel | 65 | 62096 | 4 |
| IPI00230941 | Vim Vimentin | 65 | 54646 | 6 |
| IPI00231810 | Add1 Isoform 2 of Alpha-adducin | 64 | 71382 | 1 |
| IPI00373418 | Dbt dihydrolipoamide branched chain transacylase E2 | 64 | 54702 | 5 |
| IPI00369618 | Eif4a1 Eukaryotic translation initiation factor 4A1 | 64 | 46985 | 2 |
| IPI00193595 | Eif4a2 Eukaryotic initiation factor 4A-II | 64 | 47406 | 3 |
| IPI00201494 | Erlin2 Erlin-2 | 64 | 39063 | 5 |
| IPI00567177 | Gapdh-ps2 similar to glyceraldehyde-3-phosphate dehydrogenase isoform 2 | 64 | 36918 | 6 |
| IPI00767591 | Grhpr RCG54768, isoform CRA_a | 64 | 36560 | 7 |
| IPI00211075 | Serpina3n Serine protease inhibitor A3N | 64 | 47999 | 2 |
| IPI00188686 | - 25 kDa protein | 63 | 25459 | 3 |
| IPI00362353 | - Uncharacterized protein | 63 | 29467 | 5 |
| IPI00389196 | Acsf3 similar to C50H11.1 | 63 | 74592 | 6 |
| IPI00205068 | Ap1b1 105 kDa protein | 63 | 107713 | 3 |
| IPI00199600 | Atp6v1g1 Uncharacterized protein | 63 | 13816 | 2 |
| IPI00230830 | Eif2s1 Eukaryotic translation initiation factor 2 subunit 1 | 63 | 36989 | 3 |
| IPI00561647 | Hsd17b4 Peroxisomal multifunctional enzyme type 2 | 63 | 81442 | 16 |
| IPI00213546 | Hspa1l Heat shock 70 kDa protein 1-like | 63 | 72741 | 6 |
| IPI00191711 | LOC259246 Major urinary protein | 63 | 21326 | 2 |
| IPI00565067 | LOC680161 Uncharacterized protein | 63 | 29964 | 3 |
| IPI00765414 | LOC683295 similar to keratin complex 2, basic, gene 6a isoform 2 | 63 | 60504 | 4 |
| IPI00393340 | LOC683313 Keratin, type II cytoskeletal 6A | 63 | 60417 | 2 |
| IPI00764736 | LOC684838 ribosomal protein L7a-like | 63 | 29975 | 4 |
| IPI00204365 | Rpn1 Ribophorin1 | 63 | 70079 | 8 |
| IPI00214654 | Rps24 Isoform 1 of 40S ribosomal protein S24 | 63 | 15413 | 5 |
| IPI00324019 | Serpina1 Alpha-1-antiproteinase | 63 | 47225 | 2 |
| IPI00189536 | Slc5a8 similar to solute carrier family 5 (iodide transporter), member 8 | 63 | 67110 | 3 |
| IPI00391783 | Slc6a19 57 kDa protein | 63 | 57324 | 1 |
| IPI00231737 | Akr1b1 Aldose reductase | 62 | 36849 | 4 |
| IPI00193659 | Akr1b1-ps2 similar to Aldose reductase | 62 | 36953 | 3 |
| IPI00339188 | Haao 3-hydroxyanthranilate 3,4-dioxygenase | 62 | 33120 | 8 |
| IPI00196751 | Hspa1b;Hspa1a Heat shock 70 kDa protein 1A/1B | 62 | 72163 | 6 |
| IPI00768167 | LOC679312 similar to beta tubulin 1, class VI | 62 | 50857 | 8 |
| IPI00464503 | MGC94335 Uncharacterized protein C2orf47 homolog, mitochondrial | 62 | 33714 | 4 |
| IPI00394409 | Pmpca mitochondrial-processing peptidase subunit alpha precursor | 62 | 59064 | 2 |
| IPI00359978 | Rps28 40S ribosomal protein S28 | 62 | 7893 | 2 |
| IPI00189989 | Spr Sepiapterin reductase | 62 | 28412 | 3 |
| IPI00421723 | Tbca Tubulin-specific chaperone A | 62 | 12793 | 5 |
| IPI00371141 | Xylb Xylulose kinase | 62 | 60531 | 2 |
| IPI00367441 | - 50 kDa protein | 61 | 51637 | 2 |
| IPI00230874 | Blvra Biliverdin reductase A | 61 | 34405 | 3 |
| IPI00191728 | Calr Calreticulin | 61 | 49772 | 2 |
| IPI00869481 | Cotl1 Coactosin-like protein | 61 | 16438 | 2 |
| IPI00364311 | Gpi Glucose-6-phosphate isomerase | 61 | 64550 | 5 |
| IPI00767531 | LOC682465;LOC680217 similar to Ferritin light chain 2 | 61 | 15339 | 2 |
| IPI00766218 | LOC683536 ubiquitin-conjugating enzyme E2 N-like | 61 | 17200 | 3 |
| IPI00559098 | Rps19 40S ribosomal protein S19 | 61 | 16076 | 6 |
| IPI00204831 | Timm8a1 Mitochondrial import inner membrane translocase subunit Tim8 A | 61 | 11263 | 3 |
| IPI00231694 | Xdh Xanthine dehydrogenase/oxidase | 61 | 149632 | 5 |
| IPI00951899 | - 81 kDa protein | 60 | 83446 | 10 |
| IPI00200773 | Actn3 Skeletal muscle-specific alpha-actinin 3 | 60 | 104969 | 6 |
| IPI00204118 | Aifm1 Apoptosis-inducing factor 1, mitochondrial | 60 | 68358 | 10 |
| IPI00231651 | Basp1 Uncharacterized protein | 60 | 21804 | 7 |
| IPI00370815 | Cct8 chaperonin containing Tcp1, subunit 8 | 60 | 61400 | 5 |
| IPI00557186 | Cndp2 44 kDa protein | 60 | 45410 | 3 |
| IPI00192246 | Cox5a Cytochrome c oxidase subunit 5A, mitochondrial | 60 | 16420 | 5 |
| IPI00202971 | Dhrs1 Dehydrogenase/reductase (SDR family) member 1 | 60 | 34681 | 3 |
| IPI00357893 | Hibch Isoform 2 of 3-hydroxyisobutyryl-CoA hydrolase, mitochondrial | 60 | 35363 | 3 |
| IPI00366324 | Hist1h1a Uncharacterized protein | 60 | 21991 | 3 |
| IPI00206298 | Lgmn Legumain | 60 | 50553 | 3 |
| IPI00364134 | Nipsnap1 4-nitrophenylphosphatase domain and non-neuronal SNAP25-like protein homolog 1 | 60 | 34185 | 9 |
| IPI00767638 | RGD1562373 similar to 3-ketoacyl-CoA thiolase B, peroxisomal precursor | 60 | 44578 | 8 |
| IPI00210238 | Rps23 40S ribosomal protein S23 | 60 | 15969 | 5 |
| IPI00204469 | Sult1c2a Sulfotransferase 1C2A | 60 | 35782 | 2 |
| IPI00367011 | - 20 kDa protein | 59 | 20552 | 1 |
| IPI00201953 | Acmsd 2-amino-3-carboxymuconate-6-semialdehyde decarboxylase | 59 | 39012 | 2 |
| IPI00327143 | Alpl Alkaline phosphatase, tissue-nonspecific isozyme | 59 | 58913 | 5 |
| IPI00471800 | Cdh16 Cadherin 16 | 59 | 90794 | 3 |
| IPI00231013 | Cyb5a Isoform Short of Cytochrome b5 | 59 | 11830 | 5 |
| IPI00366735 | Gpd1l similar to glycerol-3-phosphate dehydrogenase 1-like | 59 | 39246 | 3 |
| IPI00212969 | Hnrnpa2b1 Isoform B0b of Heterogeneous nuclear ribonucleoproteins A2/B1 | 59 | 34755 | 1 |
| IPI00210357 | Hnrnpf Heterogeneous nuclear ribonucleoprotein F | 59 | 46043 | 8 |
| IPI00212697 | Napsa napsin A aspartic peptidase | 59 | 46117 | 2 |
| IPI00869516 | Ogdhl oxoglutarate dehydrogenase-like | 59 | 118745 | 11 |
| IPI00363182 | RGD1560896 similar to solute carrier family 25, member 5 | 59 | 34187 | 12 |
| IPI00231474 | Rps15a 40S ribosomal protein S15a | 59 | 14944 | 5 |
| IPI00371684 | Sfxn2 Sideroflexin 2 | 59 | 36793 | 2 |
| IPI00205413 | Slc25a20 Mitochondrial carnitine/acylcarnitine carrier protein | 59 | 33906 | 8 |
| IPI00231927 | Slc25a4 ADP/ATP translocase 1 | 59 | 34000 | 11 |
| IPI00200466 | Slc25a5 ADP/ATP translocase 2 | 59 | 33912 | 14 |
| IPI00191216 | Tardbp Uncharacterized protein | 59 | 44888 | 4 |
| IPI00367666 | - 34 kDa protein | 58 | 35370 | 7 |
| IPI00188858 | Amacr Alpha-methylacyl-CoA racemase | 58 | 42576 | 6 |
| IPI00213618 | Atp2a1 Sarcoplasmic/endoplasmic reticulum calcium ATPase 1 | 58 | 111618 | 7 |
| IPI00190020 | Atp2a2 Isoform SERCA2B of Sarcoplasmic/endoplasmic reticulum calcium ATPase 2 | 58 | 117321 | 7 |
| IPI00193918 | Cox5b Cytochrome c oxidase subunit 5B, mitochondrial | 58 | 14191 | 5 |
| IPI00845876 | Fga Fga protein | 58 | 62145 | 9 |
| IPI00231925 | Gnai2 Guanine nucleotide-binding protein G(i) subunit alpha-2 | 58 | 41634 | 11 |
| IPI00193277 | Hsd17b11 Estradiol 17-beta-dehydrogenase 11 | 58 | 33906 | 1 |
| IPI00766037 | LOC684314 Glycerate kinase | 58 | 55884 | 2 |
| IPI00870820 | Myl6b;Myl6 myosin, light chain 6B, alkali, smooth muscle and non-muscle | 58 | 12907 | 6 |
| IPI00195160 | Psap Sulfated glycoprotein 1 | 58 | 62908 | 4 |
| IPI00208026 | Selenbp1 Selenium-binding protein 1 | 58 | 53746 | 5 |
| IPI00189762 | Thnsl2 Threonine synthase-like 2 | 58 | 55048 | 7 |
| IPI00781028 | Atad3a 57 kDa protein | 57 | 58845 | 5 |
| IPI00210280 | Comt Isoform 1 of Catechol O-methyltransferase | 57 | 30180 | 3 |
| IPI00199861 | Dcn Decorin | 57 | 40941 | 3 |
| IPI00870112 | Dpysl2 Dihydropyrimidinase-related protein 2 | 57 | 63615 | 3 |
| IPI00655249 | Echdc3 Enoyl-CoA hydratase domain-containing protein 3, mitochondrial | 57 | 32924 | 2 |
| IPI00191738 | Gclm Glutamate--cysteine ligase regulatory subunit | 57 | 31260 | 2 |
| IPI00212478 | Hbe1 RCG39817, isoform CRA_a | 57 | 16611 | 13 |
| IPI00766808 | LOC679899 ribosomal protein S20-like | 57 | 18581 | 6 |
| IPI00189759 | Ndufa10l1 NADH dehydrogenase (ubiquinone) 1 alpha subcomplex 10-like | 57 | 41508 | 4 |
| IPI00558582 | RGD1564865 20-alpha-hydroxysteroid dehydrogenase-like | 57 | 37354 | 7 |
| IPI00230917 | Rpl18 60S ribosomal protein L18 | 57 | 22505 | 3 |
| IPI00421451 | Rps16 40S ribosomal protein S16 | 57 | 16549 | 3 |
| IPI00762996 | Sqrdl Sulfide quinone reductase-like (Yeast), isoform CRA_a | 57 | 51546 | 3 |
| IPI00779050 | - 24 kDa protein | 56 | 24110 | 3 |
| IPI00361239 | - Uncharacterized protein | 56 | 50009 | 6 |
| IPI00951517 | Agrn 174 kDa protein | 56 | 175520 | 5 |
| IPI00188885 | Agrn Isoform 1 of Agrin | 56 | 210864 | 5 |
| IPI00193425 | Bsg Isoform 2 of Basigin | 56 | 30212 | 3 |
| IPI00187662 | Cyfip1 145 kDa protein | 56 | 147807 | 16 |
| IPI00326225 | Dao D-amino-acid oxidase | 56 | 39355 | 4 |
| IPI00325144 | Ddx39a ATP-dependent RNA helicase DDX39A | 56 | 49591 | 16 |
| IPI00454401 | Dhrs7 Down-regulated in nephrectomized rat kidney #3 | 56 | 37023 | 3 |
| IPI00325281 | Eef1a2 Elongation factor 1-alpha 2 | 56 | 52401 | 4 |
| IPI00232011 | Ehhadh Peroxisomal bifunctional enzyme | 56 | 80673 | 6 |
| IPI00199865 | Farsa Phenylalanyl-tRNA synthetase alpha chain | 56 | 58974 | 3 |
| IPI00559163 | Gldc 114 kDa protein | 56 | 115466 | 9 |
| IPI00950332 | Grhpr 36 kDa protein | 56 | 36889 | 5 |
| IPI00778425 | Gstm6l glutathione S-transferase M6-like | 56 | 26170 | 3 |
| IPI00372559 | Hsd17b8 Estradiol 17-beta-dehydrogenase 8 | 56 | 27161 | 4 |
| IPI00231783 | Ldhb L-lactate dehydrogenase B chain | 56 | 37664 | 13 |
| IPI00464886 | LOC652956 p55 protein | 56 | 51747 | 4 |
| IPI00766968 | LOC690226 similar to dehydrogenase/reductase (SDR family) member 7 | 56 | 36827 | 4 |
| IPI00370745 | Mtx2 Metaxin 2 | 56 | 30344 | 3 |
| IPI00767676 | Myh11 myosin-11 | 56 | 236197 | 12 |
| IPI00193397 | Slc34a1 Sodium-dependent phosphate transport protein 2A | 56 | 69523 | 2 |
| IPI00562923 | Slc4a10 Isoform 4 of Sodium-driven chloride bicarbonate exchanger | 56 | 124901 | 5 |
| IPI00679235 | Slc4a4 Isoform 2 of Electrogenic sodium bicarbonate cotransporter 1 | 56 | 118977 | 9 |
| IPI00421289 | Slc4a8 Isoform 2 of Electroneutral sodium bicarbonate exchanger 1 | 56 | 122097 | 4 |
| IPI00365286 | Vcl Vinculin | 56 | 119768 | 11 |
| IPI00231677 | Ywhah 14-3-3 protein eta | 56 | 29054 | 5 |
| IPI00358905 | - 57 kDa protein | 55 | 58744 | 4 |
| IPI00372228 | - 78 kDa protein | 55 | 80590 | 4 |
| IPI00554106 | Acaa1 Isoform 2 of 3-ketoacyl-CoA thiolase A, peroxisomal | 55 | 38740 | 6 |
| IPI00231736 | Aldoc Fructose-bisphosphate aldolase C | 55 | 40162 | 8 |
| IPI00361738 | Apool 28 kDa protein | 55 | 29337 | 4 |
| IPI00557879 | Echdc2 enoyl Coenzyme A hydratase domain containing 2 | 55 | 32060 | 5 |
| IPI00768104 | Eea1 early endosome antigen 1 | 55 | 168269 | 6 |
| IPI00200360 | G6pc Glucose-6-phosphatase | 55 | 41217 | 3 |
| IPI00199465 | Gls Glutaminase kidney isoform, mitochondrial | 55 | 75782 | 1 |
| IPI00210975 | Hyou1 Hypoxia up-regulated protein 1 | 55 | 114532 | 9 |
| IPI00454403 | Isoc1 Isochorismatase domain-containing protein 1 | 55 | 32584 | 2 |
| IPI00566640 | LOC685520 high mobility group box 1 | 55 | 25043 | 8 |
| IPI00207766 | Mgst3 similar to microsomal glutathione S-transferase 3 | 55 | 24839 | 3 |
| IPI00204504 | Plvap Plasmalemma vesicle-associated protein | 55 | 51077 | 4 |
| IPI00365423 | Ppp2r1a Uncharacterized protein | 55 | 66079 | 6 |
| IPI00777683 | RGD1566085 similar to pyridoxal (pyridoxine, vitamin B6) kinase | 55 | 35707 | 9 |
| IPI00364884 | Ssr1 Translocon-associated protein subunit alpha | 55 | 35994 | 1 |
| IPI00213547 | Surf1;Surf4 Ab1-205 | 55 | 84542 | 4 |
| IPI00195803 | Ugdh UDP-glucose 6-dehydrogenase | 55 | 56362 | 6 |
| IPI00778948 | - 161 kDa protein | 54 | 164597 | 5 |
| IPI00559057 | - 34 kDa protein | 54 | 34966 | 2 |
| IPI00563746 | - 34 kDa protein | 54 | 35142 | 2 |
| IPI00189773 | Acadsb Short/branched chain specific acyl-CoA dehydrogenase, mitochondrial | 54 | 49083 | 3 |
| IPI00203054 | Acsf2 Acyl-CoA synthetase family member 2, mitochondrial | 54 | 69348 | 3 |
| IPI00209082 | Actn1 Alpha-actinin-1 | 54 | 105175 | 6 |
| IPI00390086 | Atp5j2 similar to ATP synthase, H+ transporting, mitochondrial F0 complex, subunit f, isoform 2 | 54 | 10833 | 3 |
| IPI00365380 | Col4a2 similar to Collagen alpha-2(IV) chain precursor | 54 | 171015 | 5 |
| IPI00358470 | Golt1b Golt1b protein | 54 | 15641 | 1 |
| IPI00370776 | LOC303448 Similar to glyceraldehyde-3-phosphate dehydrogenase | 54 | 37250 | 2 |
| IPI00209916 | LOC362855 tRNA-splicing ligase RtcB homolog | 54 | 55727 | 15 |
| IPI00767397 | LOC680385 Uncharacterized protein | 54 | 46655 | 5 |
| IPI00364124 | Lrpap1 Alpha-2-macroglobulin receptor-associated protein | 54 | 43640 | 4 |
| IPI00192639 | Opa1 Isoform 3 of Dynamin-like 120 kDa protein, mitochondrial | 54 | 119144 | 8 |
| IPI00366425 | Rabl4 RAB, member of RAS oncogene family-like 4 | 54 | 21342 | 1 |
| IPI00365587 | RGD1560655 similar to hydroxyacyl-Coenzyme A dehydrogenase/3-ketoacyl-Coenzyme A thiolase/enoyl-Coenzyme A hydratase (trifunctional protein), alpha subunit | 54 | 27464 | 5 |
| IPI00390829 | RGD1563145 similar to 60S ribosomal protein L13 | 54 | 25139 | 2 |
| IPI00559898 | RGD1564688 similar to glyceraldehyde-3-phosphate dehydrogenase | 54 | 37110 | 4 |
| IPI00230916 | Rpl13 60S ribosomal protein L13 | 54 | 25370 | 2 |
| IPI00203523 | Rpl23a 60S ribosomal protein L23a | 54 | 17684 | 2 |
| IPI00869568 | Vps35 maternal embryonic message 3 | 54 | 93604 | 4 |
| IPI00394415 | - 35 kDa protein | 53 | 35917 | 4 |
| IPI00557975 | Acad11 Acyl-CoA dehydrogenase family member 11 | 53 | 88993 | 4 |
| IPI00207668 | Afm Afamin | 53 | 71268 | 2 |
| IPI00369349 | Atp6v1e2 Uncharacterized protein | 53 | 26658 | 9 |
| IPI00365663 | Bckdha 2-oxoisovalerate dehydrogenase subunit alpha, mitochondrial | 53 | 51324 | 6 |
| IPI00568450 | Clybl Isoform 1 of Citrate lyase subunit beta-like protein, mitochondrial | 53 | 38416 | 2 |
| IPI00470246 | Ddc Aromatic L-amino acid decarboxylase | 53 | 55140 | 3 |
| IPI00207184 | Erp29 Endoplasmic reticulum resident protein 29 | 53 | 28614 | 4 |
| IPI00373752 | Flnb Uncharacterized protein | 53 | 280122 | 21 |
| IPI00358175 | Flnc Uncharacterized protein | 53 | 293427 | 18 |
| IPI00199641 | Gcs1 Mannosyl-oligosaccharide glucosidase | 53 | 92675 | 4 |
| IPI00231976 | Hist1h2ba Histone H2B type 1-A | 53 | 14273 | 41 |
| IPI00471526 | LOC298795 Similar to 14-3-3 protein sigma | 53 | 28673 | 8 |
| IPI00555262 | Park7 Park7 protein | 53 | 23220 | 8 |
| IPI00367843 | Peg12 paternally expressed 12 | 53 | 29500 | 5 |
| IPI00201561 | Prdx2 Peroxiredoxin-2 | 53 | 22329 | 3 |
| IPI00197369 | Ptges2 prostaglandin E synthase 2 | 53 | 44327 | 4 |
| IPI00193584 | Rnpep Aminopeptidase B | 53 | 73949 | 2 |
| IPI00475474 | Rps4x;LOC100362640;LOC100364620 40S ribosomal protein S4, X isoform | 53 | 29807 | 9 |
| IPI00562127 | Rps4y2 Uncharacterized protein | 53 | 29408 | 8 |
| IPI00187628 | Sptb Erythroid spectrin beta | 53 | 274798 | 14 |
| IPI00760126 | Ywhab Isoform Short of 14-3-3 protein beta/alpha | 53 | 28665 | 8 |
| IPI00188142 | Acy3 Aspartoacylase-2 | 52 | 35784 | 3 |
| IPI00231502 | Ap2b1 Isoform 2 of AP-2 complex subunit beta | 52 | 108248 | 2 |
| IPI00192078 | Bphl Biphenyl hydrolase-like | 52 | 33619 | 9 |
| IPI00470301 | Cct5 T-complex protein 1 subunit epsilon | 52 | 61219 | 2 |
| IPI00194222 | Cox4i1 Cytochrome c oxidase subunit 4 isoform 1, mitochondrial | 52 | 20233 | 11 |
| IPI00471645 | Ddost Dolichyl-diphosphooligosaccharide--protein glycosyltransferase 48 kDa subunit | 52 | 49682 | 4 |
| IPI00190555 | Folh1 Glutamate carboxypeptidase 2 | 52 | 86508 | 4 |
| IPI00231726 | Gnai3 Guanine nucleotide-binding protein G(k) subunit alpha | 52 | 41700 | 8 |
| IPI00192301 | Gpx1 Glutathione peroxidase 1 | 52 | 22631 | 4 |
| IPI00464518 | Hexb Uncharacterized protein | 52 | 61948 | 5 |
| IPI00372689 | Itga3 117 kDa protein | 52 | 118488 | 7 |
| IPI00421857 | Krt1 Keratin, type II cytoskeletal 1 | 52 | 66081 | 5 |
| IPI00392216 | LOC299282 Liver regeneration protein lrryan | 52 | 69858 | 5 |
| IPI00369209 | LOC303140 similar to Organic cation/carnitine transporter 3 | 52 | 60526 | 3 |
| IPI00767651 | LOC689114 similar to NADH-ubiquinone oxidoreductase 24 kDa subunit, mitochondrial precursor | 52 | 12021 | 1 |
| IPI00203766 | Msra Peptide methionine sulfoxide reductase | 52 | 26437 | 1 |
| IPI00211813 | Myh10 Nonmuscle myosin heavy chain B | 52 | 241061 | 23 |
| IPI00358115 | Myt1 117 kDa protein | 52 | 120376 | 2 |
| IPI00189925 | Napa Alpha-soluble NSF attachment protein | 52 | 34289 | 5 |
| IPI00194958 | Picalm Isoform 2 of Phosphatidylinositol-binding clathrin assembly protein | 52 | 66293 | 2 |
| IPI00191501 | Psma6 Proteasome subunit alpha type-6 | 52 | 27838 | 5 |
| IPI00368614 | Rab25 RAB25, member RAS oncogene family | 52 | 23860 | 3 |
| IPI00952027 | RGD1563815 RGD1563815 protein | 52 | 70789 | 6 |
| IPI00212933 | Slc5a1 Sodium/glucose cotransporter 1 | 52 | 74265 | 6 |
| IPI00360564 | Slc5a4b similar to solute carrier family 5 (low affinity glucose cotransporter), member 4 | 52 | 73115 | 3 |
| IPI00204467 | Sult1c2 Sulfotransferase 1C2 | 52 | 34961 | 24 |
| IPI00212014 | Vcp Transitional endoplasmic reticulum ATPase | 52 | 91314 | 4 |
| IPI00192310 | Bcam Basal cell adhesion molecule | 51 | 68243 | 2 |
| IPI00778179 | Dock5 213 kDa protein | 51 | 218760 | 15 |
| IPI00209038 | Fbp2 Fructose-1,6-bisphosphatase isozyme 2 | 51 | 37853 | 7 |
| IPI00231880 | H2afz Histone H2A.Z | 51 | 14147 | 11 |
| IPI00363719 | Hnrpdl Heterogeneous nuclear ribonucleoprotein D-like | 51 | 35500 | 7 |
| IPI00205036 | LOC360504 hemoglobin alpha 2 chain | 51 | 15791 | 6 |
| IPI00559107 | LOC682649 similar to Histone H2A type 1 | 51 | 19282 | 11 |
| IPI00655321 | LOC687575 Similar to Splicing factor U2AF 35 kDa subunit | 51 | 28228 | 2 |
| IPI00231200 | Por NADPH--cytochrome P450 reductase | 51 | 78548 | 3 |
| IPI00388209 | Prkcsh Protein kinase C substrate 80K-H (Predicted), isoform CRA_b | 51 | 60557 | 2 |
| IPI00191761 | Rab5c Rab5c protein | 51 | 23970 | 6 |
| IPI00567379 | RGD1308874 Isoform 1 of Adipocyte plasma membrane-associated protein | 51 | 42810 | 4 |
| IPI00554326 | Slc22a12 Solute carrier family 22 member 12 | 51 | 60507 | 1 |
| IPI00215134 | Snap91 Isoform Long of Clathrin coat assembly protein AP180 | 51 | 95095 | 2 |
| IPI00231643 | Sod1 Superoxide dismutase [Cu-Zn] | 51 | 16289 | 2 |
| IPI00454559 | Txnrd1 Txnrd1 protein | 51 | 64898 | 4 |
| IPI00214601 | Zufsp Zinc finger with UFM1-specific peptidase domain protein | 51 | 67271 | 2 |
| IPI00557688 | - 28 kDa protein | 50 | 28522 | 5 |
| IPI00776957 | - 32 kDa protein | 50 | 32690 | 31 |
| IPI00364363 | - 38 kDa protein | 50 | 38511 | 5 |
| IPI00562360 | - 55 kDa protein | 50 | 56066 | 3 |
| IPI00949416 | - 6 kDa protein | 50 | 6635 | 4 |
| IPI00779851 | - Protein | 50 | 26193 | 6 |
| IPI00778133 | Agxt2 60 kDa protein | 50 | 61217 | 4 |
| IPI00195241 | Apoh Beta-2-glycoprotein 1 | 50 | 34336 | 2 |
| IPI00205749 | Ces1e Uncharacterized protein | 50 | 61986 | 6 |
| IPI00394419 | Chp Calcium-binding protein p22 | 50 | 22418 | 2 |
| IPI00560847 | Dab2 Protein | 50 | 82830 | 7 |
| IPI00200067 | F11r Junctional adhesion molecule A | 50 | 33037 | 1 |
| IPI00190943 | Gfap Isoform 1 of Glial fibrillary acidic protein | 50 | 50787 | 4 |
| IPI00210211 | Gfap Isoform 2 of Glial fibrillary acidic protein | 50 | 50234 | 4 |
| IPI00188304 | Glo1 Lactoylglutathione lyase | 50 | 21537 | 3 |
| IPI00421513 | Got1 Aspartate aminotransferase, cytoplasmic | 50 | 47303 | 8 |
| IPI00200498 | Hdac6 Uncharacterized protein | 50 | 127566 | 11 |
| IPI00207038 | Kb15 Type II keratin Kb15 | 50 | 59436 | 2 |
| IPI00421788 | Krt7 Keratin, type II cytoskeletal 7 | 50 | 51839 | 2 |
| IPI00421780 | Krt73 Keratin, type II cytoskeletal 73 | 50 | 61812 | 4 |
| IPI00337227 | Nit1 nitrilase homolog 1 isoform a | 50 | 36414 | 6 |
| IPI00764604 | RGD1561232 similar to Keratin, type II cytoskeletal 8 | 50 | 59055 | 2 |
| IPI00390733 | RGD1562178 similar to adenylate kinase 2 isoform b | 50 | 26602 | 10 |
| IPI00368950 | RGD1566136 Uncharacterized protein | 50 | 22748 | 8 |
| IPI00551718 | RT1-A2 RT1 class Ia, locus A2 | 50 | 41754 | 3 |
| IPI00551774 | RT1-EC2 Mature alpha chain of major histocompatibility complex class I antigen (Fragment) | 50 | 39627 | 3 |
| IPI00370878 | Scand3 Uncharacterized protein | 50 | 84951 | 13 |
| IPI00187967 | Tmem205 transmembrane protein 205 | 50 | 21429 | 2 |
| IPI00366247 | Ttc35 Tetratricopeptide repeat protein 35 | 50 | 35750 | 8 |
| IPI00368347 | Uba1 Ubiquitin-like modifier-activating enzyme 1 | 50 | 119992 | 2 |
| IPI00324565 | Ucp2 Mitochondrial uncoupling protein 2 | 50 | 33957 | 3 |
| IPI00777829 | - 54 kDa protein | 49 | 54584 | 8 |
| IPI00214801 | Acly Isoform 2 of ATP-citrate synthase | 49 | 122810 | 16 |
| IPI00363828 | Actr3 Uncharacterized protein | 49 | 47734 | 10 |
| IPI00365297 | Agmat Agmatinase, mitochondrial | 49 | 38264 | 4 |
| IPI00567919 | Ap2a1 105 kDa protein | 49 | 107750 | 4 |
| IPI00869978 | Bdh2 3-hydroxybutyrate dehydrogenase, type 2 | 49 | 28348 | 3 |
| IPI00189554 | Cdipt CDP-diacylglycerol--inositol 3-phosphatidyltransferase | 49 | 23812 | 4 |
| IPI00197443 | Chchd2 Coiled-coil-helix-coiled-coil-helix domain containing 2 | 49 | 16000 | 2 |
| IPI00208249 | Clic4 Chloride intracellular channel protein 4 | 49 | 29647 | 6 |
| IPI00421517 | Des Desmin | 49 | 54327 | 4 |
| IPI00230946 | Dpp7 Dipeptidyl peptidase 2 | 49 | 55638 | 1 |
| IPI00368708 | Fahd1 Fumarylacetoacetate hydrolase domain-containing protein 1 | 49 | 25196 | 2 |
| IPI00211074 | LOC299282 Serine protease inhibitor 2.1 (Fragment) | 49 | 25020 | 2 |
| IPI00959871 | LOC691065 hCG1994130-like | 49 | 15613 | 2 |
| IPI00206749 | Ppp2r2b Isoform 2 of Serine/threonine-protein phosphatase 2A 55 kDa regulatory subunit B beta isoform | 49 | 53103 | 8 |
| IPI00781411 | Ppp2r2c 48 kDa protein | 49 | 49394 | 8 |
| IPI00208209 | Prdx4 Peroxiredoxin-4 | 49 | 31676 | 3 |
| IPI00876581 | Rbp4 Retinol-binding protein 4 | 49 | 23592 | 5 |
| IPI00339125 | Slc1a1 Excitatory amino acid transporter 3 | 49 | 57896 | 2 |
| IPI00369654 | Slc25a35 Solute carrier family 25, member 35 | 49 | 32987 | 6 |
| IPI00914293 | Tmem14c transmembrane protein 14C | 49 | 11822 | 3 |
| IPI00471523 | Tuba3a;Tuba3b Tubulin alpha-3 chain | 49 | 50745 | 7 |
| IPI00196783 | - 18 kDa protein | 48 | 19443 | 5 |
| IPI00763657 | - similar to Putative deoxyribose-phosphate aldolase | 48 | 35919 | 2 |
| IPI00421457 | Abcc4 ATP-binding cassette protein C4 | 48 | 151893 | 6 |
| IPI00191444 | Capzb 31 kDa protein | 48 | 32186 | 4 |
| IPI00869949 | Chchd3 coiled-coil-helix-coiled-coil-helix domain containing 3 | 48 | 27192 | 5 |
| IPI00464794 | Cryz Quinone oxidoreductase | 48 | 35899 | 7 |
| IPI00870706 | Dnajc13 DnaJ (Hsp40) homolog, subfamily C, member 13 | 48 | 212040 | 5 |
| IPI00190759 | Fgg Isoform Gamma-B of Fibrinogen gamma chain | 48 | 51228 | 2 |
| IPI00360056 | Galm Aldose 1-epimerase | 48 | 38643 | 2 |
| IPI00372665 | Haus4 Similar to chromosome 14 open reading frame 94 | 48 | 43612 | 4 |
| IPI00360221 | LOC364802 similar to CG8043-PA | 48 | 28911 | 3 |
| IPI00198904 | Mat2b Methionine adenosyltransferase 2 subunit beta | 48 | 37750 | 4 |
| IPI00200041 | Pbld Phenazine biosynthesis-like domain-containing protein | 48 | 32355 | 4 |
| IPI00565228 | Pms1 Protein | 48 | 106408 | 3 |
| IPI00362537 | RGD1309188 hypothetical protein LOC315463 | 48 | 30198 | 1 |
| IPI00359123 | RGD1310427 111 kDa protein | 48 | 113448 | 16 |
| IPI00476955 | RGD1564956 calcium binding protein P22-like | 48 | 22434 | 4 |
| IPI00206010 | Rras Harvey rat sarcoma virus oncogene, subgroup R | 48 | 24324 | 3 |
| IPI00475480 | Sfn similar to 14-3-3 protein sigma | 48 | 37822 | 5 |
| IPI00764841 | Slc25a31 similar to solute carrier family 25 (mitochondrial carrier; adenine nucleotide translocator), member 31 | 48 | 36263 | 8 |
| IPI00372717 | Spta1 Erythroid spectrin alpha | 48 | 284192 | 9 |
| IPI00471762 | Sypl1 Uncharacterized protein | 48 | 28952 | 3 |
| IPI00421328 | Usp40 LRRGT00071 | 48 | 91741 | 4 |
| IPI00196661 | Ywhaq 14-3-3 protein theta | 48 | 28578 | 5 |
| IPI00230857 | Ak2 Isoform 1 of Adenylate kinase 2, mitochondrial | 47 | 27223 | 12 |
| IPI00196994 | Arhgdia Rho GDP-dissociation inhibitor 1 | 47 | 24210 | 3 |
| IPI00471540 | Bhmt2 Betaine--homocysteine S-methyltransferase 2 | 47 | 40591 | 3 |
| IPI00780349 | Crocc ciliary rootlet coiled-coil | 47 | 212132 | 8 |
| IPI00362395 | Esd S-formylglutathione hydrolase | 47 | 32203 | 3 |
| IPI00231146 | Hint1;Hint1-ps1 Histidine triad nucleotide-binding protein 1 | 47 | 13882 | 3 |
| IPI00364061 | Hnrnpl heterogeneous nuclear ribonucleoprotein L isoform b | 47 | 64550 | 10 |
| IPI00421408 | LOC317165 Uncharacterized protein | 47 | 60105 | 4 |
| IPI00208236 | MGC94207 UPF0598 protein C8orf82 homolog | 47 | 24510 | 3 |
| IPI00213380 | Miox Inositol oxygenase | 47 | 33851 | 3 |
| IPI00212603 | Pnpo Pyridoxine-5~-phosphate oxidase | 47 | 30810 | 3 |
| IPI00212967 | RGD1306862 similar to CG6729-PA | 47 | 112144 | 3 |
| IPI00911245 | Rptor Uncharacterized protein | 47 | 152220 | 12 |
| IPI00213688 | Set Isoform 1 of Protein SET | 47 | 33386 | 4 |
| IPI00208324 | Slc13a3 Solute carrier family 13 member 3 | 47 | 67088 | 3 |
| IPI00475639 | Tubb2a Tubulin beta-2A chain | 47 | 50520 | 3 |
| IPI00205519 | Ugcgl1 UDP-glucose:glycoprotein glucosyltransferase 1 | 47 | 180577 | 11 |
| IPI00369311 | - 40 kDa protein | 46 | 41022 | 4 |
| IPI00361432 | - Uncharacterized protein | 46 | 25477 | 8 |
| IPI00214992 | Bard1 BRCA1-associated RING domain protein 1 | 46 | 86989 | 2 |
| IPI00188111 | Cct6a Chaperonin subunit 6a | 46 | 60002 | 4 |
| IPI00364286 | Cct7 chaperonin containing Tcp1, subunit 7 | 46 | 61426 | 4 |
| IPI00196478 | Clpx ATP-dependent Clp protease ATP-binding subunit clpX-like, mitochondrial | 46 | 71186 | 6 |
| IPI00206224 | Dars Aspartyl-tRNA synthetase, cytoplasmic | 46 | 57546 | 6 |
| IPI00209854 | Ddx20 56 kDa protein | 46 | 56851 | 2 |
| IPI00209690 | Ephx1 Epoxide hydrolase 1 | 46 | 54053 | 6 |
| IPI00201564 | Fmo1 Dimethylaniline monooxygenase [N-oxide-forming] 1 | 46 | 61249 | 8 |
| IPI00555168 | Fth1 Ferritin heavy chain | 46 | 21672 | 3 |
| IPI00210264 | Gsto1 Glutathione S-transferase omega-1 | 46 | 27936 | 8 |
| IPI00421429 | Jup Junction plakoglobin | 46 | 83125 | 1 |
| IPI00389571 | Krt8 Keratin, type II cytoskeletal 8 | 46 | 55490 | 4 |
| IPI00765714 | LOC683649 similar to type II keratin Kb36 | 46 | 26434 | 4 |
| IPI00324047 | Lypla1 Acyl-protein thioesterase 1 | 46 | 25165 | 4 |
| IPI00231106 | Mpst 3-mercaptopyruvate sulfurtransferase | 46 | 33393 | 5 |
| IPI00188245 | Mtch1 Mtch1 protein | 46 | 42296 | 2 |
| IPI00196167 | Mt-nd1 NADH-ubiquinone oxidoreductase chain 1 | 46 | 36454 | 2 |
| IPI00212052 | Ndufs4 NADH dehydrogenase [ubiquinone] iron-sulfur protein 4, mitochondrial | 46 | 20373 | 4 |
| IPI00192160 | Pllp Plasmolipin | 46 | 19906 | 3 |
| IPI00205107 | Prodh2 Probable proline dehydrogenase 2 | 46 | 51443 | 3 |
| IPI00358845 | Prosc proline synthetase co-transcribed homolog | 46 | 30817 | 2 |
| IPI00372631 | RGD1308139 hypothetical protein LOC360563 | 46 | 72236 | 5 |
| IPI00870032 | RGD1563438 hypothetical protein LOC287442 | 46 | 11882 | 2 |
| IPI00958176 | Rps2 Ribosomal protein S2 | 46 | 28860 | 9 |
| IPI00365946 | Sarnp SAP domain-containing ribonucleoprotein | 46 | 23647 | 6 |
| IPI00369358 | Sec24a SEC24 family, member A | 46 | 120126 | 2 |
| IPI00200593 | Serpina3k Serine protease inhibitor A3K | 46 | 47822 | 1 |
| IPI00327694 | Slc25a1 Tricarboxylate transport protein, mitochondrial | 46 | 34631 | 8 |
| IPI00769246 | Sptbn4 similar to spectrin beta 4 | 46 | 297163 | 15 |
| IPI00362757 | Vil1 Vil1 protein | 46 | 95011 | 10 |
| IPI00562540 | - 33 kDa protein | 45 | 34459 | 6 |
| IPI00208495 | - 33 kDa protein | 45 | 34988 | 4 |
| IPI00764788 | - similar to pyruvate kinase 3 isoform 1 | 45 | 49089 | 4 |
| IPI00948663 | Acsl5 77 kDa protein | 45 | 79016 | 5 |
| IPI00373674 | Agk acylglycerol kinase | 45 | 47986 | 6 |
| IPI00190701 | Apoe Apolipoprotein E | 45 | 36247 | 1 |
| IPI00360930 | Car1 Carbonic anhydrase 1 | 45 | 29013 | 1 |
| IPI00563501 | Dst 824 kDa protein | 45 | 847514 | 29 |
| IPI00204303 | Gpc2 Glypican-2 | 45 | 63788 | 4 |
| IPI00211895 | Gss Glutathione synthetase | 45 | 53258 | 6 |
| IPI00211897 | Gsta1;Gsta3;Gsta2 Glutathione S-transferase alpha-5 | 45 | 25388 | 13 |
| IPI00387868 | Hspa4 Heat shock 70 kDa protein 4 | 45 | 97438 | 7 |
| IPI00371173 | Mlec Malectin | 45 | 33129 | 3 |
| IPI00421428 | Pgam1 Phosphoglycerate mutase 1 | 45 | 29588 | 4 |
| IPI00231929 | Pkm2 Isoform M1 of Pyruvate kinase isozymes M1/M2 | 45 | 59329 | 4 |
| IPI00370450 | Plxnb2 plexin B2 | 45 | 209818 | 5 |
| IPI00369195 | Ppa2 pyrophosphatase (inorganic) 2 | 45 | 39065 | 2 |
| IPI00195614 | Rdh2 Retinol dehydrogenase 2 | 45 | 36219 | 4 |
| IPI00392019 | Rdh5 similar to retinol dehydrogenase 5 | 45 | 27745 | 2 |
| IPI00568360 | RGD1564131 similar to solute carrier family 25, member 5 | 45 | 29729 | 7 |
| IPI00390343 | Rpl6 60S ribosomal protein L6 | 45 | 35691 | 4 |
| IPI00371518 | Sdhc Uncharacterized protein | 45 | 18419 | 2 |
| IPI00393781 | Slc5a11 Protein | 45 | 49279 | 4 |
| IPI00210532 | Tagln3 Transgelin-3 | 45 | 22657 | 5 |
| IPI00421706 | Ugt1a2;Ugt1a1 UDP-glucuronosyltransferase 1-2 precursor | 45 | 61411 | 4 |
| IPI00214436 | Ugt1a7c;Ugt1a1 UGT1A7 | 45 | 60846 | 4 |
| IPI00213538 | Cpt1a Carnitine O-palmitoyltransferase 1, liver isoform | 44 | 88810 | 11 |
| IPI00195593 | Cpt2 Carnitine O-palmitoyltransferase 2, mitochondrial | 44 | 75569 | 2 |
| IPI00195516 | Hpx Hemopexin | 44 | 52350 | 4 |
| IPI00358998 | LOC680700 ribosomal protein L10a-like | 44 | 25239 | 4 |
| IPI00765290 | LOC681227;LOC100363700 probable N-acetyltransferase CML4-like | 44 | 25762 | 9 |
| IPI00764950 | LOC685322 ubiquinol-cytochrome c reductase complex 7.2kDa protein | 44 | 7457 | 1 |
| IPI00366206 | Ndufa12 NADH dehydrogenase (ubiquinone) 1 alpha subcomplex, 12 | 44 | 17224 | 7 |
| IPI00957897 | RGD1564698 ribosomal protein S10-like | 44 | 32288 | 7 |
| IPI00421626 | Rps9 40S ribosomal protein S9 | 44 | 22635 | 7 |
| IPI00371711 | Clic2 Chloride intracellular channel protein 2 | 43 | 28429 | 5 |
| IPI00464785 | Ctsa Uncharacterized protein | 43 | 51867 | 9 |
| IPI00766463 | LOC681996 RCG20659, isoform CRA_b | 43 | 38365 | 3 |
| IPI00372009 | Map1b Uncharacterized protein | 43 | 270675 | 20 |
| IPI00207980 | Rpl23 60S ribosomal protein L23 | 43 | 14970 | 3 |
| IPI00200552 | Rpl26 60S ribosomal protein L26 | 43 | 17267 | 3 |
| IPI00204344 | Bckdk [3-methyl-2-oxobutanoate dehydrogenase [lipoamide]] kinase, mitochondrial | 42 | 46673 | 3 |
| IPI00202513 | Ces2a Uncharacterized protein | 42 | 62162 | 6 |
| IPI00200465 | Ces2c Carboxylesterase 2 | 42 | 62662 | 4 |
| IPI00359436 | Ces2g Uncharacterized protein | 42 | 63210 | 6 |
| IPI00781693 | Dync2h1 Uncharacterized protein (Fragment) | 42 | 142247 | 11 |
| IPI00366767 | Fam46b Protein FAM46B | 42 | 47442 | 3 |
| IPI00608161 | LOC679149 LOC679149 protein | 42 | 63170 | 5 |
| IPI00763603 | LOC679368 carboxylesterase 5-like | 42 | 59695 | 10 |
| IPI00364064 | RGD1565965 low molecular weight phosphotyrosine protein phosphatase-like | 42 | 22424 | 2 |
| IPI00421616 | Acsm3 Acyl-coenzyme A synthetase ACSM3, mitochondrial | 41 | 66241 | 7 |
| IPI00949697 | Chdh Uncharacterized protein (Fragment) | 41 | 16822 | 8 |
| IPI00214394 | Csad Cysteine sulfinic acid decarboxylase | 41 | 55841 | 8 |
| IPI00372377 | Fmo6 flavin containing monooxygenase 3-like | 41 | 60761 | 5 |
| IPI00211336 | Sh3bgrl3 Uncharacterized protein | 41 | 10527 | 3 |
| IPI00655276 | Tjp2 Uncharacterized protein | 41 | 131687 | 17 |
| IPI00870417 | Cpxm1 Uncharacterized protein | 40 | 81429 | 2 |
| IPI00471666 | Fam151a Protein FAM151A | 40 | 67544 | 9 |
| IPI00210542 | Gsta4 Glutathione S-transferase alpha-4 | 40 | 25550 | 11 |
| IPI00391161 | LOC501110 Glutathione S-transferase A6 | 40 | 25791 | 9 |
| IPI00958667 | LOC692029 ubiquitin and ribosomal protein L40-like | 40 | 19944 | 8 |
| IPI00372489 | Mta2 Uncharacterized protein | 40 | 75654 | 11 |
| IPI00475911 | RGD1564290 ribosomal protein S27a-like | 40 | 18228 | 5 |
| IPI00779483 | Rnf135 Mitochondrial Rho GTPase | 40 | 82011 | 5 |
| IPI00373761 | Tfap2b Uncharacterized protein | 40 | 48476 | 6 |
| IPI00213190 | Bcat2 Branched-chain-amino-acid aminotransferase, mitochondrial | 39 | 44817 | 2 |
| IPI00214398 | Mosc2 MOSC domain-containing protein 2, mitochondrial | 39 | 38851 | 10 |
| IPI00382222 | Syncrip Heterogeneous nuclear ribonucleoprotein Q | 39 | 59845 | 4 |
| IPI00476086 | Atp6v0d1 Uncharacterized protein | 38 | 40731 | 5 |
| IPI00765407 | Fam193a hypothetical protein | 38 | 158541 | 8 |
| IPI00763565 | LOC679594;LOC682397 ubiquitin B-like | 38 | 8821 | 10 |
| IPI00209570 | Me2 NAD-dependent malic enzyme, mitochondrial | 38 | 65994 | 10 |
| IPI00208265 | Ppp1ca Serine/threonine-protein phosphatase PP1-alpha catalytic subunit | 38 | 38229 | 3 |
| IPI00950668 | Prmt2 Uncharacterized protein (Fragment) | 38 | 49279 | 4 |
| IPI00627068 | Sfpq Splicing factor proline/glutamine rich | 38 | 75553 | 14 |
| IPI00362078 | Bcl6 Uncharacterized protein | 37 | 80582 | 6 |
| IPI00369144 | Frrs1 Uncharacterized protein | 37 | 67063 | 6 |
| IPI00766956 | Hist1h1b Uncharacterized protein | 37 | 22635 | 9 |
| IPI00382234 | LOC367195 Uncharacterized protein | 37 | 15671 | 4 |
| IPI00202762 | Prl2b1 Prolactin-2B1 | 37 | 26328 | 2 |
| IPI00779993 | RGD1564425 Uncharacterized protein | 37 | 23316 | 3 |
| IPI00368902 | RGD1564469 RPLP0 protein-like | 37 | 34315 | 6 |
| IPI00369429 | RGD1564906 glutathione S-transferase alpha-3-like | 37 | 28631 | 11 |
| IPI00209832 | Acot5 Uncharacterized protein | 36 | 46807 | 9 |
| IPI00205559 | Crtac1 cartilage acidic protein 1 | 36 | 71152 | 5 |
| IPI00212708 | Fetub Uncharacterized protein | 36 | 44054 | 6 |
| IPI00958036 | LOC680155 Uncharacterized protein | 36 | 186889 | 9 |
| IPI00194093 | RGD1563958 Uncharacterized protein | 36 | 15964 | 9 |
| IPI00210946 | Rpl17 60S ribosomal protein L17 | 36 | 21611 | 4 |
| IPI00958229 | Dnahc3l dynein, axonemal, heavy chain 3-like | 35 | 475213 | 38 |
| IPI00388081 | Esco1 N-acetyltransferase ESCO1 | 35 | 96041 | 7 |
| IPI00763051 | Fam40a family with sequence similarity 40, member A isoform 3 | 35 | 96290 | 7 |
| IPI00393683 | Foxp4 Uncharacterized protein | 35 | 75963 | 3 |
| IPI00565779 | LOC678705;LOC100364565 Uncharacterized protein | 35 | 24888 | 8 |
| IPI00192257 | Rpl18a 60S ribosomal protein L18a | 35 | 21004 | 3 |
| IPI00364983 | Sec61b protein transport protein Sec61 subunit beta | 35 | 10039 | 6 |
| IPI00361275 | Tanc2 tetratricopeptide repeat, ankyrin repeat and coiled-coil containing 2-like | 35 | 221157 | 21 |
| IPI00390944 | - Uncharacterized protein | 34 | 33696 | 6 |
| IPI00869717 | Acss3 Uncharacterized protein | 34 | 75255 | 10 |
| IPI00515830 | Ep300 E1A binding protein p300 | 34 | 270252 | 12 |
| IPI00214587 | Fdx1 Adrenodoxin, mitochondrial | 34 | 20578 | 5 |
| IPI00324023 | Fmr1 Isoform 2 of Fragile X mental retardation protein 1 homolog | 34 | 65876 | 8 |
| IPI00767755 | LOC684611 centromere protein H-like | 34 | 27629 | 1 |
| IPI00190161 | Ndufc2 NADH dehydrogenase [ubiquinone] 1 subunit C2 | 34 | 14406 | 3 |
| IPI00191502 | Psma5 Proteasome subunit alpha type-5 | 34 | 26545 | 3 |
| IPI00769110 | Rrbp1 ribosome binding protein 1 isoform 3 | 34 | 157865 | 22 |
| IPI00213559 | Rtn1 Isoform RTN1-B of Reticulon-1 | 34 | 83350 | 3 |
| IPI00363930 | Sept11 Isoform 1 of Septin-11 | 34 | 50005 | 8 |
| IPI00366575 | Suv420h2 Histone-lysine N-methyltransferase SUV420H2 | 34 | 54713 | 9 |
| IPI00200053 | Atp5hl1 Uncharacterized protein | 33 | 18724 | 3 |
| IPI00231690 | Csrp1 Cysteine and glycine-rich protein 1 | 33 | 21455 | 3 |
| IPI00204941 | Dnaja3 Tid-1 long isoform | 33 | 53051 | 4 |
| IPI00959923 | LOC500210 high-mobility group box 1-like | 33 | 23730 | 2 |
| IPI00454327 | LOC500350 LRRGT00139 | 33 | 46612 | 7 |
| IPI00959070 | LOC680988 ribosomal protein S12-like | 33 | 37220 | 7 |
| IPI00764492 | LOC687346 CG5555-like | 33 | 68074 | 7 |
| IPI00558381 | Mark2 Uncharacterized protein | 33 | 83420 | 11 |
| IPI00362621 | - Uncharacterized protein | 32 | 77373 | 5 |
| IPI00195860 | Cox7a2l2;Cox7a2 Cytochrome c oxidase subunit 7A2, mitochondrial | 32 | 9347 | 5 |
| IPI00193247 | Cse1l Uncharacterized protein | 32 | 110942 | 22 |
| IPI00371542 | Disp1 Uncharacterized protein | 32 | 172777 | 7 |
| IPI00363523 | Fam65a family with sequence similarity 65, member A | 32 | 146811 | 9 |
| IPI00211698 | Flii Uncharacterized protein | 32 | 146191 | 8 |
| IPI00957256 | LOC684016 liver carboxylesterase 1-like | 32 | 35083 | 5 |
| IPI00867940 | Med23 mediator complex subunit 23 isoform 2 | 32 | 159037 | 18 |
| IPI00206879 | Mrpl16 39S ribosomal protein L16, mitochondrial | 32 | 28990 | 6 |
| IPI00204076 | Nradd P75-like apoptosis-inducing death domain protein short isoform | 32 | 19776 | 12 |
| IPI00370457 | RGD1564209 acyl-Coenzyme A dehydrogenase family, member 8 | 32 | 10855 | 5 |
| IPI00364198 | RGD1565048 ribosomal protein L9-like | 32 | 21863 | 3 |
| IPI00190377 | Taldo1 Transaldolase | 32 | 37608 | 11 |
| IPI00470288 | Ckb Creatine kinase B-type | 31 | 42983 | 10 |
| IPI00767028 | Dnah7 Dynein heavy chain 7, axonemal | 31 | 467909 | 34 |
| IPI00370475 | Dopey1 Uncharacterized protein | 31 | 278474 | 13 |
| IPI00957927 | Dopey2 dopey family member 2 isoform 1 | 31 | 252813 | 18 |
| IPI00192806 | Gykl1 glycerol kinase-like 1 | 31 | 61066 | 11 |
| IPI00958881 | LOC687872 ribosomal protein S2-like | 31 | 27851 | 6 |
| IPI00364850 | Ndufb6 Uncharacterized protein | 31 | 15628 | 4 |
| IPI00209980 | Pmpcb Mitochondrial-processing peptidase subunit beta | 31 | 55029 | 2 |
| IPI00569584 | RGD1564597 ribosomal protein S25-like | 31 | 16126 | 7 |
| IPI00367215 | RGD1565912 ribosomal protein S18-like | 31 | 19424 | 3 |
| IPI00215184 | Rps25 40S ribosomal protein S25 | 31 | 13791 | 6 |
| IPI00475539 | Tdrd7 Uncharacterized protein (Fragment) | 31 | 124208 | 8 |
| IPI00390751 | - Uncharacterized protein | 30 | 24028 | 4 |
| IPI00911371 | Acad10 RGD1310159 protein | 30 | 120106 | 22 |
| IPI00189189 | Akr1c18 20alpha-hydroxysteroid dehydrogenase | 30 | 34757 | 6 |
| IPI00555261 | Akr1c2 Aldo-keto reductase family 1 member C21 | 30 | 36625 | 6 |
| IPI00230954 | Dync1i2 Isoform 2B of Cytoplasmic dynein 1 intermediate chain 2 | 30 | 70748 | 6 |
| IPI00204503 | Epb41l3 Type II brain 4.1 minor isoform | 30 | 107463 | 15 |
| IPI00324820 | Epcam Epithelial cell adhesion molecule | 30 | 35869 | 2 |
| IPI00231693 | Rps3a 40S ribosomal protein S3a | 30 | 30154 | 15 |
| IPI00373448 | - Uncharacterized protein | 29 | 18250 | 7 |
| IPI00389152 | Cox6b1 Uncharacterized protein | 29 | 10293 | 3 |
| IPI00949657 | Cpsf1 Uncharacterized protein | 29 | 155980 | 16 |
| IPI00210884 | Dnaja1 DnaJ homolog subfamily A member 1 | 29 | 45581 | 5 |
| IPI00361878 | Grb10 Uncharacterized protein | 29 | 62329 | 4 |
| IPI00382186 | LOC307347 Da1-6 | 29 | 264682 | 13 |
| IPI00382256 | LOC502176 Uncharacterized protein | 29 | 16980 | 4 |
| IPI00768308 | LOC687057 calponin 2-like | 29 | 27481 | 2 |
| IPI00566208 | LOC690976 Uncharacterized protein | 29 | 14255 | 2 |
| IPI00196723 | Chrna2 Neuronal acetylcholine receptor subunit alpha-2 | 28 | 59257 | 6 |
| IPI00360386 | Hnrnpul2 Uncharacterized protein | 28 | 85434 | 6 |
| IPI00566286 | LOC499735 heat shock protein 1, alpha-like | 28 | 35422 | 4 |
| IPI00196684 | Prkar2a cAMP-dependent protein kinase type II-alpha regulatory subunit | 28 | 45797 | 5 |
| IPI00366263 | RGD1564420 similar to Hypothetical protein MGC31278 | 28 | 121314 | 14 |
| IPI00566820 | RGD1564420 Uncharacterized protein | 28 | 82335 | 10 |
| IPI00368970 | RGD1565900 Uncharacterized protein | 28 | 15812 | 4 |
| IPI00213335 | Slc12a1 Uncharacterized protein | 28 | 121376 | 10 |
| IPI00372007 | Mrps36 28S ribosomal protein S36, mitochondrial | 27 | 11406 | 6 |
| IPI00324983 | Rps17;LOC100365810 40S ribosomal protein S17 | 27 | 15557 | 2 |
| IPI00190290 | Rras2 Uncharacterized protein | 27 | 23613 | 5 |
| IPI00327502 | Sftpa1 Pulmonary surfactant-associated protein A | 27 | 26671 | 6 |
| IPI00870450 | Dmrta1 Uncharacterized protein | 26 | 53083 | 2 |
| IPI00209789 | Fkbp3 peptidyl-prolyl cis-trans isomerase FKBP3 | 26 | 25220 | 6 |
| IPI00326703 | Gpx4 Uncharacterized protein | 26 | 22934 | 2 |
| IPI00368156 | Man2a2 Uncharacterized protein | 26 | 132400 | 10 |
| IPI00203358 | Ppp1cc Isoform Gamma-1 of Serine/threonine-protein phosphatase PP1-gamma catalytic subunit | 26 | 37701 | 3 |
| IPI00372387 | - Uncharacterized protein | 25 | 40197 | 5 |
| IPI00209216 | Add3 Isoform 2 of Gamma-adducin | 25 | 79097 | 5 |
| IPI00371036 | Fis1 Isoform 1 of Mitochondrial fission 1 protein | 25 | 17041 | 3 |
| IPI00957675 | Ppih Peptidyl-prolyl cis-trans isomerase | 25 | 14601 | 2 |
| IPI00202461 | Prpf18 Pre-mRNA-splicing factor 18 | 25 | 40012 | 5 |
| IPI00372471 | Rps6ka4 ribosomal protein S6 kinase, polypeptide 4 | 25 | 86026 | 10 |
| IPI00231196 | Tagln Transgelin | 25 | 22645 | 3 |
| IPI00476464 | Akr1c19 Uncharacterized protein | 24 | 37380 | 5 |
| IPI00209874 | Cpne7 Uncharacterized protein | 24 | 38050 | 4 |
| IPI00366464 | Cpne8 Uncharacterized protein | 24 | 65211 | 5 |
| IPI00364549 | Cpne9 Copine-9 | 24 | 62228 | 9 |
| IPI00206112 | Haus1 HAUS augmin-like complex subunit 1 | 24 | 31535 | 8 |
| IPI00464724 | Ppp3cc Serine/threonine-protein phosphatase | 24 | 59593 | 6 |
| IPI00949722 | Acss2 Acyl-CoA synthetase short-chain family member 2 | 23 | 79963 | 8 |
| IPI00393333 | Atic Bifunctional purine biosynthesis protein PURH | 23 | 64681 | 7 |
| IPI00191487 | Atp7a Copper-transporting ATPase 1 | 23 | 163415 | 13 |
| IPI00191112 | Ndufab1 Acyl carrier protein | 23 | 17788 | 3 |
| IPI00779473 | Serpinb1a Leukocyte elastase inhibitor A | 23 | 42871 | 4 |
| IPI00768759 | Exo1 Uncharacterized protein | 22 | 93395 | 9 |
| IPI00568632 | Mapt Microtubule-associated protein | 22 | 78792 | 11 |
| IPI00208657 | RGD1565117 Uncharacterized protein | 22 | 12169 | 4 |
| IPI00358704 | Dsc2 Uncharacterized protein | 21 | 101281 | 12 |
| IPI00949127 | Dsc3 Uncharacterized protein | 21 | 95082 | 8 |
| IPI00287309 | Gpm6a Uncharacterized protein | 21 | 31916 | 2 |
| IPI00958569 | LOC679539 ubiquitin-conjugating enzyme E2-like isoform 2 | 21 | 11775 | 9 |
| IPI00782322 | Map3k2 mitogen-activated protein kinase kinase kinase 2 | 21 | 69961 | 4 |
| IPI00763255 | E2f5 E2F transcription factor 5 | 20 | 41525 | 4 |
| IPI00363260 | LOC685890 serine/arginine repetitive matrix 3 | 20 | 71477 | 10 |
| IPI00194042 | Cox6a1 Cytochrome c oxidase subunit 6A1, mitochondrial | 19 | 12293 | 4 |
| IPI00190929 | Fam115a Uncharacterized protein | 19 | 103686 | 10 |
| IPI00777845 | LOC681766 hypothetical protein LOC681766 | 19 | 27995 | 1 |
| IPI00373587 | Ppp2r4 Uncharacterized protein | 19 | 36879 | 7 |
| IPI00365894 | Setd3 Setd3 protein | 19 | 67777 | 7 |
| IPI00371043 | Manf Mesencephalic astrocyte-derived neurotrophic factor | 18 | 20831 | 5 |
| IPI00358767 | Sh3gl2 Endophilin-A1 | 17 | 40045 | 6 |
| IPI00360508 | Dnah8 Uncharacterized protein | 16 | 545515 | 51 |
| IPI00390531 | Fsip2 fibrous sheath-interacting protein 2-like | 16 | 589252 | 30 |
| IPI00359003 | Macf1 Uncharacterized protein | 15 | 836127 | 65 |
